# Oceanic fronts shape hemispheric contrasts in polar stratospheric extremes

**DOI:** 10.1038/s41467-026-71998-5

**Published:** 2026-04-20

**Authors:** Nour-Eddine Omrani, Fumiaki Ogawa, Hisashi Nakamura, Sandro W. Lubis, Noel S. Keenlyside, Luca Famooss Paolini

**Affiliations:** 1 Geophysical Institute, University of Bergen and Bjerknes Centre for Climate Research, Bergen, Norway; 2 Faculty of Bioresources, Mie University, Tsu, Japan; 3 Research Center for Advanced Science and Technology, University of Tokyo, Tokyo, Japan; 4 Japan Agency for Marine-Earth Science and Technology, Yokohama, Japan; 5 Pacific Northwest National Laboratory, Richland, WA USA; 6 Nansen Environmental and Remote Sensing Center, Bergen, Norway; 7 Department of Physics and Astronomy “Augusto Righi”, University of Bologna, Bologna, Italy; 8 CMCC Foundation, Euro-Mediterranean Center on Climate Change, Bologna, Italy

**Keywords:** Atmospheric dynamics, Ocean sciences, Atmospheric chemistry

## Abstract

Sudden Stratospheric Warmings (SSWs) and Polar Stratospheric Clouds (PSCs) exhibit striking inter-hemispheric asymmetries: SSWs are frequent in the Arctic but rare in the Antarctic, while PSCs are more persistent in the Antarctic. Although land–sea thermal contrast and orography (LSCO) have been traditionally invoked to explain these asymmetries, here we show using semi-idealized model experiments that even though LSCO strongly impacts the mean state of SSWs and PSCs, it alone cannot fully account for the observed differences. Using model experiments, we reveal that midlatitude oceanic sea surface temperature (SST) fronts represent a crucial supporting additional driver of the hemispheric stratospheric differences. Like LSCO, SST fronts enhance stratospheric convergence of resolved waves, strengthening the Brewer–Dobson Circulation and inducing high-latitude adiabatic warming. This warming significantly enhances Arctic SSW frequency and strongly suppresses PSC formation. Sub-grid-scale non–resolved wave forcing modulates the stratospheric resolved waves effect. The oceanic impact is dominated by North Pacific SST fronts. Our results highlight the indispensable role of SST fronts in shaping Arctic–Antarctic asymmetries in stratospheric dynamics and associated extremes.

## Abstract (web summary)

The study shows that midlatitude oceanic fronts, alongside land–sea contrast and orography, play a crucial role in the observed Arctic–Antarctic differences in the occurrence of sudden stratospheric warmings and polar stratospheric clouds, by enhancing wave activity, strengthening the Brewer–Dobson circulation, and weakening the Arctic stratospheric polar vortex.

## Introduction

**Sudden stratospheric warmings (SSWs)** are dramatic disruptions of the cold polar vortex marked by a strong and rapid temperature rise in the polar stratosphere and a reversal of the polar-night jet from westerly to easterly winds^1,2^. These events predominantly occur during Northern Hemisphere (NH) winters, with an observed climatological frequency of about 6 events per decade^3^, but are rare in the Southern Hemisphere (SH), where only one major SSW has been observed in 2002, along with minor events in 2019 and 2024^4–8^. The stratospheric warming and associated vortex weakening significantly impact surface climate by driving severe cold spells and altering large-scale tropospheric and oceanic circulation patterns^2,9–12^.

**Polar Stratospheric Clouds (PSCs)**, primarily forming in the frigid winter polar stratosphere, are critical for stratospheric ozone depletion. They are categorized as Type I, composed of nitric acid trihydrate (NAT) and appearing thin and wispy, and Type II, made of water-ice and displaying iridescent ‘mother-of-pearl’ colors^13–16^. PSC surfaces facilitate chemical reactions that convert inert chlorine and bromine compounds into reactive free radicals, which catalyze ozone destruction upon springtime sunlight exposure. This process significantly contributes to the ozone-hole^17–19^, increasing harmful surface UV radiation and threatening both human health and ecosystems^20^. PSCs display pronounced hemispheric asymmetry. In the Antarctic, both PSC types are more widespread and persist longer than in the Arctic. The warmer Arctic stratosphere rarely supports Type II PSCs, which require extremely cold conditions, while Type I PSCs in the Arctic are less frequent and shorter-lived compared to their Antarctic counterparts^21^. Consequently, ozone holes are more frequent, persistent, severe, and deeper in the Antarctic than in the Arctic^22^. Antarctic-like ozone holes are exceptionally rare in the Arctic—having occurred only in 2011 and 2020^23–25^—mirroring the fact that SSWs are rare and virtually absent in the Antarctic.

The SSWs and PSCs phenomena are closely linked to the Brewer-Dobson Circulation (BDC), a fundamental stratospheric overturning circulation transporting air, trace-gases, and aerosols from the tropics to polar regions^20,26–29^. The BDC strongly regulates the polar stratospheric temperatures and is typically stronger in the NH than in the SH winter^26,28^. The wave-mean flow interactions constitute the main driving mechanisms of both BDC and polar stratospheric background wind^26,28–32^. The stronger BDC in the NH is associated with greater polar adiabatic warming and weaker polar vortex, suppressing PSC-formation and enabling frequent SSWs. Conversely, the SH’s weaker BDC is associated with enhanced polar stratospheric cooling and a stronger polar vortex, favoring PSC formation and limiting SSW occurrence. The convergence of the wave forcing (Methods, ^[Eq.1^, $\frac{1}{({{{\rm{\rho }}}}_{0}\cos \phi )}\nabla \cdot {{\bf{F}}}{{ < }}{{\bf{0}}}$) in the wintertime NH extratropical stratosphere is balanced by Coriolis forcing ($f{\bar{{{\rm{v}}}}}^{*} > 0$) resulting in poleward residual circulation (${\bar{{{\rm{v}}}}}^{*} > 0$) with an upward (downward) branch of BDC and adiabatic cooling (warming) in tropical (mid-to-high) latitudes^26,28^. The adiabatic warming in the downward branch of BDC acts to offset the wintertime strong radiative cooling induced by the lack of solar radiation. The inherent dynamics of the SSWs and PSCs, therefore, require a dynamical understanding of what controls the BDC itself, particularly the planetary wave activity and wave–mean flow interactions. While our primary focus is on stratospheric response and its dynamics, the broader implications of this work extend to surface climate, ozone chemistry, and model fidelity

What drives the North-South asymmetry in SSWs and PSCs? Traditionally, the interhemispheric asymmetry in SSWs and PSCs has been attributed to orography and land–sea thermal contrast, which influence planetary wave amplitudes and their propagation into the stratosphere^33,34^. As we will show, these factors alone cannot fully explain the observed asymmetry, suggesting additional mechanisms may be at play.

Here, we propose a crucial missing link: Sea Surface-Temperature (SST) fronts. These sharp oceanic temperature gradients, associated with western boundary currents, play important roles in atmospheric dynamics^35–40^. While previous studies have explored the role of SST-fronts in shaping the tropospheric eddy-driven jets, storm-tracks, blocking events and stratospheric polar vortex, their specific impacts on SSWs, the BDC, and PSCs remain largely unexplored^35–38,41^.

The goal of this study is to identify which large-scale lower boundary structures—specifically land–sea thermal contrast, orography, and oceanic SST fronts—are responsible for the hemispheric asymmetry in SSW frequency and potential formation of PSC. This asymmetry is reproducible even in models with standard horizontal resolution, provided that the stratosphere is well-resolved and gravity-wave parameterizations are included (Supplementary Figs. 5–7). We focus in particular on the influence of Northern Hemisphere ocean western boundary currents (OWBCs) and LSCO on stratospheric circulation. OWBCs, such as the Kuroshio and Gulf Stream, generate sharp meridional SST gradients (“SST fronts”) through the convergence of heat transported by subtropical and subpolar ocean gyres. These SST fronts are hypothesized to play a critical complementary role, alongside LSCO, in modulating the hemispheric asymmetry in stratospheric extremes.

## Results

To explore how SST fronts influence SSWs, PSCs, and the BDC in comparison to other terrestrial conditions, we conducted a series of semi-idealized model experiments. These experiments systematically change the boundary conditions to isolate the contributions of specific factors (Methods, Supplementary Table S1 and Supplementary Fig. 1). The Aqua-Planet (AP) configurations AP_NoFr and AP_Fr mimic the SH-like conditions without and with SST fronts, respectively. In AP_Fr, a zonally symmetric SST-front is added to the AP_NoFr configuration, while in LSCO_NoFr the land–sea thermal contrast and orography (LSCO) is added to the AP_NoFr to simulate NH-like conditions without SST fronts. We also conducted the LSCO_Fr-experiment by adding realistic mid-latitude SST fronts in both the Atlantic and Pacific basins to the LSCO_NoFr configuration. Additionally, basin-specific contributions are examined through LSCO_AtFr and LSCO_PaFr experiments, in which realistic SST fronts are added separately in the Atlantic and Pacific basins, allowing the effects of each basin on stratospheric dynamics to be distinguished. The goal of this study is not to assess the impact of fine-scale SST gradient structures, but rather to understand how the presence or absence of the NH large-scale SST frontal zones—represented at standard model resolution—contributes to the observed hemispheric asymmetry in SSW and PSC frequency that is already captured by stratosphere-resolving models with standard resolution (Supplementary Figs. 2, 3 and Supplementary Figs. 5, 6).

Our motivation and experimental design are grounded in well-established dynamical theories, particularly linear baroclinic instability theory and the Charney–Drazin criterion^42–44^. According to the former theory, oceanic SST fronts enhance near-surface baroclinicity, strengthening baroclinic eddies along the midlatitude storm tracks and the eddy-driven westerly jets. In the Pacific, the strengthening of the eddy-driven jet (e.g., ref. ^35^.) deepens the high-latitude low-pressure (Supplementary Fig. 9), which acts as a well-known precursor for upward wave propagation into the stratosphere and SSWs^45–48^. The Charney–Drazin criterion further constrains planetary wave propagation into the stratosphere, allowing vertical propagation only when the zonal-mean wind is westerly and not excessively strong. This condition is met more readily when the strong wintertime background westerlies are weakened by LSCO (e.g.,34), creating a favorable environment for upward wave propagation into the stratosphere. In contrast, the Charney–Drazin criterion implies that in aqua-planet configurations, the strong zonally symmetric background westerlies should suppress the upward wave propagation, which can explain the strong polar vortex in the SH even in the presence of SST fronts. These theoretical principles provide a physical framework for the expected results and for interpreting the differences in stratospheric responses to SST fronts across the model configurations.

### Impact of LSCO and SST Fronts on PSCs and SSWs

**Fig. 1:**
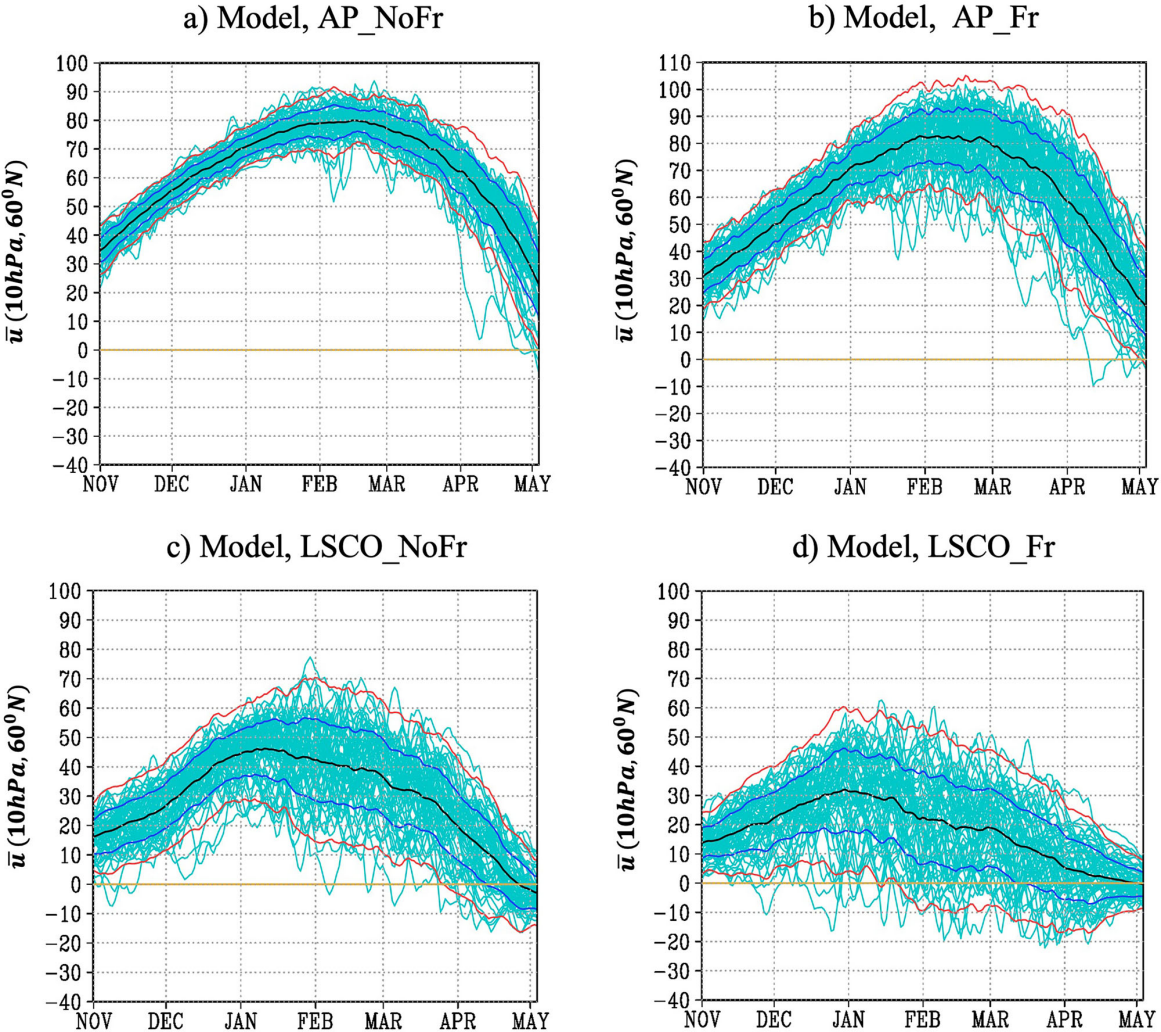
November (NOV) to April (APR) evolution of the zonally averaged stratospheric zonal wind (10 hPa, 60°N) in model experiments. The panels depict the NOV-APR evolution on daily timescales of the zonally averaged westerly wind ($\bar{u}$) at 10 hPa and 60°N across different model configurations. Panel (**a**) represents the Aqua-Planet (AP) experiment without a sea surface temperature (SST) front (AP_NoFr). Panel (**b**) shows the AP experiment with zonally averaged SST fronts (AP_Fr), to mimic Southern Hemisphere (SH) conditions. Panel (**c**) illustrates the experiment where land–sea thermal contrast and orography (LSCO) were added to the AP_NoFr configuration, while keeping the SST front absent (LSCO_NoFr). Panel (**d**) corresponds to the experiment where realistic midlatitude SST fronts were introduced in the North Atlantic and Pacific basins, alongside LSCO (LSCO_Fr). The black lines indicate the mean seasonal cycle, while the blue and red lines represent the ± 1 and ± 2 standard deviation margins, respectively. The dark yellow horizontal line denotes the zero-wind threshold, indicating the transition from westerly to easterly winds, which leads to the occurrence of Sudden Stratospheric Warmings (SSWs).

**Fig. 2:**
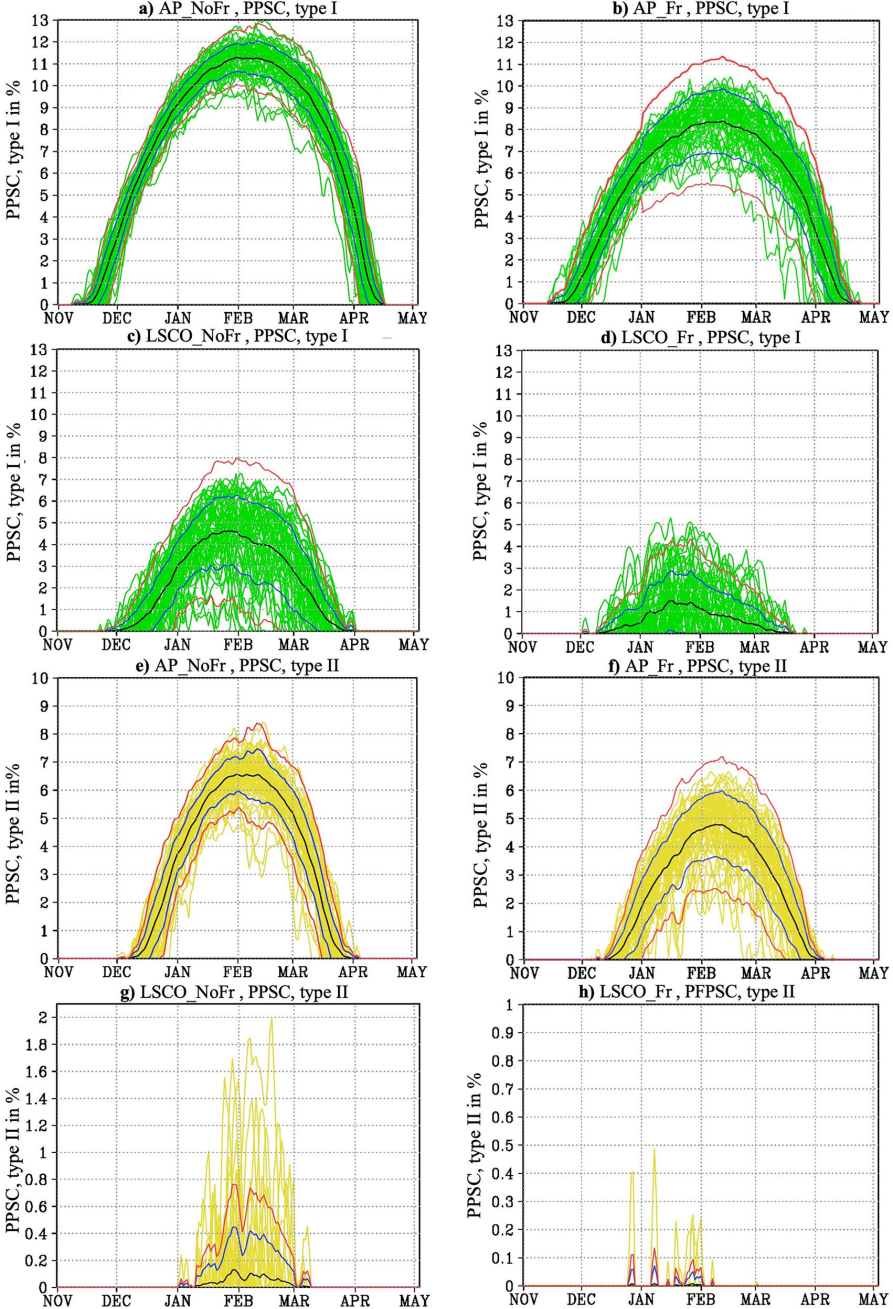
November (NOV) to April (APR) evolution of PPSC type I and II (model experiments, Methods). The panels illustrate the NOV-APR evolution of the Potential for Polar Stratospheric Clouds (PPSC) on daily timescales. Panels (**a**–**d**) depict PPSC type I, defined as the percentage of the Northern Hemisphere area where temperatures fall below 195 K, which is conducive to the formation of nitric acid trihydrate (NAT) clouds. Panel (**a**) corresponds to the Aqua-Planet (AP) experiment without sea surface temperature (SST) fronts (AP_NoFr, no SST-fronts configuration), panel (**b**) represents the AP experiment with SST fronts (AP_Fr), panel (**c**) shows the land–sea thermal contrast and orography (LSCO) configuration without SST fronts (LSCO_NoFr, no SST-fronts configuration), and panel (**d**) depicts the LSCO configuration with SST-fronts (LSCO_Fr). Panels (**e**–**h**) follow the same configuration as above but display PPSC type II, defined by the area where temperatures fall below 188 K, conducive to the formation of water ice clouds. The black lines indicate the mean seasonal cycles, while the blue and red lines represent the ± 1 and ± 2 standard deviation margins, respectively.

We first consider the zonally averaged zonal wind at 60°N and 10hPa, alongside the conditions conducive to PSC-formation—quantified by the potential of PSC-formation (PPSC, Methods)—for different experimental setups (Figs. 1, 2) and NCEP-reanalysis (Supplementary Fig. 2, 3). In the AP_NoFr experiments (Fig. 1a), the westerlies remain overall stable throughout winter, and no major SSW events—characterized by a reversal of westerlies (“Methods”, Supplementary Table S1)—are observed in the model simulation. These conditions correspond to the highest PPSC values (Fig. 2a, e), indicating the most favorable climatic environment for PSC-formation. Adding mid-latitude SST fronts to the aqua-planet configuration increases the variability of zonally averaged wind and PPSC (Figs. 1b, 2b, f), but conditions remain unfavorable for SSWs and highly conducive to PSCs. These conditions closely resemble the observed SH conditions (Supplementary Figs. 2a, 3a, c), characterized by a stable and intensely cold polar vortex.

The LSCO_NoFr-experiment shows that adding land–sea thermal contrast and orography to the AP_NoFr configuration markedly weakens the westerlies and increases their variability (Fig. 1a, c). The climatic conditions favorable for Type I PPSCs (PPSC-I) are also significantly reduced compared to AP_NoFr (Fig. 2a, c), while conditions for Type II PPSCs (PPSC-II) become relatively rare (Fig. 2g). These changes in westerly winds and PPSC begin to approach the NH-like conditions, but they still fall short of fully replicating the observed PPSC and SSW frequency values (Supplementary Figs. 2b, 3b, d and Supplementary Table S1). In fact, the SSW frequency remains exceptionally low compared to observational and reanalysis data (with about 1 event per decade compared to 6 events^3^).

The addition of mid-latitude SST fronts to the LSCO_NoFr configuration further weakens the stratospheric westerlies and enhances their variability considerably, leading to much more frequent SSWs (about 9 events per decade, Fig. 1d). This shift dramatically decreases PPSC-I (Fig. 2d) and virtually eliminates PPSC-II (Fig. 2h). The conditions simulated in the LSCO_Fr-experiment align best with the observed Arctic conditions based on the reanalysis data (Supplementary Figs. 2b3b, d), although the simulated SSW frequency remains somewhat higher and the PPSC-II values are lower than observed. These discrepancies may stem from the exclusion of Arctic sea-ice, tropical SST-asymmetry, and active two-way atmosphere-ocean interaction in our front experiments (“Methods”), as well as potential model biases. For instance, Arctic sea-ice melting acts to weaken and warm the NH stratospheric polar vortex further^49^, while the inclusion of atmosphere-ocean-coupling can introduce enhanced decadal-to-multidecadal SSW-variability^50,51^. Such variability includes periods of both high and low SSW frequency, resulting in a more balanced average frequency over longer time periods. Despite these limitations, the experiments reveal the critical role of mid-latitude SST fronts in shaping polar stratospheric dynamics and associated SSW frequency and PPSC. To further evaluate the contributions of the SST fronts, we analyzed basin-specific impacts. The examination of basin-specific contributions of SST fronts (Supplementary Fig. 4 and Supplementary Table S1) indicates that the Pacific SST front is the primary driver of the overall stratospheric response, significantly affecting both SSW frequency and conditions conducive to PSC-formation.

### Dynamics of the response

**Fig. 3:**
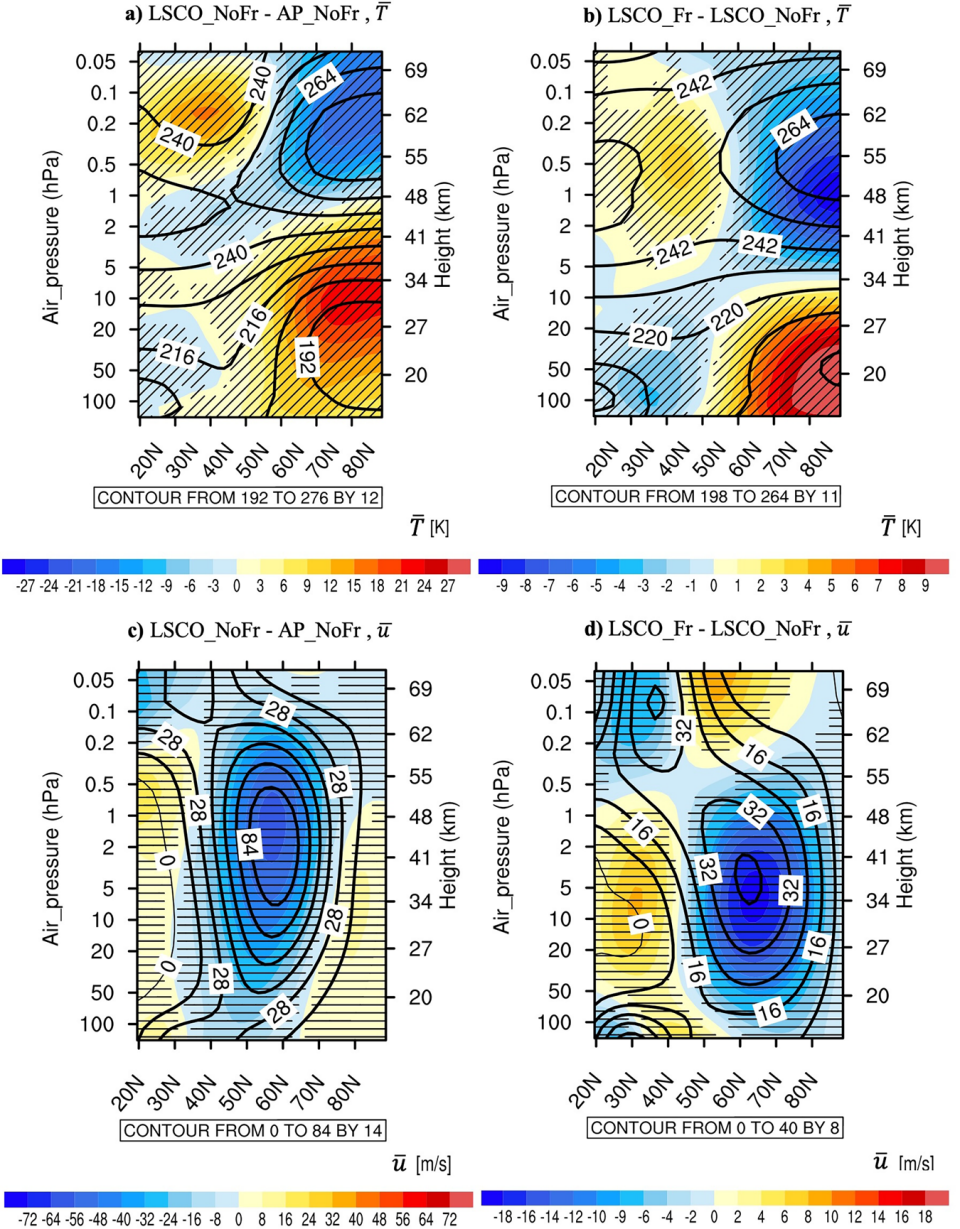
January–March (JFM)-mean response of the zonally averaged temperature and zonally averaged zonal wind. Panels (**a**, **b**) show the JFM climatology of the zonally averaged temperature differences between (**a**) LSCO_NoFr and AP_NoFr, and (**b**) LSCO_Fr and LSCO_NoFr, highlighting the impacts of land–sea thermal contrast and orography (LSCO), and sea surface temperature (SST) fronts, respectively. Panels (**c**, **d**) display the corresponding zonally averaged zonal wind differences (positive for anomalous westerlies) for (**c**) LSCO_NoFr - AP_NoFr, and (**d**) LSCO_Fr - LSCO_NoFr. The hatching indicates regions where the differences are statistically significant, based on a two-tailed Student’s *t* test at the 95% confidence level. The solid contours represent the control simulation AP_NoFr in panels (**a**, **c**), and the control simulation LSCO_NoFr in panels (**b**, **d**). Note: Color bars differ between panels (**a**,** c**) and (**b**, **d**) to enhance visibility of the weaker SST-front responses relative to LSCO, which would otherwise be indistinguishable on a common scale. The responses in (**a**, **c**) and (**b**, **d**) nevertheless remain statistically significant.

Our analyses have revealed that the midlatitude SST fronts substantially complement the impact of LSCO on both the PPSC and SSW frequency in the NH. To investigate the underlying dynamics, we analyze how the LSCO and SST fronts influence the vertical structure of the responses of zonal wind and temperature in our experiments (Fig. 3). The LSCO drives warming in most of the extratropical stratosphere and cooling in the polar uppermost stratosphere and mesosphere, in association with marked weakening of the stratospheric westerlies (Fig. 3a, c). The meridional temperature gradients are consistent with the vertical wind shear via thermal wind balance, in which the vertical shear of the zonal wind is proportional to the negative meridional temperature gradient. Specifically, anomalous positive poleward temperature gradient in the stratosphere aligns with anomalous negative wind shear (increasing easterly anomalies with height), whereas anomalous negative poleward thermal gradient in the uppermost stratosphere and mesosphere corresponds to anomalous positive wind shear (decreasing easterly anomalies with height). Adding SST fronts to the LSCO_NoFr configuration amplifies these patterns, intensifying the extratropical stratospheric warming, mesospheric cooling, and the associated weakening of the stratospheric westerlies (Fig. 3b, d). While these responses are weaker compared to those to LSCO, the SST fronts play an important supporting role, in addition to the primary impact of LSCO, in shifting the stratospheric climatic conditions of SSWs and PPSC towards the observed NH-conditions (Figs. 1, 2 and Supplementary Figs. 2, 3).

The stratospheric dynamics in these experiments are analyzed using the Transformed-Eulerian-Mean (TEM) framework, which provides a detailed representation of wave-mean flow interactions and their impact on the BDC and temperature-distribution (“Methods”). This approach decomposes the total wave forcing (TWF) of the mean flow into contributions from resolved wave forcing (RWF) and non-resolved wave forcing (NRWF), such as orographic and non-orographic gravity waves (“Methods”^28^,). In this framework, the Coriolis force, which maintains the meridional residual circulation, balances the TWF (obtained as RWF + NRWF). The overturning circulation is further decomposed into contributions from RWF and NRWF (Methods), enabling a more detailed understanding of their role. Negative values of TWF and associated components RWF and/or NRWF indicate deceleration of the mean westerly winds. According to the momentum budget (Eq. 1, “Methods”), this leads to enhanced poleward mass transport in the Coriolis term. In other words, the negative wave forcing strengthens the residual circulation, enhancing poleward flow in the low-to-mid latitudes and downward motion in the mid-to-high latitude stratosphere, which leads to anomalous adiabatic warming in the polar stratosphere. This polar stratosphere warming supports the occurrence of SSWs and suppresses PSC-formation. Conversely, positive values of TWF and the associated RWF and/or NRWF components accelerate the mean westerly winds and thereby weaken the residual circulation, leading to mid-to-high latitude cooling, which favors PSC-formation and reduces SSW frequency.

**Fig. 4:**
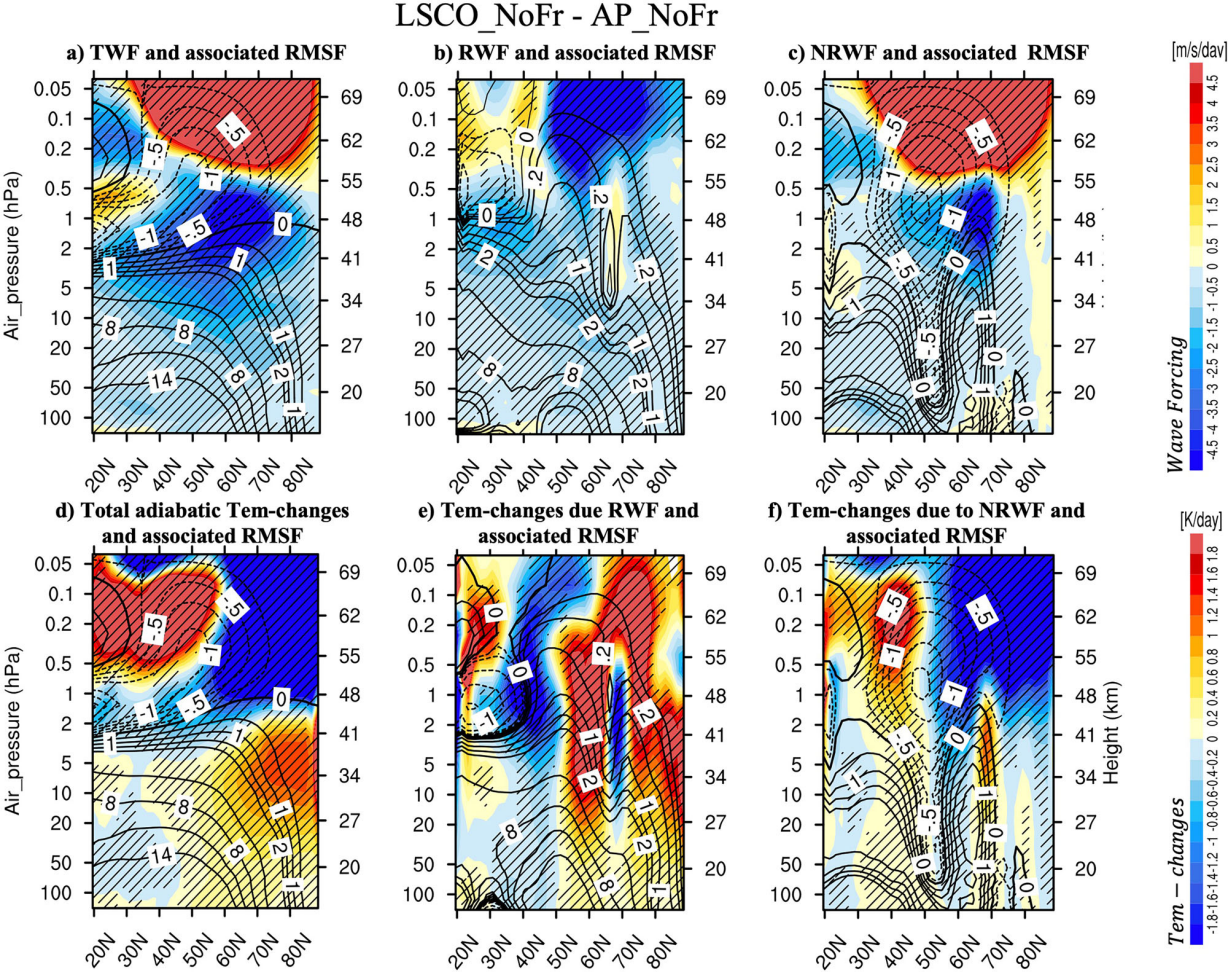
January–March (JFM)-mean dynamics of the stratospheric response to land–sea thermal contrast and orography (LSCO). The JFM climatological differences between LSCO_NoFr and AP_NoFr experiments in terms of wave forcing, residual circulation, and associated adiabatic Temperature (Temp) changes. Panels (**a**–**c**) illustrate, respectively, the total wave forcing (TWF) (shaded) and its associated residual mean stream function (RMSF) (in contours), the resolved wave forcing (RWF) and its associated RMSF, and non-resolved wave forcing (NRWF) (e.g., gravity wave drag) and its associated RMSF. Panels (**d**–**f**) follow the same structure as (**a**–**c**), respectively, but show the adiabatic Temperature changes (shaded) and the corresponding RMSF (in contours). Panel (**d**) depicts total adiabatic temperature changes associated with TWF, panel (**e**) focuses on adiabatic temperature changes driven by RWF, and panel (**f**) presents adiabatic temperature changes resulting from NRWF. Hatched areas represent regions where differences are statistically significant at the 95% confidence level, determined using a two-sided Student’s *t* test. Positive (negative) values of the residual circulation correspond to poleward (equatorward) circulation, and its upward (downward) branches coincide with blue (red) regions, indicating the resulting adiabatic cooling (warming).

Figure 4 highlights the effect of LSCO (as LSCO_NoFr - AP_NoFr) on TWF, RWF and NRWF, and their influence on the stratospheric residual circulation and adiabatic temperature changes. Incorporating LSCO into the aqua-planet configuration significantly enhances the net negative dynamical drag (TWF, Fig. 4a) across the extratropical stratosphere between 40^o^N and 70^o^N and below the 0.5hPa level. This results, according to the momentum budget (Eq. 1, “Methods”), in notable weakening of the westerlies. This enhanced negative drag is balanced mainly by the positive Coriolis force (Methods), and therefore strengthening of the BDC, particularly its downward branch. The enhanced BDC drives mid-to-high latitude adiabatic warming (Fig. 4d), explaining the stratospheric warming response in Fig. 3a. This adiabatic warming suppresses the conditions for PSC formation and strongly weakens the westerlies, but without replicating the observed NH SSW conditions (Supplementary Fig. 2, 3). The positive response in adiabatic warming $(-{\bar{w}}^{*}{\bar{\theta }}_{z})$ is balanced mainly by anomalous diabatic cooling${\bar{Q}}^{{dia}}$ (Eq. 2 in “Methods”).

In the mesosphere, a positive TWF above ~0.5 hPa (Fig. 4a) drives an anomalous negative residual circulation, characterized by anomalous enhanced upward motion and adiabatic cooling in the polar mesosphere (Fig. 4d). This cooling explains the mid-to-high latitudes mesospheric temperature reduction in Fig. 3a. The overall vertical structure of the zonal wind response remains consistent with the meridional gradient of adiabatic temperature changes, maintaining thermal wind-balance throughout the stratosphere and mesosphere (Figs. 3a and 4d).

In response to LSCO, the RWF emerges as the primary driver of the stratospheric BDC-response between 150 and about 2 hPa (Fig. 4), intensifying its subsidence branch and inducing stronger adiabatic warming in mid-to-high latitudes. However, NRWF also plays a significant role, especially near 60°N, where it locally enhances the BDC’s downward branch, contributing to additional stratospheric warming in regions where RWF weakens. Additionally, NRWF strengthens the BDC in the low-to-mid latitude stratosphere. In contrast, in the polar stratosphere and at about 50–60^o^N, NRWF induces a significant cooling response due mainly to its downward control effect originating from the mesosphere^52^.

The shifting of TWF from negative in the stratosphere to positive in the mesosphere is primarily driven by NRWF, consistent with the selective filtering of non-orographic gravity waves by the stratospheric background winds. In general, the stratospheric westerly winds filter out westerly gravity wave-drag, enabling easterly gravity wave-drag to propagate into the mesosphere, and vice versa^28,53^. In the LSCO-experiment, the RWF-induced weakening of the stratospheric westerlies suppresses easterly NRWF, permitting more westerly NRWF to reach the mesosphere, culminating in a net westerly forcing.

**Fig. 5:**
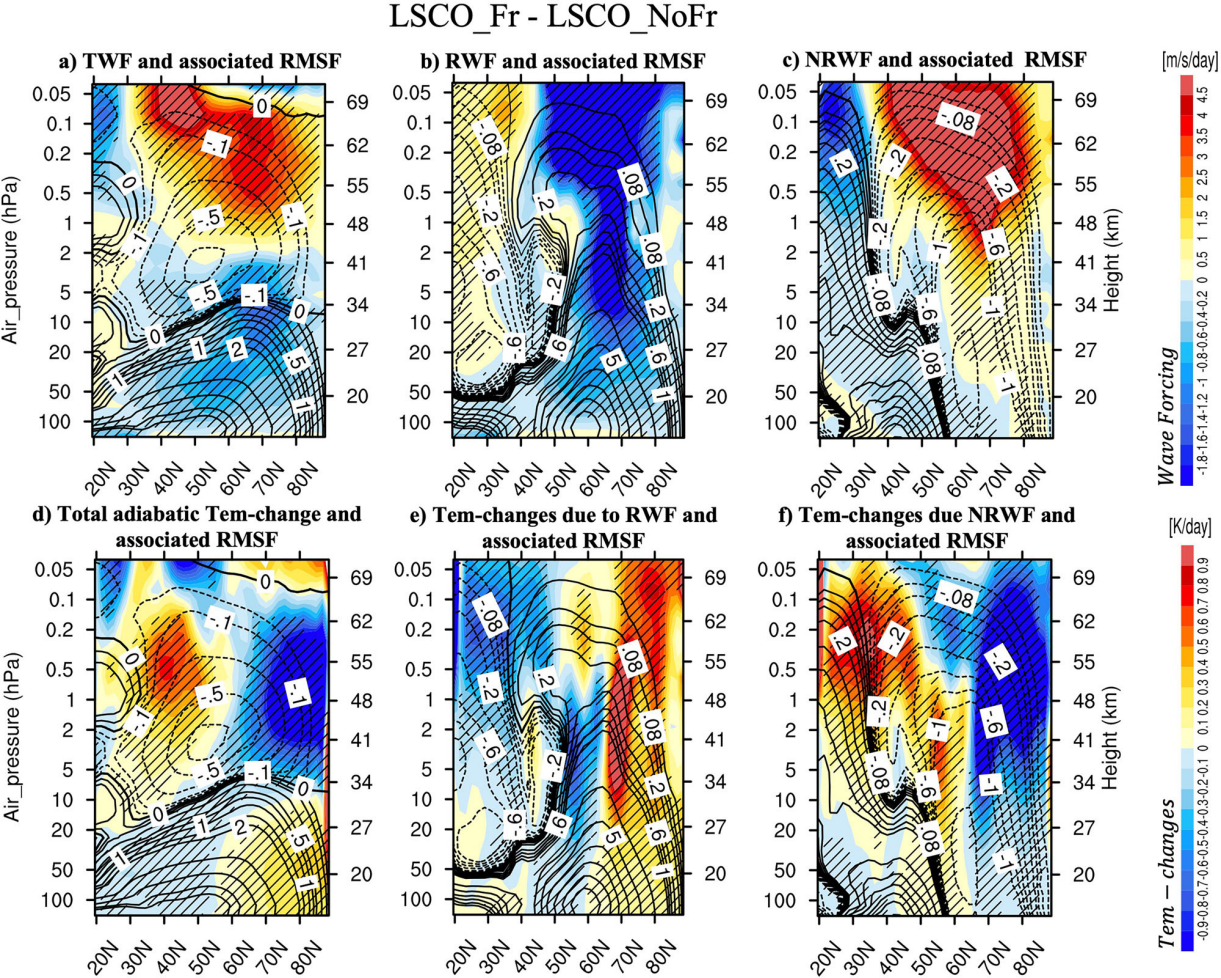
January–March (JFM)-mean dynamics of the stratospheric response to sea surface temperature (SST) fronts. The JFM climatological differences between LSCO_Fr and LSCO_NoFr experiments highlight the impact of SST fronts on wave forcing, residual circulation, and associated adiabatic Temperature (Temp) changes. Panels (**a**–**c**) depict, respectively, the total wave forcing (TWF) (shaded) and its residual mean stream function (RMSF) (contours), the resolved wave forcing (RWF) and its associated RMSF, and the non-resolved wave forcing (NRWF) (e.g., gravity wave drag) and its associated RMSF. Panels (**d**–**f**) show the adiabatic temperature changes (shaded) and the corresponding RMSF (contour). Specifically, panel (**d**) represents the total adiabatic temperature changes driven by TWF, panel (**e**) highlights adiabatic temperature changes due to RWF, and panel (**f**) focuses on those resulting from NRWF. Hatched regions denote statistically significant differences at the 95% confidence level, based on a two-sided Student’s *t* test. Positive (negative) values of the residual circulation correspond to poleward (equatorward) circulation, and its upward (downward) branches coincide with blue (red) regions, indicating the resulting adiabatic cooling (warming).

The midlatitude SST fronts exert significant influence on SSWs and PPSC (Figs. 1, 2), although their dynamical effects on the mean stratospheric warming and polar-vortex weakening are less pronounced than those driven by LSCO (Figs. 3, 4). The SST fronts notably amplify the easterly TWF (Fig. 5a) in the extratropical stratosphere to further weaken the westerlies. This enhanced easterly drag intensifies the BDC, particularly its high-latitudes downward branch, resulting in further adiabatic warming in the polar stratosphere (Fig. 5d). While this warming is less pronounced compared to the LSCO effect, it plays a critical supporting role by complementing the LSCO’s primary influence, bringing SSW frequency and PPSC conditions more in line with their observed NH conditions.

In the uppermost stratosphere and mesosphere (above ~ 2 hPa), the SST fronts induce positive TWF (Fig. 5a), generating anomalous negative residual circulation characterized by enhanced upward motion and adiabatic cooling in high latitudes. The vertical structure of the zonal wind response remains consistent with the meridional gradient of the adiabatic temperature-changes, maintaining thermal wind-balance throughout the mesosphere (Figs. 3b, 5d). RWF remains the primary driver of the BDC-strengthening and the associated adiabatic warming in the high-latitude stratosphere (Fig. 5). However, RWF, the associated BDC-strengthening and TWF in response to the SST front, are weaker compared to LSCO. Meanwhile, NRWF induces anomalous cooling in the high-latitude stratosphere, partially offsetting the RWF-induced warming and thus diminishing its overall effect. While less influential in strengthening the high-latitude branch of the BDC, NRWF plays an important role in reinforcing the BDC in the low-to-mid latitude stratosphere (20°N–50°N). Its contribution also enhances the downward branch of the BDC and supports additional warming in areas where the RWF influence weakens (50°–60°N).

As in the LSCO-response, the reversal of the SST front-induced TWF, shifting from negative in the stratosphere to positive in the mesosphere, is predominantly driven by NRWF. This reflects the selective filtering of non-orographic gravity waves by the anomalous background stratospheric winds. According to the momentum budget, this gravity wave-induced westerly forcing is associated with anomalous negative residual overturning circulation and cooling in the mesosphere (Fig. 5c, f). This response exerts a pronounced downward control, extending the anomalous negative residual circulation and its cooling effects from the mesosphere into the lowermost stratosphere.

As shown in Supplementary Fig 4 and Supplementary Table S1, the Pacific SST front plays a key role in the response of the NH SSWs and PPSCs to the midlatitude SST fronts. Extending the Eliassen-Palm (EP) flux analysis downward into the troposphere (Supplementary Fig. 8) shows that the enhanced stratospheric response of the RWF stems from stronger upward propagation of planetary waves from the lower atmospheric levels. This enhanced wave activity occurs only in experiments that include the Pacific SST fronts. The associated tropospheric circulation response is characterized by a significant lowering of geopotential height over the North Pacific and the associated strengthening of the Aleutian Low (Supplementary Fig 9a–d), a pattern widely recognized as a precursor to the weaker polar vortex states and SSW events^45–48^. Nishii et al. (2011) demonstrated that the opposite phase—positive geopotential height anomalies over the North Pacific (or blocking)—suppresses upward planetary-wave propagation, leading to a stronger stratospheric polar vortex. The tropospheric response in our Pacific front experiments is, by geostrophic balance, dynamically consistent with the previously shown intensification of the Pacific eddy-driven jet and storm track^35^, maintained by the Pacific SST-front-induced baroclinicity through turbulent heat fluxes. In contrast to the Pacific, the Atlantic SST front induces a tropospheric circulation resembling a positive NAO-like pattern (Supplementary Fig 9e, f). This configuration features a lowered geopotential height over the northern Euro-Atlantic and Scandinavian regions and lacks the northern Euro-Atlantic high geopotential height anomalies that typically precede the weak stratospheric polar vortex and SSW events (e.g., 47.). As a result, the Atlantic SST front suppresses upward planetary-wave propagation into the stratosphere and produces only a weak stratospheric response in the EP-flux (Supplementary Fig 8e, f), highlighting the dominant role of the Pacific sector in driving the hemispheric asymmetry.

## Discussion

This study highlights an important but previously underexplored role of midlatitude SST fronts in shaping the hemispheric asymmetry in SSWs and conditions conducive to PSCs. Through a suite of semi-idealized experiments, we isolate the contributions of SST fronts and LSCO to polar stratospheric dynamics. While LSCO strongly weakens and warms the stratospheric polar vortex, it alone is insufficient to account for the frequent SSW events observed in the NH. The addition of midlatitude SST fronts, particularly in the Pacific, substantially increases SSW frequency, demonstrating their critical complementary role in breaking down the stratospheric polar vortex and promoting Arctic-like stratospheric variability. The SST fronts also enhance the polar stratospheric warming via the residual circulation, suppressing the cold conditions required for Type I and Type II PSCs. In particular, the Pacific SST fronts significantly reduce the frequency and duration of Antarctic-like cold extremes in the NH, while the Atlantic SST fronts contribute much less.

**Fig. 6:**
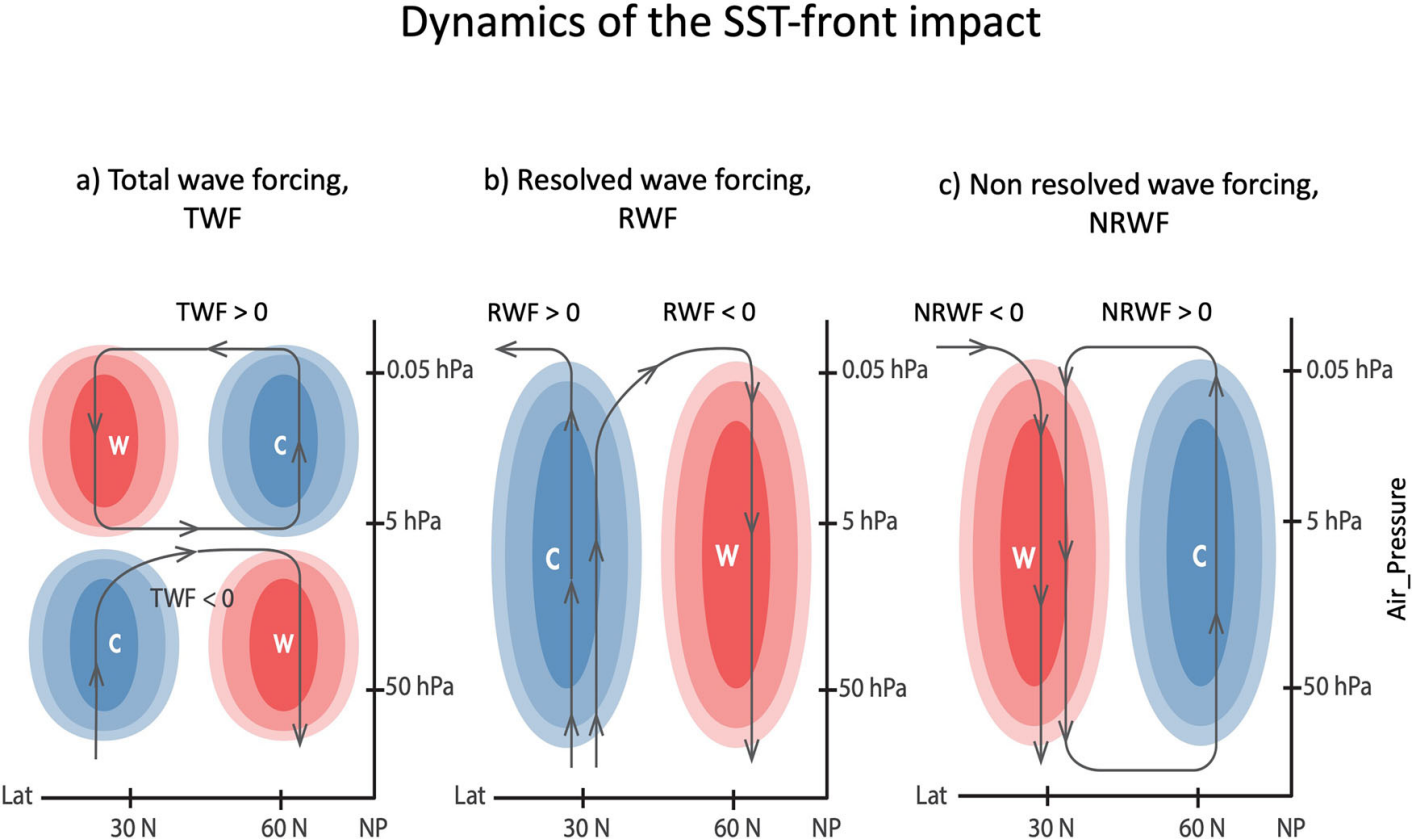
Schematic representation of the underlying dynamical response to sea surface temperature (SST) fronts. The panels illustrate changes in the adiabatic temperature (shading) and their associated residual circulation (arrows) driven by dynamical forcings in response to midlatitude SST fronts. Positive (warm (W), red) and negative (cold (C), blue) temperature changes indicate adiabatic warming and cooling, respectively, due to changes in residual circulation, resulting from the balance between dynamical forcings (total wave forcing (TWF), resolved wave forcing (RWF), and non-resolved wave forcing (NRWF)) and the Coriolis force (Methods). Negative dynamical forcing balances positive Coriolis forcing, strengthening the poleward residual circulation (or Brewer–Dobson Circulation (BDC)), while positive forcing induces an anomalous equatorward circulation. Panel (**a**) shows that in the low-to-mid stratosphere, negative TWF—mainly driven by RWF (panel **b**)—is balanced by enhanced poleward residual circulation, strengthening the Brewer-Dobson Circulation (BDC). This augments upward motion with adiabatic cooling in low latitudes and downward motion with adiabatic warming in mid-to-high latitudes, favoring Sudden Stratospheric Warmings (SSWs) and suppressing Polar Stratospheric Clouds (PSC) formation. In the uppermost stratosphere and mesosphere, by contrast, positive TWF (panel **c**)—primarily driven by NRWF—enhances equatorward residual circulation, with upward motion and adiabatic cooling in mid-to-high latitudes and downward motion and adiabatic warming in low latitudes. Panel (**b**) highlights that RWF due to planetary waves, which dominate in the stratosphere, primarily generates an overall negative forcing in the mid-to-high latitudes. This drives an anomalous poleward residual circulation, contributing significantly to the overall strengthening of the Brewer-Dobson Circulation (BDC). Panel (**c**) shows that NRWF, which surpasses RWF in the uppermost stratosphere and mesosphere, introduces substantial positive forcing in the mid-to-high latitude stratosphere and mesosphere. This forcing drives an anomalous equatorward residual circulation and adiabatic cooling in the polar mesosphere with strong downward control that extends into the lower stratosphere.

Mechanistically (Fig. 6), the SST fronts amplify the convergence of resolved planetary-wave forcing (RWF), strengthening the BDC and enhancing the adiabatic warming in its downward branch. In the upper stratosphere and mesosphere, they also modulate NRWF, consistent with the filtering of gravity wave drag. The NRWF acts to enhance the equatorward residual circulation and adiabatic cooling aloft, which exerts downward control on the stratosphere, cooling it and thus counteracting the warming effect of RWF in lower stratospheric levels. This modulation by NRWF is essential for shaping the net response of the vertical structure of the stratospheric warming. The combined effect of RWF and NRWF thus structures the vertical profile of changes in polar temperature and residual circulation, modulating the hemispheric contrasts in stratospheric extremes (Fig. 6).

While our analysis focuses on how SST fronts amplify RWF and its role in weakening the stratospheric polar vortex, it is crucial to emphasize that RWF itself is strongly modulated by the background wind structure in the stratosphere. According to the Charney–Drazin criterion^42^, vertically propagating planetary waves require westerly winds of moderate strength, if the background winds are excessively strong, the upward wave propagation is inhibited. Our results suggest that LSCO already weakens the background stratospheric westerlies, but not enough to allow for the observed frequency of SSWs. The addition of the SST fronts enhances stratospheric variability and provides the North Pacific tropospheric precursory conditions for upward wave propagation. With the already weakened background westerlies in the LSCO configuration, the effect of the SST fronts is further enhanced, according to the Charney-Drazin criterion, leading to additional weakening of the westerlies and an increase in the frequency of SSWs. This sequential weakening of the background wind helps explain why the SST fronts in the LSCO configuration have a much stronger dynamical impact than when they are in isolation, reinforcing the interaction between the convergence of upward-propagating planetary wave and the background westerly wind deceleration.

This study focuses on how permanent, stationary large-scale boundary condition differences between the NH and SH—specifically land–sea contrast, orography, and the presence of midlatitude SST fronts—control the marked asymmetry in SSW and PSC frequency between the two hemispheres. While interannual variability in SST gradients may modulate wave activity and stratospheric dynamics, such variability remains small relative to the persistent climatological boundary differences and cannot therefore account for the pronounced NH–SH contrast, since this contrast exists even in the presence of interannual ocean variability. Nonetheless, how interannual-to-interdecadal variability in SST fronts modules stratospheric dynamics remains an important question for future work. The SST fronts significantly enhance the interannual variability of conditions conducive to SSWs, even without two-way atmosphere-ocean coupling, which can further amplify this variability (Fig. 1). Both Atlantic and Pacific western boundary currents exhibit significant decadal variability^54,55^. However, the extent to which this variability influences the climatic conditions conducive to SSWs and PSC-formation remains unexplored. While large-scale oceanic variability, such as the Pacific-Decadal-Oscillation (PDO) and Atlantic-Multidecadal-Oscillation (AMO) has been shown to impact the stratospheric polar vortex and associated SSWs^11,50,51,56^, their potential impacts on PSCs and associated ozone depletion on multidecadal time-scales, which has been largely attributed to anthropogenic forcing^20^, also remain unexplored.

While insightful, the SST front experiments show some deviations from observed climatic conditions conducive to SSWs and PSCs, likely due to the absence of components like Arctic sea-ice, tropical SST-asymmetry, and two-way atmosphere-ocean interaction, in addition to potential model biases. The omissions of these factors was intentional to clearly isolate the impact of midlatitude SST fronts. By excluding other confounding factors, we could focus on the unique dynamical role of the SST fronts in forcing the atmospheric circulation without interference from complex climate processes. The stratospheric circulation response to the combined forcing of SST-fronts and Arctic sea-ice forcing (see Supplementary Fig. 10) does not differ significantly from the response to the SST-fronts alone. This indicates that, in our experiments, the SST-fronts exert a dominant influence on the stratospheric circulation compared to Arctic sea-ice. Despite some limitations, the semi-idealized framework has proven powerful, uncovering previously unexplored aspects of SST front dynamics and their impact. For example, it has provided key insights into the influence of SST fronts on storm-tracks, the eddy-driven jet, and atmospheric blocking, helping to address model biases in simulating these features^57^. Going forward, it is crucial to investigate how the misrepresentation of SST fronts in stratosphere-resolving models affects their ability to simulate stratospheric extreme events like SSWs and extreme cooling events conducive to PSCs, their variability and associated impact on surface climate, surface extremes and stratospheric ozone depletion. Such efforts could provide a pathway to refine model representations and improve the accuracy of climate projections, particularly under extreme conditions.

## Methods

### Model experiments

We used the MAECHAM5 atmospheric model developed at the Max Planck Institute for Meteorology^58,59^. The model extends into the mesosphere with 39 vertical levels reaching up to 0.01 hPa and runs at T63 horizontal resolution ( ~ 1.875°). It includes well-resolved stratosphere and comprehensive gravity wave parameterizations suitable for simulating Sudden Stratospheric Warmings (SSWs) and conditions favorable for Polar Stratospheric Clouds (PSCs) studies^56,59–61^.

Lower boundary forcing was prescribed using monthly mean climatological SSTs from 1950–2008, based on HadISST1 data^62^, which are interpolated to the model grid. In addition to the model experiments, we used NCEP reanalysis data^63^ to diagnose observed climatologies of SSWs and PSCs. The goal of this study is to use semi-idealized model experiments with standard resolution to understand which large-scale lower boundary structures—specifically land–sea thermal contrast, orography (LSCO), and oceanic SST fronts—are responsible for the hemispheric asymmetry in SSW and PSC frequency. This asymmetry is reproducible across both standard and higher model resolutions, provided the stratosphere is well-resolved and appropriate gravity wave parameterizations are included. To support the use of standard T63 resolution, Supplementary Fig. 5–7 compares SSW and PPSC statistics based on two model configurations of coupled (MPI-ESM) for the standard resolution (T63 atmosphere, ~ 1.9°, 100 km ocean) and higher-resolution setup (T127 atmosphere, ~ 1.0°, 40 km ocean). As expected, the NH–SH asymmetry remains robust across resolutions, demonstrating that the key asymmetry is insensitive to the model horizontal resolution.

To isolate the contribution of WBC-driven SST fronts, a series of Atmospheric General Circulation Model (AGCM) experiments were performed with systematically modified SST gradients. First, a Non-Front (NF) SST profile was created by taking zonally averaged observed SSTs and eliminating the mid-latitude frontal gradient in both hemispheres by imposing a linear poleward decrease of the SST with constant gradient north (south) of 33.5°N (33.5°S). This created a constant meridional SST gradient from the tropical flanks of the fronts (33.5°N/S) to the poles, assuming a linear poleward temperature decrease (see Supplementary Fig. 1). Sea ice was removed in all experiments to avoid introducing artificial SST gradients at the ice edge of the non-front configurations, which would otherwise interfere with the intended removal of oceanic SST fronts. This artificial gradient arises from the way the SST fronts are removed—using a zonally symmetric, linearly decreasing SST profile—which creates an abrupt discontinuity when intersected by fixed sea-ice boundaries. This sea-ice-free experimental setup follows earlier semi-idealized modeling approaches^35–38,41^, here adapted for a systematic hemispheric comparison framework.

We then designed a series of semi-idealized experiments to separately assess the roles of LSCO and midlatitude SST fronts in modulating the BDC, SSWs, and PSC-related temperatures (see Supplementary Table S1). The AP_NoFr configuration represents a zonally symmetric aquaplanet baseline experiment mimicking SH-like conditions without SST fronts. The AP_Fr experiment adds zonally averaged Atlantic SST fronts to this setup, mimicking SH-like conditions with SST fronts. To assess the impact of terrestrial features, the LSCO_NoFr experiment adds realistic LSCO to the aquaplanet configuration without SST fronts (AP_NoFr), simulating NH-like conditions without SST fronts. The LSCO_Fr configuration builds on this by incorporating realistic midlatitude SST fronts in both ocean basins, representing NH-like conditions with all key structural features (Supplementary Fig. 1).

In interpreting the combined impact of LSCO and SST fronts, we carefully designed our experiments to ensure both physical realism and mathematical consistency. To isolate the specific effect of LSCO, we compare AP_NoFr and LSCO_NoFr, which differ only by the presence of land and orography. The effect of SST fronts in the NH must be assessed in the presence of LSCO, since these features are an inseparable part of the real-world NH environment. The AP configuration, which lacks LSCO, provides a zonally symmetric baseline more representative of SH-like conditions. Consequently, the difference LSCO_Fr – LSCO_NoFr appropriately captures the SST-front effect in the NH context, while AP_Fr – AP_NoFr reflects SST-front effects under SH-like conditions. Furthermore, the combined effect of LSCO and SST fronts (LSCO_Fr – AP_NoFr) satisfies mathematical additivity, as it can be decomposed into the sum of LSCO_NoFr – AP_NoFr and LSCO_Fr – LSCO_NoFr. Each experiment is integrated continuously for approximately 62 years in repeating the annual cycle of monthly climatological SSTs from HadISST1. To examine basin-specific contributions, the LSCO with a realistic Pacific SST-front (LSCO_PaFr) configuration incorporates a realistic Pacific SST front to isolate its specific role in SSWs and PSCs, while the LSCO with a realistic Atlantic SST-front (LSCO_AtFr) focuses on its corresponding role within the same framework.

### Calculation of Potential formation of Polar Stratospheric Clouds (PPSC)

The Potential for Polar Stratospheric Cloud (PPSC) represents the percentage of the hemispheric area north of 40°N meeting the temperature thresholds for PSC formation^21^. It is calculated daily for both Type I (PPSC-I) and Type II (PPSC-II) PSCs as follows: surface areas of grid cells with temperatures below the 195 K and 188 K thresholds representing PPSC-I (nitric acid trihydrate, NAT) and PPSC-II (water ice), respectively, are summed within the region north of 40°N. This area is normalized by the total NH area, yielding the PPSC as a percentage of the hemispheric area. The resulting daily PPSC values represent the extent of conditions conducive to PSC formation within the defined hemisphere region.

### Identifying the SSW events

SSW events are identified following^3^, as the day when the zonal-mean zonal wind at 60°N and 10 hPa is reversed to easterly during the boreal winter (November– March). Each SSW event in the same winter must be separated by consecutive westerlies of at least 20 days. Although the inversion of the meridional temperature gradient criterion is not explicitly included, it is implicitly accounted for under thermal wind balance. Only zonal wind reversals that return to westerly are counted as SSWs. Persistent springtime reversals that do not return to westerly are classified as final warmings and are excluded from the SSW count.

### Dynamical diagnostics

To understand the dynamics of the stratospheric adjustment to different boundary conditions, we used the momentum (Eq. 1) and heat (Eq. 2) budgets in the transformed Eulerian mean (TEM) formalism. This formalism has been widely used to understand the extratropical wave-mean flow interaction. We first used the primitive equations described in ref. ^28^ and kept only the dominant terms in the zonally averaged zonal wind $(\bar{u})$ and potential temperature ($\bar{\theta }$) budgets that can be approximated to:

1 $${\bar{u}}_{t}=\frac{1}{({{{\rm{\rho }}}}_{0}\cos \phi )}\nabla \cdot {{\bf{F}}}+\hat{f}{\bar{v}}^{*}+\bar{X}\approx \frac{1}{({{{\rm{\rho }}}}_{0}\cos \phi )}\nabla \cdot {{\bf{F}}}+f{\bar{v}}^{*}+\bar{X}$$

2 $${\bar{\theta }}_{t}=-{\bar{w}}^{*}{\bar{\theta }}_{z}+{\bar{Q}}^{{dia}}$$

where $\nabla \cdot {{\bf{F}}}$ is the EP-flux divergence:

3 $$\nabla \cdot {{\bf{F}}}=\frac{1}{(a\cos \phi )}\frac{\partial \left({F}^{\phi }\cos \phi \right)}{\partial \phi }+\frac{\partial {F}^{z}}{\partial z}$$

4 $${F}^{\phi }={\rho }_{0}a\cos \phi \left({\bar{u}}_{z}\frac{\bar{v{\prime} \theta }{\prime} }{{\bar{\theta }}_{z}}-\bar{{u}^{{\prime} }{v}^{{\prime} }}\right)$$

and

5 $${F}^{z}={\rho }_{0}a\cos \phi \left(\frac{{\hat{f}}\bar{{v}^{{\prime} }{\theta }^{{\prime} }}}{{\bar{\theta }}_{z}}-\bar{u^{\prime} w^{\prime} }\right)$$

representing respectively the meridional and vertical components of the EP-flux vectors ${{\bf{F}}}$.

$${\bar{v}}^{*}=\bar{v}-{{\rho }_{0}}^{-1}{\left({\rho }_{0}\frac{\bar{{v}^{{\prime} }{\theta }^{{\prime} }}}{{\bar{\theta }}_{z}}\right)}_{z}6$$

$${\bar{w}}^{*}=\bar{w}+{{\left(a\cos \phi \right)}^{-1}\left(\cos \phi \frac{\bar{{v}^{{\prime} }{\theta }^{{\prime} }}}{{\bar{\theta }}_{z}}\right)}_{\phi }7$$

are the meridional and vertical residual velocities, respectively. In these equations, $\bar{v}$ and $\bar{w}$ are the zonally averaged meridional and vertical velocities. ()’ signifies a deviation from the zonal mean. ${()}_{t}$, ${()}_{z}$ and ${()}_{\phi }$ are the derivatives with respect to time t, vertical coordinate z, and latitude $\phi$_._ $f=2\Omega \sin \phi$ is the Coriolis parameter, $\Omega=7.29\times {10}^{-5}{{{\rm{s}}}}^{-1}$ is the rotation rate of Earth, $a$ is Earth’s radius; ${\rho }_{0}$ is air density. $\bar{X}$ represents the zonally averaged non-resolved wave forcing (NRWF), such as orographic and non-orographic gravity waves, $f{\bar{v}}^{*}$ is the residual Coriolis forcing and $\hat{f}=f-{\left(a\cos \phi \right)}^{-1}{\left(\bar{u}\cos \phi \right)}_{\phi}.$ In general, the net effects of the resolved wave forcing (RWF), represented as $\frac{1}{{{{\rm{\rho }}}}_{0}\cos \phi }\nabla \cdot {{\bf{F}}}$, and NRWF, represented $\bar{X}$ in ref. ^1^, are balanced by the residual Coriolis forcing $(f{\bar{v}}^{*})$. Specifically, the EP-flux convergence (i.e., negative or easterly RWF) or/and negative (or easterly) NRWF tend to be balanced by positive residual Coriolis forcing $(f{\bar{v}}^{*} > 0)$, which is manifested as enhanced meridional residual circulation and vice versa.

In Eq. 2, the first term on the right-hand side represents the adiabatic temperature change associated with residual vertical motion and the second term the total effect of the diabatic heating (including short and long-wave radiative heating/cooling and latent heat release). $\bar{X}$ and ${\bar{Q}}^{{dia}}$ are in general computed as residuum in the primitive TEM-equations^28^. The residual term ($\bar{X}$) represents the net momentum forcing not captured by resolved planetary wave activity, which is commonly interpreted as the contributions from subgrid-scale processes, such as gravity wave drag in the stratosphere^53,64^ and friction near the surface. In the lower and middle stratosphere (below ~ 30 hPa), orographic gravity wave drag typically dominates the overall gravity wave forcing, while in the upper stratosphere and mesosphere, non-orographic gravity waves become the primary contributors. Although we did not explicitly output the individual gravity wave drag components in our simulations, the vertical structure of $\bar{X}$ is consistent with known gravity wave filtering behavior^28,53^: enhanced planetary wave activity alters the background wind field, which in turn modulates gravity wave propagation and dissipation.

The model’s orographic gravity wave drag is parameterized by using the scheme of McFarlane^65^, which depends on near-surface winds and topographic slope. The non-orographic gravity wave drag is based on the spectral formulation of Hines^66,67^, which launches a broad spectrum of gravity waves from the troposphere and then estimates the momentum deposition based on wave filtering and saturation processes. In general, the sensitivity of the stratosphere in an AGCM to SST forcing can be influenced by the gravity wave parameterization scheme incorporated, and can therefore be model-dependent. However, as shown in refs. ^56,68^, including middle atmosphere and associated gravity wave parameterizations, are essential for realistically capturing stratospheric variability and its response to lower-boundary SST-conditions.

For diagnosing the BDC or residual meridional overturning circulation, we used the total residual mass stream function defined as:

8 $${\varPsi }^{{tot}}=-\frac{\cos \phi }{g}{\int }_{p}^{0}{\bar{v}}^{*}d{p}^{{\prime} }$$

Positive ${\Psi }^{{tot}}$ in the NH represents poleward meridional overturning circulation with upward (downward) motion and therefore adiabatic cooling (warming) at lower (higher) latitudes, and vice versa for negative ${\Psi }^{{tot}}$.

For diagnosing the contribution of the RWF ($\nabla \cdot {{\bf{F}}}$) to the residual meridional overturning circulation, we computed the downward control^52,53,61^ residual mass stream function that can be approximated to:

9 $${\Psi }^{{rw}}=\frac{\cos \phi }{g}{\int }_{p}^{0}{({\bar{v}}{*})}^{{{\rm{rw}}}}{dp}^{\prime}=\frac{\cos \phi }{g}{\int }_{p}^{0}\frac{({{\rho }_{0}\cos \phi })^{-1}\nabla \cdot {{\bf{F}}}} {\hat{f}} {dp}^{\prime}$$

With ${({\bar{v}}^{*})}^{{{\rm{rw}}}}$ = $\frac{({{\rho }_{0}\cos \phi })^{-1}\nabla \;\cdot\; {{\bf{F}}}}{\hat{f}}$ is the residual meridional velocity obtained from Eq^1^. in quasigeostrophic and stationary case by considering only the effect of wave forcing neglecting the non-resolved forcing $\bar{X}$. In this work, we used the full form of ${({\bar{v}}^{*})}^{{{\rm{rw}}}}$ obtained from the primitive TEM-equation(*28*) by assuming stationarity and neglecting both $\bar{X}$ and the vertical advection term.

The residual circulation due to NRWF $\left(\bar{X}\right)$ can be evaluated as the difference between ${\varPsi }^{{tot}}$ and ${\varPsi }^{{rw}}$:

10 $${\varPsi }^{{nr}}={\varPsi }^{{tot}}-{\varPsi }^{{rw}}$$

To understand the origin of the stratospheric adiabatic temperature response, we first computed the residual vertical motion associated respectively with RWF and NRWF according to:

11 $${({\bar{w}}^{*})}^{{{\rm{rw}}}}=\frac{{gH}}{({pa}f\cos \phi )}{\left({\varPsi }^{{rw}}\right)}_{\phi }$$

12 $${({\bar{w}}^{*})}^{{{\rm{nr}}}}=\frac{{gH}}{({pa}f\cos \phi )}{\left({\varPsi }^{{nr}}\right)}_{\phi }$$

Afterwards, we computed the associated dynamical potential temperature change using Eq^2^. as:

13 $${\left({\bar{\theta }}_{t}\right)}^{{{\rm{rw}}}}=-{({\bar{w}}^{*})}^{{{\rm{rw}}}}{\bar{\theta }}_{z}$$

14 $${\left({\bar{\theta }}_{t}\right)}^{{{\rm{nr}}}}=-{({\bar{w}}^{*})}^{{{\rm{nr}}}}{\bar{\theta }}_{z}$$

## Supplementary information

Supplementary Information

Transparent Peer Review file

## Data Availability

The raw climate model output generated in this study is available under restricted access due to both the very large size of the datasets (several terabytes) and because the simulations are currently being used in ongoing research projects. Access to the data required to reproduce the results presented in this study can be obtained by contacting the corresponding author at nour.omrani@uib.no. Data will be shared with researchers for the purpose of reproducing the results presented in this study via institutional data-transfer services. Requests are typically processed within two weeks. This study also uses NCEP reanalysis data, which are publicly available from the National Centers for Environmental Prediction (NCEP) and can be accessed at: https://psl.noaa.gov/data/gridded/data.ncep.reanalysis.html.

## Code availability

The code for sudden stratospheric warming (SSW) detection is publicly available at https://github.com/sandrolubis/Sudden-Stratospheric-Warmings and at Zenodo (10.5281/zenodo.19021451). The code used for dynamical analysis (e.g., transformed Eulerian mean diagnostics and residual circulation) and polar stratospheric cloud (PSC) diagnostics is publicly available at Zenodo (10.5281/zenodo.18248247), along with a subset of data for testing purposes.
