# Supplementary material for: Oceanic fronts shape hemispheric contrasts in polar stratospheric extremes
Source: Nat Commun. 2026 Apr 20;17:5462. doi: 10.1038/s41467-026-71998-5 (PMC13284378; doi:10.1038/s41467-026-71998-5)
Supplement: Supplementary file 1 — Supplementary Information [file 41467_2026_71998_MOESM1_ESM.pdf]

Oceanic Fronts Shape Hemispheric Contrasts in Polar Stratospheric Extremes (Supplementary Information)

Nour-Eddine Omrani[1], Fumiaki Ogawa[2], Hisashi Nakamura[3,4], Sandro W. Lubis[5], Noel S. Keenlyside[1,8] and Luca Famooss Paolini[6,7]

[1] Geophysical Institute, University of Bergen and Bjerknes Centre for Climate Research, Bergen, Norway.

[2] Faculty of Bioresources, Mie University, Tsu, Japan.

[3] Research Center for Advanced Science and Technology, University of Tokyo, Tokyo, Japan

[4] Japan Agency for Marine-Earth Science and Technology, Yokohama, Japan

[5] Pacific Northwest National Laboratory, Richland, WA, USA

[6] Department of Physics and Astronomy "Augusto Righi", University of Bologna, Bologna, Italy

[7] CMCC Foundation - Euro-Mediterranean Center on Climate Change, Bologna, Italy

[8] Nansen Environmental and Remote Sensing Center, Bergen, Norway

Correspondence to: Nour-Eddine Omrani (noureddine.omrani@uib.no)

This Supplementary Information provides additional details on the experiments and supporting figures that underpin the main results of the study.

Experiment Name	Land-Sea Thermal Contrast & Orography	SST Front	SSW-frequency per decade
**AP_NoFr**	No	No	0
**AP_Fr**	No	Yes (Zonally averaged)	0
**LSCO_NoFr**	Yes	No	1
**LSCO_Fr**	Yes	Yes	9
**LSCO_AtFr**	Yes	Yes (Atlantic Only)	1
**LSCO_PaFr**	Yes	Yes (Pacific Only)	10

Table S1: Overview of model experiments and simulated Sudden Stratospheric Warming (SSW) frequency: This table summarizes the experimental configurations used in this study, including the presence or absence of land–sea thermal contrast and orography (LSCO) and midlatitude sea surface temperature (SST) fronts. The corresponding SSW frequency is reported as the number of events per decade (see Methods).

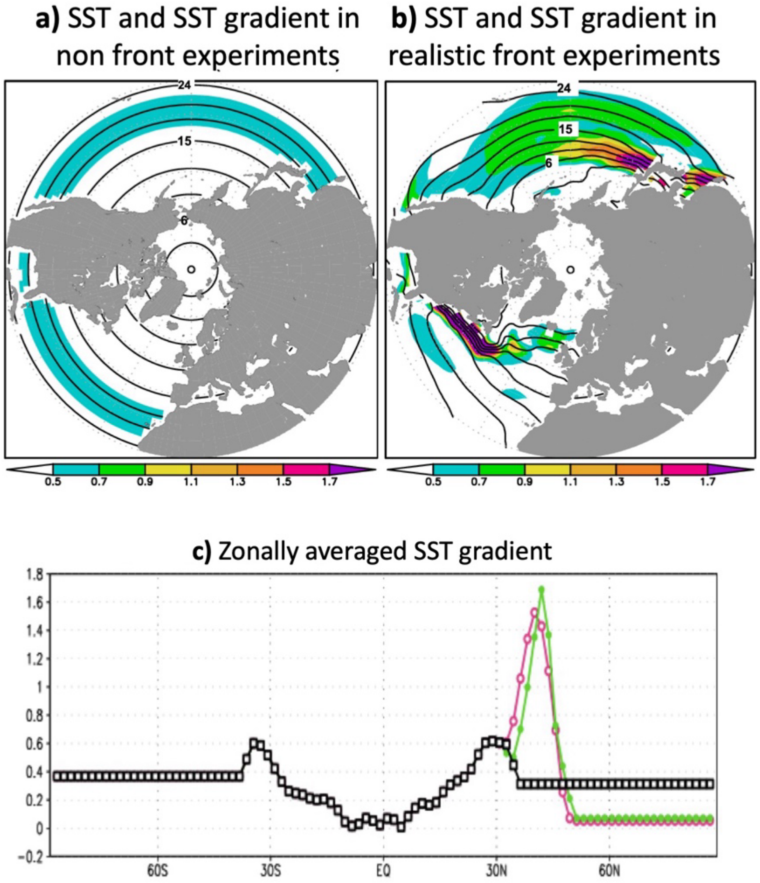

Supplementary Figure 1: Sea surface temperature (SST) forcing (Adapted from (*1*), *Scientific Reports*, licensed under CC BY 4.0, (*1*)): a) and b) represent the January SST (contours) and its meridional gradients (shaded) plotted for a) non-front (NF) experiment and b) both (Atlantic and Pacific) climatological SST fronts in the LSCO_Fr experiment (Methods). c) represents the latitudinal profiles of zonally averaged SST gradient used in the NF-experiments (black) as well as the frontal SST gradients in the North Atlantic (green) and North Pacific (pink) associated with the Gulf Stream and Kuroshio, respectively.

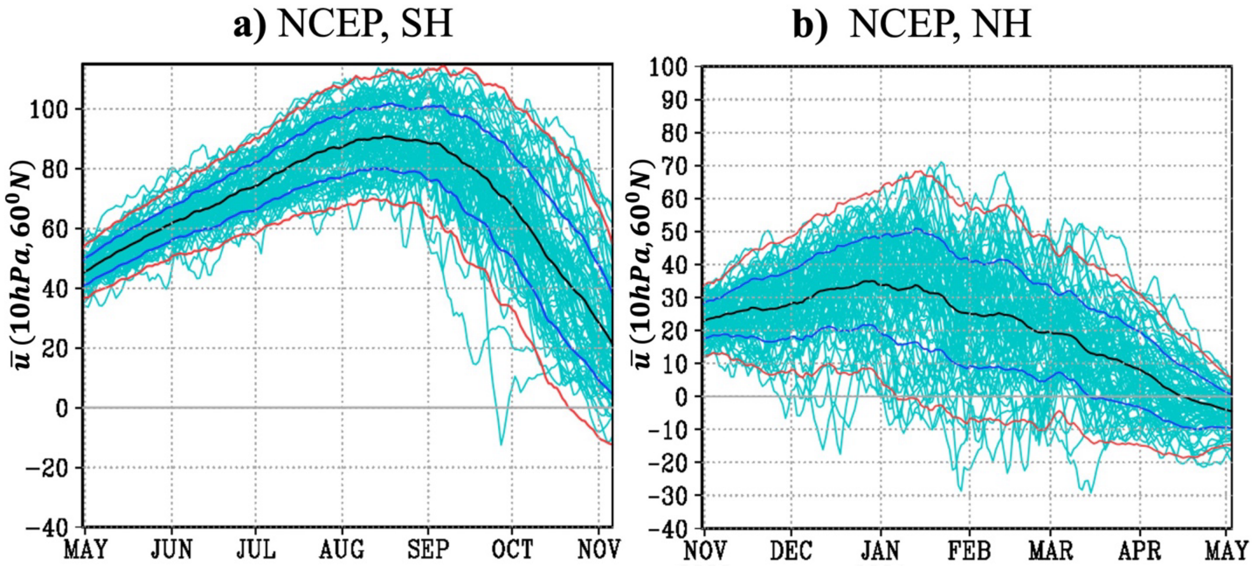

Supplementary Figure 2: Distinct seasonal evolution of the stratospheric zonally averaged zonal wind between the two hemispheres (National Centers for Environmental Prediction (NCEP) reanalysis): Like Fig. 1, these panels depict the seasonal evolution on daily timescales of the zonally averaged westerly wind ($\bar{u}$) at 10 hPa in a) the Southern Hemisphere (SH) at 60°S and b) the Northern Hemisphere (NH) at 60°N. The black lines indicate the climatological mean seasonal cycles, while the blue and red lines represent the ±1 and ±2 standard deviation margins, respectively. The zero-wind threshold is highlighted, to indicate the transition from westerly to easterly winds, corresponding to the occurrence of major Sudden Stratospheric Warmings **(**SSWs).

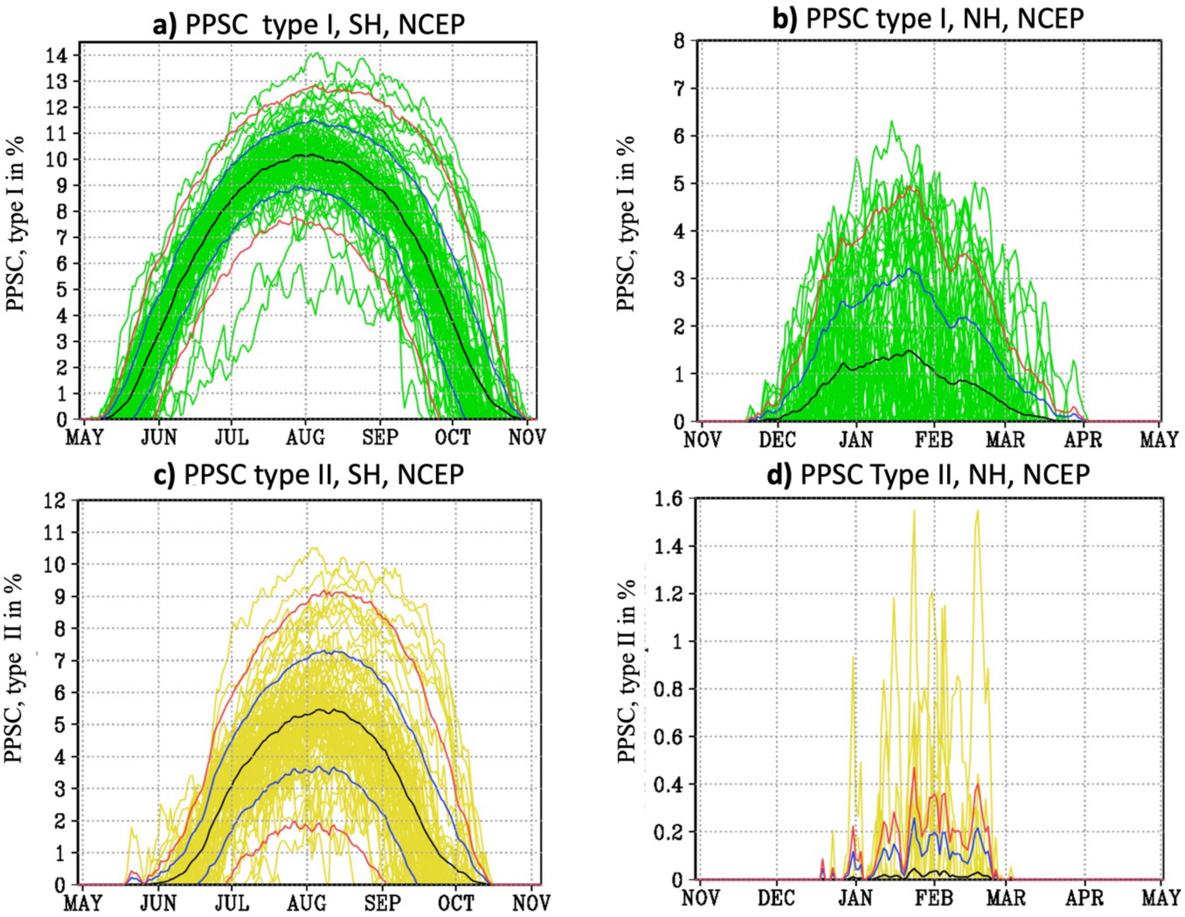

Supplementary Figure 3: Seasonal evolution of the potential formation of polar stratospheric clouds (PPSC) types I and II (National Centers for Environmental Prediction (NCEP) reanalysis): Like Fig. 2, these panels illustrate the seasonal evolution of the PPSC (Methods) for the Northern Hemisphere (NH) and Southern Hemisphere (SH) using the NCEP reanalysis. Panels a) and c) depict PPSC types I and II, respectively, for SH. Panels b) and d) depict the same as in a) and c), respectively, but for the NH. PPSC I (PPSC II) are defined as the percentage of the hemispheric area where temperature falls below 195 (188) K. The black lines indicate the mean seasonal cycles, while the blue and red lines represent the ±1 and ±2 standard deviation margins, respectively.

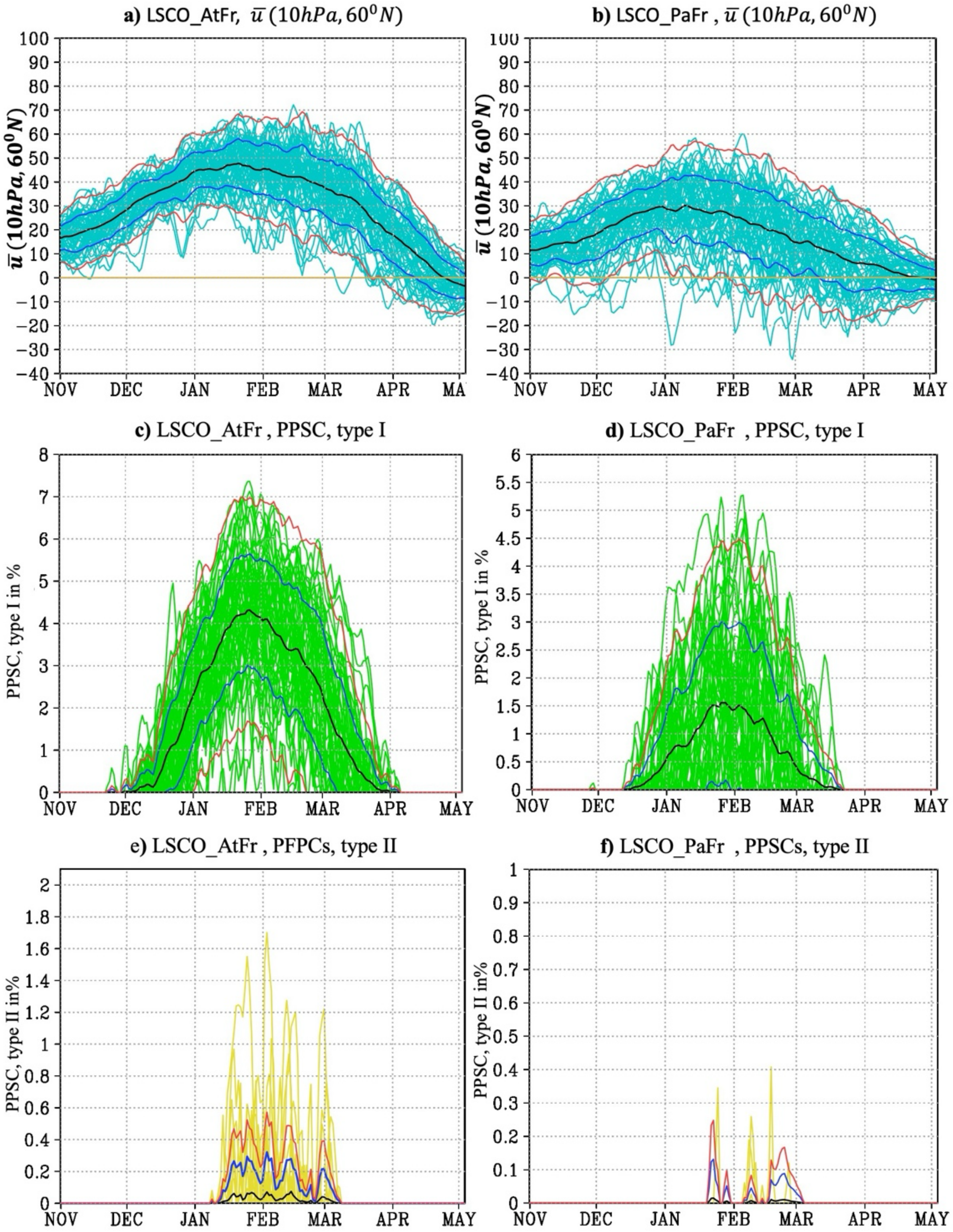

Supplementary Figure 4: The November (NOV) to April (APR) evolution of the stratospheric zonally averaged zonal wind and Potential for Polar Stratospheric Clouds (PPSC) (Atlantic and Pacific experiments): Like Fig. 1, a) and b) depict the NOV-APR evolutions of the zonally averaged westerly wind ($\bar{u}$) at 10 hPa and 60°N for the Atlantic SST-front (LSCO_AtFr) and Pacific sea surface temperature (SST)-front (LSCO_PaFr) experiments, respectively. c) and d) represent PPSC type I for the LSCO_AtFr and LSCO_PaFr experiments, respectively. e) and f) represent PPSC type II for LSCO_AtFr and LSCO_PaFr experiments, respectively. The black lines indicate the mean seasonal cycles, while the blue and red lines represent the ±1 and ±2 standard deviation margins, respectively. In a) and b) the zero-wind threshold is highlighted to mark the transition from westerly to easterly flow associated with Sudden Stratospheric Warmings (SSWs).

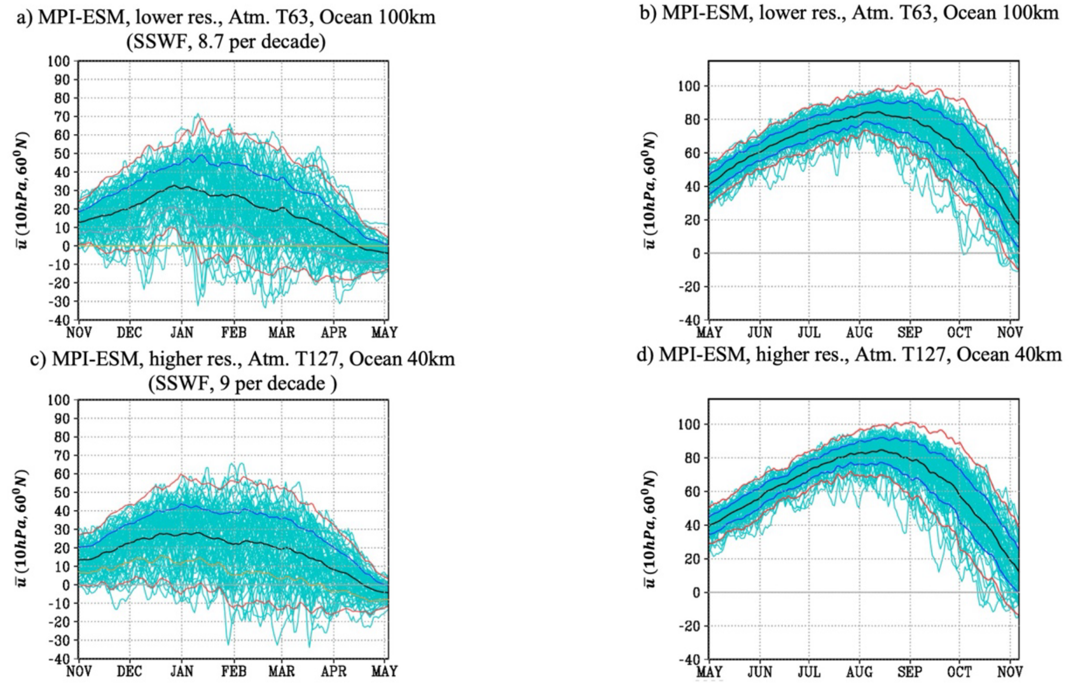

Supplementary Figure 5: Seasonal evolution of the stratospheric zonally averaged zonal wind (Max Planck Institute Earth System Model MPI-ESM coupled simulations): Like Supplementary Figure 2, these panels show the extended wintertime evolution of the zonally averaged westerly wind ($\overline{u}$) at 10 hPa (a, c) in the Northern Hemisphere (NH) at 60°N and (b, d) in the Southern Hemisphere (SH) at 60°S. Panels (a, b) correspond to the lower-resolution configuration (T63 atmosphere, ~1.9°, 100 km ocean), and (c, d) to the higher-resolution configuration (T127 atmosphere, ~1.0°, 40 km ocean). The black lines indicate the mean seasonal cycles, while the blue and red lines represent the ±1 and ±2 standard deviation margins, respectively. The zero-wind threshold is highlighted to mark the transition from westerly to easterly flow associated with Sudden Stratospheric Warmings (SSWs). The mean SSW frequency is 8.7 decade^{-1} in the lower-resolution model and 9 decade^{-1} in the higher-resolution model.

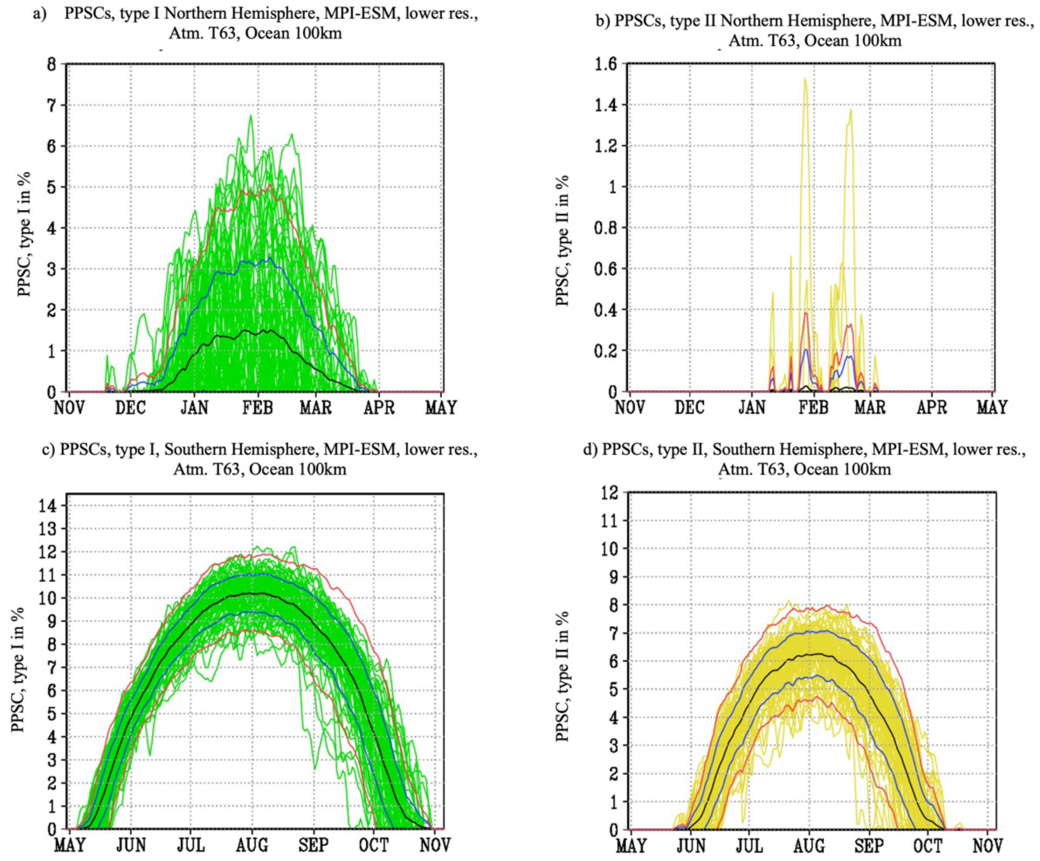

Supplementary Figure 6: Seasonal evolution of the Potential for Polar Stratospheric Clouds (PPSC) (MPI-ESM coupled simulations): Like Supplementary Figure 3 and Fig. 2, these panels illustrate the extended wintertime evolution of the PPSC (see Methods) for the Northern Hemisphere (NH) and Southern Hemisphere (SH) in the MPI-ESM lower-resolution configuration (T63 atmosphere, ~1.9°, 100 km ocean). Panels a) and b) show PPSC type I and type II, respectively, for the NH, while panels c) and d) depict PPSC type I and type II, respectively, for the SH. PPSC I and II correspond to the percentage of hemispheric areas where temperature falls below 195 K and 188 K, respectively. The black lines indicate the mean seasonal cycles, and the blue and red lines represent the ±1 σ and ±2 σ variability, respectively. The simulation reproduces the strong hemispheric asymmetry in PSC occurrence, with frequent PSC conditions in the SH and rare Polar Stratospheric Cloud (PSC) formation in the NH, consistent with the simulated Sudden Stratospheric Warming (SSW) climatology.

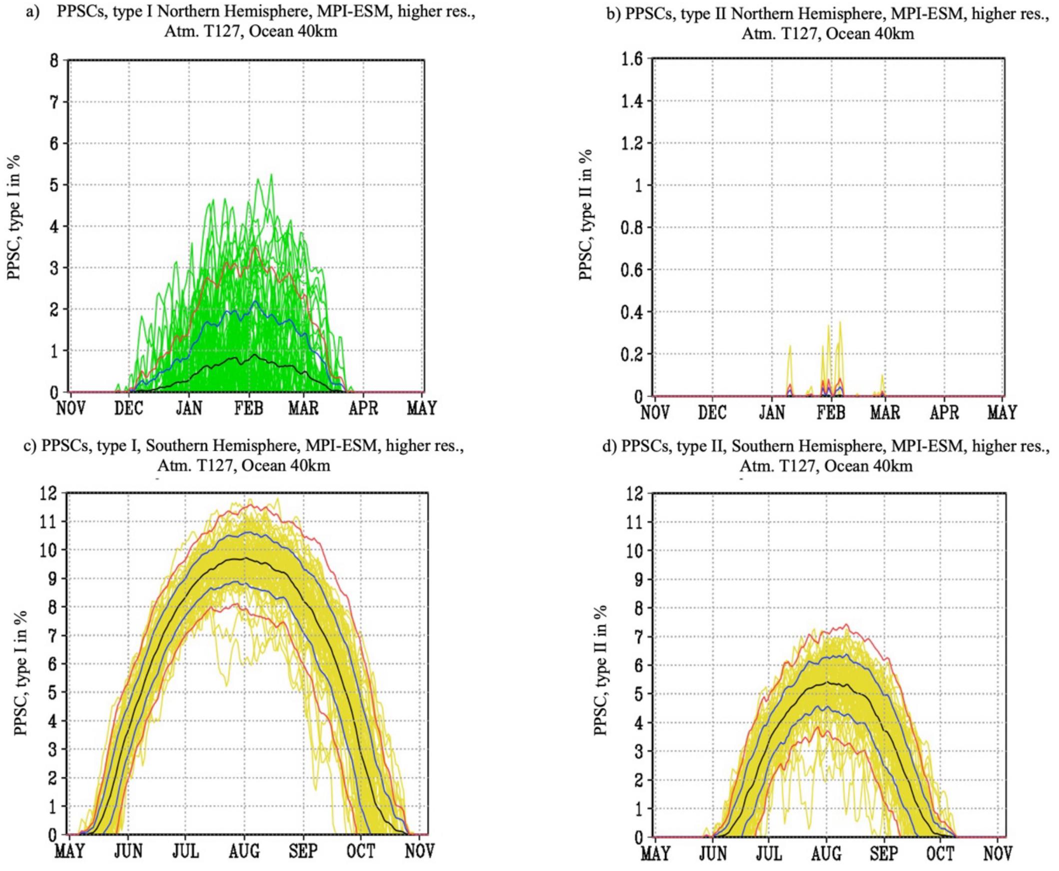

Supplementary Figure 7: November to early May evolution of Potential for Polar Stratospheric Clouds (PPSC) types I and II (MPI-ESM, higher resolution): Like Supplementary Figure 5, these panels illustrate the seasonal evolution of the PPSC (see Methods) for the Northern Hemisphere (NH) and Southern Hemisphere (SH) in the MPI-ESM higher-resolution configuration (T127 atmosphere, ~1.0°, 40 km ocean). Panels a) and b) show PPSC type I and type II, respectively, for the NH, and panels c) and d) show PPSC type I and type II, respectively, for the SH. PPSC I and II correspond to the percentage of hemispheric areas where stratospheric temperature falls below 195 K and 188 K, respectively. The black lines indicate the mean seasonal cycles, while the blue and red lines represent the ±1 σ and ±2 σ margins, respectively. The higher-resolution configuration exhibits a slightly reduced overall PPSC occurrence—particularly in the NH—consistent with the increased Sudden Stratospheric Warmings (SSWs) frequency compared to the lower-resolution simulation.

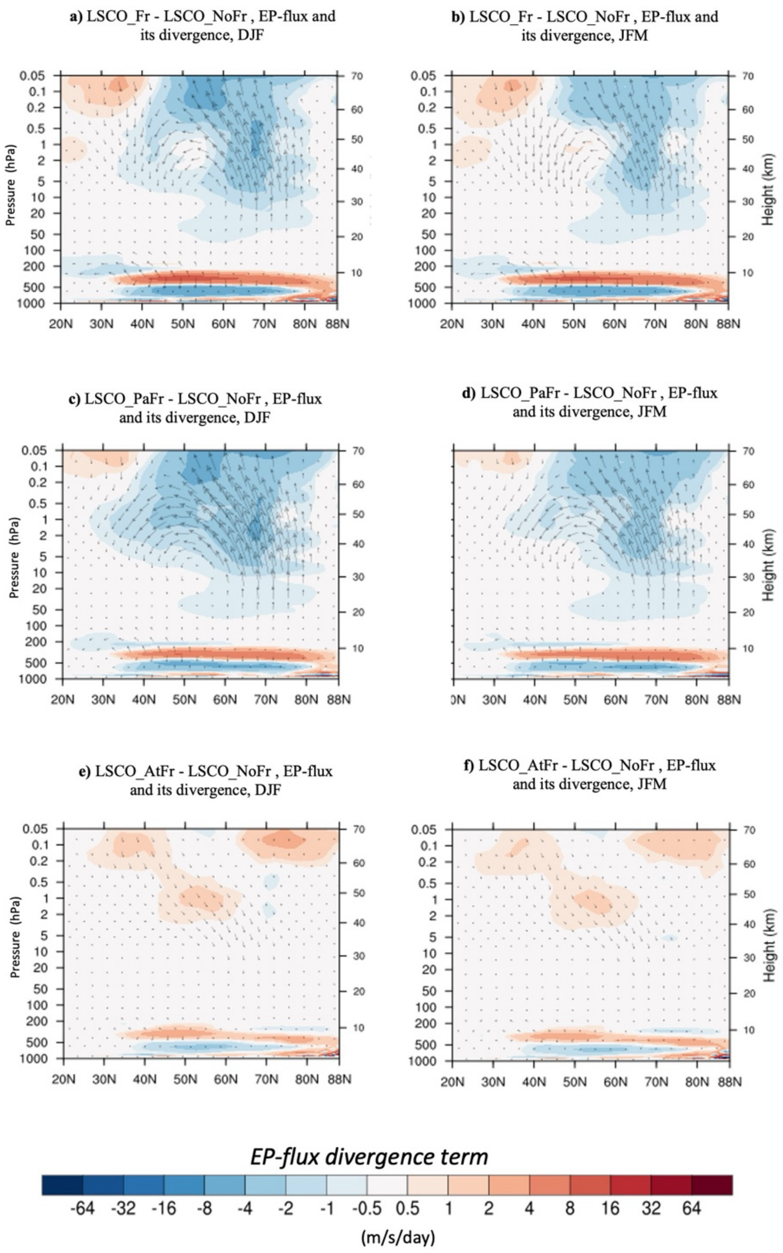

Supplementary Figure 8: EP-flux response to sea surface temperature (SST) fronts and its regional contributions: Zonal-mean response of the Eliassen–Palm (EP) flux and its divergence to SST fronts. Panels (a–b) show the overall response to the inclusion of SST fronts (LSCO_Fr – LSCO_NoFr) for December–to-February (DJF) and January–to–March (JFM), respectively. Panels (c–d) and (e–f) show the corresponding responses to the Pacific (LSCO_PaFr – LSCO_NoFr) and Atlantic (LSCO_AtFr – LSCO_NoFr) SST fronts, respectively. Shading denotes the anomalous EP-flux divergence (m s^{-1} day^{-1}), and arrows indicate the scaled anomalous EP-flux vectors. Blue (red) shading indicates anomalous convergence (divergence) of the EP-flux. Extending the analysis into the troposphere reveals that the Pacific SST-front experiment produces the stronger anomalous upward wave propagation from the troposphere into the stratosphere, linking tropospheric storm-track intensification with enhanced stratospheric planetary-wave activity.

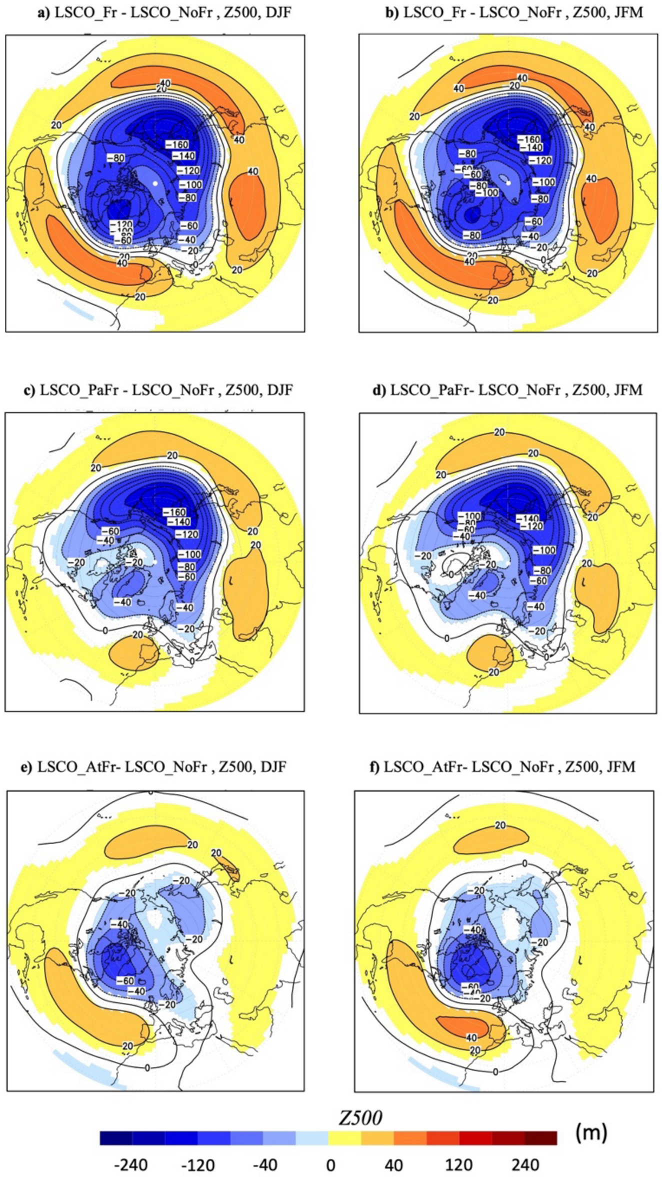

Supplementary Figure 9: Z500 response to sea surface temperature (SST) fronts and their regional contributions: Tropospheric circulation response to SST fronts, shown as 500 hPa geopotential height (Z500) anomalies (in m). Panels (a–b) display the overall response to the NH SST fronts (LSCO_Fr – LSCO_NoFr) for December–to–February (DJF) and January–to–March (JFM), respectively, while panels (c–d) and (e–f) show the Pacific (LSCO_PaFr – LSCO_NoFr) and Atlantic (LSCO_AtFr – LSCO_NoFr) SST front responses, respectively. Only statistically significant responses at the 95% confidence level (two-tailed Student's *t*-test) are shaded. Blue (yellow to red) shading indicates negative (positive) geopotential height anomalies. The Pacific SST-front response features the strengthened Aleutian Low, consistent with enhanced upward planetary-wave propagation and more frequent SSWs, while the Atlantic response exhibits a North Atlantic Oscillation (NAO)-like pattern that does not favor strong upward wave activity.

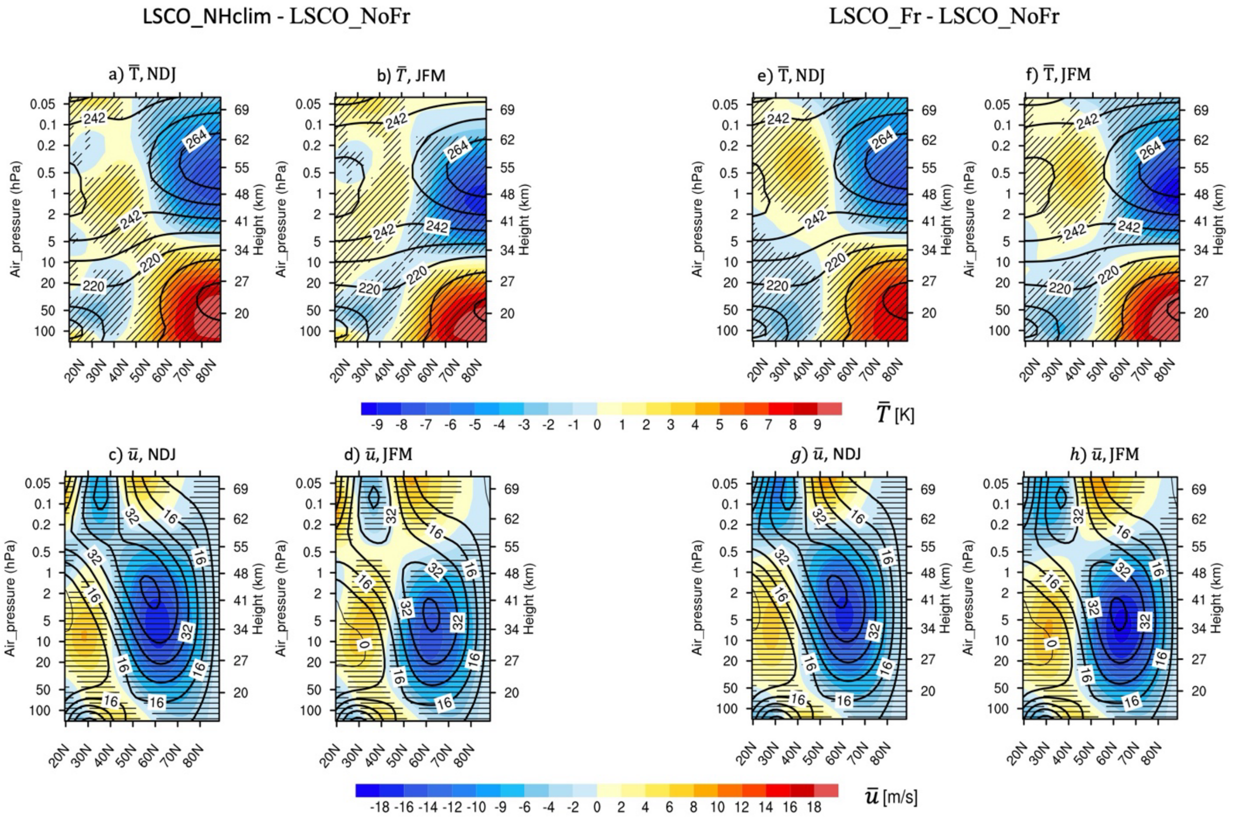

Supplementary Figure 10: Zonal-mean temperature and zonal-mean zonal wind responses to midlatitude sea surface temperature (SST) fronts and to the combined SST-front and sea-ice forcing: Panels (a–d) show the response when realistic Arctic sea-ice is added to the midlatitude SST fronts (LSCO_NHclim – LSCO_NoFr), while panels (e-h) show the response to midlatitude SST fronts alone (LSCO_Fr – LSCO_NoFr). The top row (a-b, e-f) displays zonal-mean temperature anomalies (K) for November–to–January (NDJ) and January–to–March (JFM), and the bottom row (c-d, g-h) shows zonal-mean zonal-wind anomalies (m s^{-1}) for the same seasons. Shading denotes anomaly amplitude and stippling marks regions significant at the 95% confidence level (Student's t-test). The solid contours represent the control simulation LSCO_NoFr. Both experiments exhibit a robust warming of the stratosphere up to ~5 hPa, associated with enhanced upward wave propagation, and a cooling in the uppermost stratosphere and lower mesosphere, consistent with a weakened residual circulation (not shown for the LSCO_NHclim experiment). The close similarity between the response of LSCO_NHclim and LSCO_Fr indicates that midlatitude SST fronts constitute the dominant driver of the stratospheric dynamical response, while the additional effects of sea-ice in LSCO_NHclim slightly modify but do not fundamentally change the mechanism.

Supplementary References

1. N.-E. Omrani *et al.*, Key Role of the Ocean Western Boundary currents in shaping the Northern Hemisphere climate. *Sci Rep-Uk* **9**, 3014 (2019).
