# Supplementary material for: Oceanic fronts shape hemispheric contrasts in polar stratospheric extremes
Source: Nat Commun. 2026 Apr 20;17:5462. doi: 10.1038/s41467-026-71998-5 (PMC13284378; doi:10.1038/s41467-026-71998-5)
Supplement: Supplementary file 2 — Transparent Peer Review file [file 41467_2026_71998_MOESM2_ESM.pdf]

nature portfolio

Peer Review File

Oceanic Fronts Shape Hemispheric Contrasts in Polar Stratospheric Extremes

Corresponding Author: Dr Nour-Eddine Omrani

This file contains all reviewer reports in order by version, followed by all author rebuttals in order by version.

Version 0:

Reviewer comments:

Reviewer #1

(Remarks to the Author)
Comments on “Oceanic Fronts Shape Hemispheric Contrasts in Polar Strato-spheric Extremes”
This manuscript investigates the influence of mid-latitude sea surface temperature (SST) fronts on polar stratospheric dynamics, focusing on their role in hemispheric asymmetries of Sudden Stratospheric Warmings (SSWs) and Polar Stratospheric Clouds (PSCs). Using semi-idealized simulations, the authors demonstrate that SST fronts—particularly in the Pacific—represent a previously overlooked driver, alongside traditional land-sea thermal contrast and orography (LSCO), for explaining Arc-tic-Antarctic differences in SSW frequency and PSC persistence. The study shows that SST fronts enhance upward planetary wave propagation, strengthening the Brew-er-Dobson Circulation (BDC) and causing high-latitude adiabatic warming, which in-creases Arctic SSWs while suppressing PSC formation. While offering novel insights into stratospheric extremes, the paper relies entirely on idealized model experiments without sufficient mechanistic analysis or observational validation. Below I outline ma-jor concerns that must be addressed before publication.

Major comments:
1. What is the spatial resolution of your monthly mean SST dataset? Which version of HadISST did you use? The 1-degree HadISST1 or the 5-degree HadiSST2? Your model’s resolution is T63, which is about 1.875 degrees, so even if you use the HadISST1, the efficient resolution is no higher than 1.875 degrees. Plus, you used monthly mean SST. All these facts mean that the SST front in your model is severe-ly smoothed, and the atmospheric impacts of the fronts are severely underestimated (Masunaga et al., 2015, JC; Parfitt et al., 2017, GRL; Zhang et al., 2020, Adv. At-mos. Sci.). Smirnov et al. (2015, JC) showed that the atmospheric (tropospheric) response to SST fronts in NCEP-NCAR (2.5°) is 40% weaker than in ERA-Interim (0.75°). I believe it’s necessary to increase the resolution to get the atmospheric re-sponse on the correct order of magnitude, even though the overall picture might be similar to what is found in this paper. In the paper it is written that some figures in the supplementary show the SST forcing in different sensitivity experiments. How-ever, as I cannot see the supplementary, I cannot really judge their correctness, but the limited resolution is nevertheless still a valid problem.
2. Howe does the oceanic SST fronts affect the stratosphere? It must first drive tropo-spheric response, and then in one way or another pass the response over to the stratosphere. The most promising bridge-mechanism could be the up-ward-propagating planetary waves associated with changed tropospheric westerly jet and/or storm track in response to the SST fronts. Studies have shown that the storm track response to SST fronts can indeed trigger planetary waves. The effects of the planetary waves are already evaluated with the TEM analysis, but the origin of the changing wave activity should be further explored, probably by simply ex-tending the TEM analysis down to the surface. Other possible bridge-mechanisms include differential orographic and non-orographic gravity waves and tropopause folding events, both presumably associated with SST frontal-driven storm track re-sponse. These tropospheric signals are already there in the model output. They must be included and analyzed in the paper to depict a full picture of stratospheric re-sponse to the sea surface, otherwise the paper is just “floating in the air”.
3. The whole paper is based on an aqua-planet model setup without any observational evidence, which is fine for a specialized paper in a specialized journal trying to un-derstand just a specific small piece of knowledge but is not enough for Nature Communications. More observational studies should be performed to justify the findings based on the model. For example, the authors could regress the BDC, PPSC, and SSW events in observational (e.g., SABER) or reanalysis dataset (e.g., ERA5 instead of NCEP for higher resolution) against SST fronts to show that SSW event frequency covaries with frontal strength and verify the mechanism in terms of the EP flux and others. The paper did include some basic results using the NCEP dataset in the supplementary (again, I cannot see the supplementary), but those are only for describing the phenomenon and no mechanism analysis is made thereby.

4. Some details about the experimental design and the analysis framework are not clear. 1) The captions of Fig. 1 and 2 claim that they show the seasonal evolution of westerly wind and PPSC, but why is the x-axis from November to May, covering only 6 months? 2) I thought SSW events primarily occur in NH winter, but why in Fig. 1 the zonal wind sign reversal happens more in spring. How could this happen? Or is there a mistake in the labels of the x-axis? 3) I understand the logic of the pa-per is that the EP flux divergence associated with LSCO and fronts drives reduction to the background westerly jet around the year, instead of their effects on individual events. Then, what is shown in Fig. 3-5? 62-year annual mean? Or winter mean? It should be made clear. 4) Is the model run continuously for 62 years forced by Had-ISST, or is it run for 62 separate periods spanning Nov-May? If the latter case (which I think is better expect for the seasons), how is the model initialized for each run? 5) How is the effects of land-sea thermal contrast and orography introduced into the SST? This is not described in the “Methods” section or anywhere in the paper. Is it shown in the supplementary? I have difficulties imaging adding the effect of orography just in SST. The land-sea thermal contrast could, I guess, probably be introduced by setting the SST to some typical land temperature over land. This must be clarified.
5. The model did not have sea ice, which could be a big problem. The authors argued that they removed sea ice on purpose to isolate the effects of SST fronts. But I think this is unreasonable because you just have to keep the sea ice boundary condition identical in the four experiments to achieve that. As long as the only thing different in the experiments is the SST front and/or the LSCO, you don’t have to worry about mixing different effects in the results. On the other hand, the most serious problem is regarding the model’s basic state and thermodynamic mechanisms responding to the midlatitude SST fronts. The absence of sea ice could force excessive upward heat flux at the surface, and cause erroneous low-level warming, weakened polar vortex, and increased SSW frequency. The propagation of planetary waves is affected too, which is essential to the BDC response. See Peings & Magnusdottir (2014, JC) and Kim et al. (2014, GRL) for the effect of sea ice. If you really want to keep the cur-rent simulation results, at least verify your model basic state.
6. The reason why the Pacific SST front is more effective should be discussed in more details. Is this front the Kuroshio-Oyashio Extension (KOE) front studied in many literature works? Is the Atlantic front the Gulf Stream (GS) front? Why are they dif-ferent? Is it because the KOE front is stronger, or is it because the Pacific is wider and its storm track is more powerful? As far as I know, this is somehow inconsistent with the findings of Kim et al. (2014) that the intensity of Atlantic storm track de-termines the frequency of SSW. Since the GS front modulates the Atlantic storm track, I would assume it has more impacts on the SSW too.

Minor comments:
1. The paper defines SSW events purely with wind reversals without considering reversals of meridional temperature gradient. Strictly speaking, this is not SSW events defined by WMO which requires both wind and temperature.
2. The paper uses words like “pivotal” and “critical” to describe the role of SST fronts, but does not give quantitative evidence supporting such strong conclusions. As said in the paper, the effect of SST fronts is much weaker than those driven by LSCO.
3. The colorbar of Fig. 5 is misleading. The blue part representing negative values range from -1.8 to 0 with a spacing of 0.2 (different with its positive part). It’s counterpart, Fig. 4, has the same color but ranges from -4.5 to 0. Without close inspection, the reader is easily deluded with an impression that the SST effects are of similar magnitudes as the LSCO effects. This little trick is not acceptable.
4. The Discussion section is just a lengthy repeat of the Results, with only a little bit discussion on implications and limitations in the last two paragraphs. Please shorten the review of results, extracting only the most saline findings, and add more discussions on important issues and comparison with previous studies.
5. The Method section is too verbose, including many sentences about background, purpose, or findings, that belong better to the introduction or results sections. Please in this section only describe the methods with concise language.
6. Caption of Fig. 4e: due to
7. Line 144: details -> detailed
8. Line 155: componeaffents? What’s that?

(Remarks on code availability)

Reviewer #2

(Remarks to the Author)
This is an interesting study showing the importance of oceanic fronts as drivers of stratospheric variability. The analysis is based on several idealized atmospheric-only experiments in which the effects of oceanic fronts is compared to the effect of land-sea contrast on stratospheric variability. The set of experiments include a pair of Aqua-Planet (AP) configurations and another pair with realistic orography (land-sea thermal contrast and orography, LSCO), both with and without oceanic fronts. Basin-specific contributions from the Pacific and the Atlantic are also assessed separately. Results highlight the overlook role of SST-fronts in driving stratospheric variability, and particularly in sudden stratospheric warming (SSW) occurrence and Polar Stratospheric Cloud formation, which appears to play a key role in the LSCO configuration. The observed impacts are explained via an acceleration of the residual mean circulation and therefore an adiabatic warming in the polar stratosphere. The manuscript is overall well-written and the messages are quite clear. Particularly interesting is the focus on Polar Stratospheric Cloud formation in the Arctic stratosphere since this is a significant issue considering the uncertainties in the future trends of the Northern Stratospheric Polar vortex. However, there are several unclear points that need clarification before publication.

I think too much attention is paid to separate the effects of resolved and non-resolved wave forcing. I understand this is a common procedure in this kind of analysis, but it should be acknowledged that this is quite model dependent, and very much depends on the particular parametrization of gravity waves used. I specifically missed some comments on the

parametrization used if the specific contribution of resolved vs. parametrized waves is considered to be so important. Moreover, the conclusion was somehow expected, since by wave filtering, as you explain in the manuscript, non-resolved waves show a major role in the upper stratosphere and mesosphere, while resolved waves mainly force the stratospheric circulation. In fact, note that this result is not even mentioned in the abstract. I still think this analysis is correctly developed through the paper, and although it is valuable, it takes up too much space, and more priority should be given to explain the mechanisms leading to the results.
In particular, less attention is paid to the large changes in the background winds in the different configurations and how this may affect wave propagation. In principle, in the experiments, you are modifying wave generation: including or not those related to fronts in Fr/NoFr cases, and large-scale Rossby wave trains in the AP/LSCO configurations. In addition, upward wave propagation from the lower stratosphere is also modified via changes in the background flow: conditions may be very different in the Aqua Planet vs. the LSCO experiment; even we are not shown the different wind profiles, it can be inferred both Figure 1, or the already large differences in LSCO_NoFr and LSCO_Fr in Figures 3c and 3d. It would be helpful to quantify the impacts of LSCO and SST fronts on the background conditions to interpret the role that the increased front-related wave forcing has on SSWs. Are these waves just modifying the background conditions or are they directly responsible for triggering SSW? This is important because from Figure 1, one could interpret that they are responsible for SSW triggering – they have a major impact on the variability than on the mean wind value, but SSW occurrence is largely associated with resolved waves, how is this reconciled?
In this sense, line 12 reads "This warming significantly enhances Arctic SSW frequency and strongly reduces PSC formation."
From this statement, one could assume that SST fronts are responsible for background wind changes that favor conditions for SSWs, but, is this shown? As previously commented, Figure 1 shows that the largest effect on the mean flow is LSCO, and the effect of fronts mainly acts to increase the variability, which is more effective in producing SSWs because winds are already weaker: closer to zero (cf. Figure 1 left and right panels). Again, more details are needed in the specific role of fronts on SSW occurrence.

Minor comments
Another, although minor concern because should not change the conclusions of the analysis is that possible biases in the location of the stratospheric polar vortex are not taken into account in Figure 1. Note that Figure 3 shows an equatorward bias of the maximum strength of the winds, so it would be misleading to use this same unique latitude (60N) to evaluate the stratospheric polar vortex in all the experiments, and another approach as a latitudinal average would be more precise.
It should be specified in the abstract that the experiments are semi-idealized because not including sea ice is an important fact considering the number of studies that relate stratospheric polar vortex interactions to Arctic sea-ice (e.g. recently, Liang et al 2024 or Xu et al. 2024).
Line 141: Have you considered using NRWF or non-RWF? Using RWF vs NRF is confusing to me.
Lines 163-165: Is it balanced by anomalous diabatic cooling? This is confusing, Are you just referring to a particular equation?
Lines 200-201: "However, its influence is weaker compared to the LSCO effects." Aren't the LSCO effects translated into RWF? Or you mean the total, TWF?
Lines 207-210: I very much agree with this statement but it's quite repetitive as in lines 179-181
Line 645: "Negative dynamical forcing drives positive Coriolis forcing". This does not seem accurate since that momentum does not drive Coriolis forcing, more accurate is that the Coriolis forcing term (the term) increases.
Line 191,192 and other parts of the text: LSCO-effects, SSW-frequency and PPSC-conditions, I don't think you need the hyphen in these cases because they're not a compound adjective followed by a name.
Discussion:
Interesting to see how oceanic fronts become so important when LSCO is present. In this sense, I am missing some discussion on the role of orography that has been explored before (e.g. White et al. 2018).

Detected typos:

Line 59: Do you mean wave-mean flow interactions? Please, revise
Line 144: details -> detailed
Line 151: please revise "componeaffents accelerate"
Line 259: Ozon -> Ozone
Line 216,308: "conductive" conducive, as you used in other parts of the text, is better.
Line 285: SSW -> SSWs
Line 296: "which as we will show" change for "which as shown in Results" or similar since Methods appear at the end of the manuscript.
Line 316: AP_NoFr-configuration -> AP_NoFr configuration
Line 352: Eulerian
Line 368: temperature
Line 380: "residuum"
Line 397: "term.."
Line 401: origin
Figures 3-5: y-axis label reads "Air_pressur", revise.
Figure 5a: there's a white arrow in the bottom right, does this mean something?
Figure 5d is missing the Height values.
Figure 6: The vertical axis reads Height, but it should be Pressure level.
Supplementary Material: Please, revise the formatting of figure captions.

REFERENCES:
Liang, Y. C., Kwon, Y. O., Frankignoul, C., Gastineau, G., Smith, K. L., Polvani, L. M., ... & Screen, J. (2024). The weakening of the stratospheric polar vortex and the subsequent surface impacts as consequences to Arctic sea ice loss. Journal of Climate, 37(1), 309-333.
White, R. H., Battisti, D. S., & Sheshadri, A. (2018). Orography and the boreal winter stratosphere: The importance of the Mongolian mountains. Geophysical Research Letters, 45(4), 2088-2096.
Xu, M., Screen, J. A., Tian, W., Zhang, J., Zhang, C., & Yu, H. (2024). Influence of regional sea ice loss on the Arctic stratospheric polar vortex. Journal of Geophysical Research: Atmospheres, 129(15), e2023JD040571.

(Remarks on code availability)

Reviewer #3

(Remarks to the Author)
This work aims to isolate and understand the impact of midlatitude SST-fronts on the frequency of SSWs and climatic conditions conducive to the PSCs. However, after reading the entire manuscript, I find that neither of the two stated goals is clearly addressed.

(A) The impact of SST-fronts cannot be considered in isolation, as it is inherently influenced by land-sea thermal contrast and orography.

In this work, the authors 'isolate' the impact of SST-fronts by calculating the differences between LSCO_Fr and LSCO_NoFr (Fig. 3 b&d and Fig. 5). This difference shows the impact of midlatitude SST-fronts with interference from land-sea thermal contrast and orography (LSCO). First of all, what the authors should do is to calculate the difference between AP_Fr and AP_NoFr, which represent the impacts of SST fronts in the absence of LSCO. Beyond that, I don't think the impact of SST-fronts can be isolated, given the non-linear interaction between SST-fronts and LSCO. The non-linear interaction between SST-fronts and LSCO can be inferred from Supplementary Table 1. In particular, AP_NoFr vs. AP_Fr suggests that adding SST-fronts alone does not substantially increase SSW-frequency; AP_NoFr vs. LSCO_NoFr suggests that adding LSCO alone also does not substantially increase SSW-frequency. It is only when both SST-front and LSCO (LSCO_F) are included that one can see an SSW frequency comparable to the observations.

(B) The study relies heavily on model simulations but lacks robust theoretical justification.

The findings of this study are largely derived from a single model's experiments. Although the model results show some agreement with reanalysis (Figs. 1-2), such agreement can arise by coincidence or may stem from compensating errors, rather than an accurate representation of the underlying physical processes. Therefore, a comprehensive theoretical framework is required to support the robustness of the results. However, this study lacks a thorough examination of the underlying mechanisms behind the results. In the absence of observational and theoretical support, modeling results alone cannot provide conclusive scientific insight. In fact, the manuscript lacks a clear logical structure, well-articulated scientific ideas, and a rationale analytical foundation. For example, regarding "What drives the North-South asymmetry of SSWs and PSCs?", after briefly introducing the role of orography and land-sea thermal contrast first, the authors jumped to say "Here, we propose a crucial missing link: Sea Surface-Temperature (SST) fronts" (see lines 61-67). What is your scientific thinking behind this proposal? How can you expect it to be 'crucial'? Without a clear explanation of the scientific rationale behind this proposal, it risks appearing as an arbitrary choice, lacking a meaningful physical connection to the core topic of the study.

(C) The manuscript has a highly specialized focus and may not meet the general scope of the journal.

The manuscript mentions PSCs and stratospheric ozone depletion but does not provide a direct or detailed analysis of these topics. The focus on temperature and wind anomalies may limit its relevance to the wider scope of the journal.

Other comments:

Line 3: "such as Sudden Stratospheric Warmings (SSWs)" ->

such as major Sudden Stratospheric Warmings (SSWs)

Lines 3-4: "Extreme stratospheric events such as ... Polar Stratospheric Clouds (PSCs)"

The term "Polar Stratospheric Clouds (PSCs)" generally refers to a phenomenon rather than an event. This also applies to line 272.

Lines 49-51: I'm not sure about the relevance of mentioning SSW in this sentence. Is the first part of this sentence necessary?

Line 55: "The BDC is typically stronger in the NH than in the SH winter."

Please provide some references for this statement.

Lines 55-56: "A strong BDC drives polar adiabatic warming and weaker polar vortex, ..."

maybe say "In the NH, a strong BDC drives polar adiabatic warming and weaker polar vortex, ... "

Lines 66-67: "These sharp oceanic temperature gradients, associated with western boundary currents, play pivotal roles in atmospheric dynamics (33-38)."

Please describe how the SST fronts impact atmospheric dynamics.

Lines 69-70: "While previous studies have explored their role in shaping the tropospheric eddy-driven jets, storm-tracks, blocking events and stratospheric polar vortex"

Please provide some references for these statements, as well as describe how the SST fronts shape these systems.

Lines 129-130: "anomalous negative windshear (increasing easterly anomalies with height), whereas anomalous negative thermal gradient in the uppermost stratosphere and mesosphere corresponds to anomalous positive windshear (decreasing easterly anomalies with height)."

The correspondence is not that clear here. It is not easy to imagine the picture of a windshear based on the figure shown.

Lines 131-132: "Adding SST-fronts overall amplifies these patterns,"

The impact of adding SST-fronts can also be shown by calculating the difference between AP_Fr and AP_NoFr. Do you get the same result?

Lines 133-134: "While these responses are weaker compared to those to LSCO"

How do you reach this conclusion? Did you calculate the difference between a&b, as well as between c&d?

Lines 157-158: "across the extratropical stratosphere, resulting in notable weakening of the westerlies"

Do you show the changes of zonal winds here? If not, please plot a box in Fig. 4 to indicate the domain of change in dynamical drag, as well as a box in Fig. 3 to indicate the domain of change in zonal wind. This will allow the readers to see if there is a correspondence between the two.

Line 159: "which corresponds to the strengthening of the BDC, particularly its downward branch"

You don't expect all readers to understand BDC and its downward branch. To make it easier for your readers to follow, please add a box to show where you refer to (e.g., where the downward branch is). This will allow the readers to know

where they should focus on your figures, especially when most of the figures are busy and noisy.

Line 160: "drives mid-to-high latitude adiabatic warming (Fig. 4d),"

Please indicate the altitude of this warming. Again, add a box to indicate where you refer to.

Lines 168-169: "This cooling explains the mid-to-high latitudes mesospheric temperature reduction."

I don't think there is a close correspondence between the changes in residual circulation and the changes in temperature. For example, the former is strongest at about 50-60N, whereas the latter is strongest at 90N.

Lines 169-170: "The overall vertical structure of the zonal wind response remains consistent with the meridional gradient of adiabatic temperature-changes,"

Again, this is not as obvious as the authors thought.

Lines 172-174: "RWF emerges as the primary driver of the stratospheric BDC-response in response to LSCO (Fig. 4b-f), intensifying its subsidence branch and inducing stronger adiabatic warming in mid-to-high latitudes."

"primary"? Panel b does not resemble panel a. Also, panel e does not resemble panel d.

Lines 186-187, "The midlatitude SST-fronts exert significant influence on SSWs and PPSCs (Figs. 1&2), although their effects are weaker than those driven by LSCO (Fig. 4)."

This appears to be true, as suggested by the figures, but is not supported by the table. According to Supplementary Table 1, if one compares LSCO_NoFr and LSCO_Fr, one may conclude that the largest changes in SSW frequency are caused by adding the SST-fronts.

Lines 233-234: "The Pacific SST-front emerges as the dominant driver of these changes, while the Atlantic SST-fronts exert minor impact. This underscores the importance of basin-specific SST-gradients."

Why? Any dynamic explanation?

Lines 76-77 vs. Lines 314-316: In lines 76-77 "In AP_Fr a symmetric SST-front is introduced in AP_NoFr-configuration"; in lines 314-316: "The Aqua-Planet with SST-fronts (AP_Fr) experiment adds zonally averaged SST-fronts over the western Atlantic boundary current to the AP_NoFr-configuration,"

I don’t think these two statements are identical. What kind of SST-fronts are indeed used?

Fig. 3 b&d: the two panels represent the impacts of SST fronts in the presence of land-sea thermal contrast and orography. Can you also show the difference between AP_Fr and AP_NoFr, which represent the impacts of SST fronts in the absence of land-sea thermal contrast and orography? This also applies to the entire Fig. 5: you can also show the differences between AP_Fr and AP_NoFr, which also represent the impacts of SST fronts but in the absence of land-sea thermal contrast and orography.

(Remarks on code availability)

Version 1:

Reviewer comments:

Reviewer #1

(Remarks to the Author)
The authors have revised the manuscript according to the reviewers' comments. I have no further comments.

(Remarks on code availability)

Reviewer #2

(Remarks to the Author)
I would like to thank the authors for the detailed responses to my comments. My main concern in the previous revision was the role of SST Fronts in SSW triggering and/or modification of the background flow and how it was explained. Authors have clarified that the major role of SST Fronts is on the background flow, and that leads to increased variability. They have modified some passages of the text and included more supplementary figures to support the impact of Pacific SST Fronts as a precursor for weak vortex regimes.
The remainder of the comments have been satisfactorily resolved, so I can recommend the publication of the manuscript after checking some very minor notes below.

L3: MSSW; You should choose MSSW/SSW and use it thorough the paper, SSW is a better option since it’s the standard.
L30: Majore -> Major
L31: 50C is quite too specific and it’s not part of the definition; rapid warming could be enough here.
L57-58: This sentence leads to the understanding the BDC is responsible for the different polar vortex strengths between the Northern and the Southern Hemispheres. It would be more correct to say that both, background wind configurations and the BDC are driven by wave forcing, and indeed, weak-vortex years correlate with enhanced BDC strength and increased polar ozone due to stronger poleward transport (Harzer et al., 2023: https://doi.org/10.5194/acp-23-10661-2023), but this is interpreted as co variability driven by waves, rather than a cause-> consequence: stronger BDC -> weak polar vortex. Please, clarify
L55-57: These three lines need some revision, it feels choppy, you could try to connect them.
L60: wavea-> wave
L64: Please, correct: “tropical (mid-to high) latitudes”
L100: In AP_Fr -> For AP_Fr
L101: in (the) AP_NoFr
L120: srong -> strong
L141 and 142: PPSC is already defined in L131.
L159: In your response to Rev#1, Figure R1Fig6 seems to be in agreement with results in Palmeiro et al., (2025: https://doi.org/10.1029/2025GL116639)regarding covariability over the Kuroshio-Oyashio extension and the polar vortex.
L174: proportional “to”
L200: accelerate of -> accelerate
L200: reduces -> reduce
L244: “shifting stratospheric dynamics”; Shifting where?
L253: high-latitudes -> high-latitude
L320: use of “too strong”; this sentence could be improved
L321: “but not enough…” -> but insufficient to account for the observed…
L323: upward propagation -> upward wave propagation
L364: theire -> their

(Remarks on code availability)

Open Access This Peer Review File is licensed under a Creative Commons Attribution 4.0 International License, which permits use, sharing, adaptation, distribution and reproduction in any medium or format, as long as you give appropriate credit to the original author(s) and the source, provide a link to the Creative Commons license, and indicate if changes were made.
In cases where reviewers are anonymous, credit should be given to 'Anonymous Referee' and the source.
The images or other third party material in this Peer Review File are included in the article's Creative Commons license, unless indicated otherwise in a credit line to the material. If material is not included in the article's Creative Commons license and your intended use is not permitted by statutory regulation or exceeds the permitted use, you will need to obtain permission directly from the copyright holder.
To view a copy of this license, visit https://creativecommons.org/licenses/by/4.0/

We thank the Editor for handling our manuscript and for the opportunity to revise it. We also thank the reviewers for the time and effort they devoted to carefully reviewing this manuscript. Their thoughtful and constructive comments were extremely helpful and have led to substantial improvements in the clarity, structure, and scientific depth of the paper.

In response to the reviews, we have done our best to address each comment carefully. In particular, we have (i) strengthened the mechanistic interpretation by explicitly linking SST fronts to tropospheric precursor patterns and upward planetary-wave propagation and considering the role of the background state, (ii) clarified the physical rationale and linear consistency of the experimental design, (iii) added new diagnostics and figures to trace the troposphere–stratosphere coupling pathway, (iv) assessed the robustness of our conclusions across model resolutions and within a coupled framework, and (v) clarified the role and limitations of observational evidence in the context of our mean-state focus. We also realized that, to be consistent with previous studies, the entire month of March must be included in SSW. This leads to slightly larger difference in SSW-events between SST-front and non-front experiments, ensuring the Robustness of the SSW response to SST-fronts. We believe these revisions have significantly improved the manuscript and we hope they directly address the reviewers' concerns regarding mechanism, interpretation, and relevance. If any issues remain unresolved, we would be happy to address them further.

For clarity and ease of navigation, the response to each reviewer is presented as a self-contained section, with individual comments followed directly by the corresponding responses and relevant references. **Throughout this document, reviewer comments are shown in "red", our responses are shown in "black", and summaries of the corresponding changes made to the manuscript are shown in "blue". The detailed response to Reviewer 1 begins on page 1, the response to Reviewer 2 begins on page 26, and the response to Reviewer 3 begins on page 37.**

Reviewer #1 (Remarks to the Author):

Comments on "Oceanic Fronts Shape Hemispheric Contrasts in Polar Strato-spheric Extremes"
This manuscript investigates the influence of mid-latitude sea surface temperature (SST) fronts on polar stratospheric dynamics, focusing on their role in hemispheric asymmetries of Sudden Stratospheric Warmings (SSWs) and Polar Stratospheric Clouds (PSCs). Using semi-idealized simulations, the authors demonstrate that SST fronts—particularly in the Pacific—represent a previously overlooked driver, alongside traditional land-sea thermal contrast and orography (LSCO), for explaining Arc-tic-Antarctic differences in SSW frequency and PSC persistence. The study shows that SST fronts enhance upward planetary wave propagation, strengthening the Brew-er-Dobson Circulation (BDC) and causing high-latitude adiabatic warming, which in-creases Arctic SSWs while suppressing PSC formation. While offering novel insights into stratospheric extremes, the paper relies entirely on idealized model experiments without sufficient mechanistic analysis or observational validation. Below I outline ma-jor concerns that must be addressed before publication.

We thank the Reviewer 1 for the time and effort devoted to reviewing this manuscript and for the detailed and constructive comments. The reviewer's comments helped improve the manuscript considerably. In particular, they motivated us to clarify the physical rationale and linear consistency of the experimental design, to strengthen the mechanistic interpretation linking SST fronts to tropospheric precursor patterns and upward planetary-wave propagation, and to assess the robustness of our conclusions across model resolutions and coupled configurations. These revisions have substantially improved the clarity, rigor, and interpretability of the manuscript.

Major comments:

1. What is the spatial resolution of your monthly mean SST dataset? Which version of HadISST did you use? The 1-degree HadISST1 or the 5-degree HadiSST2? Your model's resolution is T63, which is about 1.875 degrees, so even if you use the HadISST1, the efficient resolution is no higher than 1.875 degrees. Plus, you used monthly mean SST. All these facts mean that the SST front in your model is severe-ly smoothed, and the atmospheric impacts of the fronts are severely underestimated (Masunaga et al., 2015, JC; Parfitt et al., 2017, GRL; Zhang et al., 2020, Adv. At-mos. Sci.). Smirnov et al. (2015, JC) showed that the atmospheric (tropospheric) response to SST fronts in NCEP-NCAR (2.5°) is 40% weaker than in ERA-Interim (0.75°). I believe it's necessary to increase the resolution to get the atmospheric re-sponse on the correct order of magnitude, even though the overall picture might be similar to what is found in this paper. In the paper it is written that some figures in the supplementary show the SST forcing in different sensitivity experiments. How-ever, as I cannot see the supplementary, I cannot really judge their correctness, but the limited resolution is nevertheless still a valid problem.

We thank the reviewer for this thoughtful comment. We apologize for the missing Supplementary figures, which also depict the SST-front in each of the experiments and provide clearer insight into the purpose of the experimental setup. The Supplementary Material was uploaded under an incorrect name.

We fully agree that model resolution can play a role in modulating the strength of the atmospheric response to SST fronts, especially in mesoscale-sensitive regions such as western boundary current extensions. However, our study does not aim to resolve fine-scale SST fronts or their detailed impacts. As mentioned in the title and abstract and as becomes clear from the Supplementary figures (depicting the SST used for the non-front experiment, SFig. 1), we focus on a different scientific question: which large-scale lower boundary structures (e.g., LSCO and overall SST fronts) are responsible for the hemispheric asymmetry in SSW and PSC frequency that occurs in both observations and our standard idealized model resolution with the stratosphere included (SFig. 2-3 & SFig. 5-7 that are also presented as R1Fig.1-3 below), see also details below). The focus is on the existence of the SST front and land-sea contrast with orography and not on their highly resolving structures. The SST fronts imposed in our experiments are broad-scale features shaped by gyre-scale ocean circulation with basin scales, and our interest lies in their presence or absence as done for LSCO, not the detailed gradient sharpness.

The smoothing almost removes the SST gradient by assuming that SST north of 33.5°N decreases linearly, with the gradients in the smallest range of SST-gradient north of 33.5°N. In this way, the localized gradients along the Gulf Stream and Kuroshio currents are completely removed (see Methods and SFig. 1 and c). This is very different from standard smoothing approaches, which keep local SST-gradien and just smooth it into smaller values. This smoothing enables the study of the impact of SST fronts in a standard-resolution model but compared to the standard smoothing can conflict with Arctic sea-ice induced SST-fronts (see the comment related to the Arctic sea ice). This approach of removing SST fronts in our model experiments represents an extreme case designed to maximally suppress SST-front effects. It is therefore comparable, in terms of experimental severity, to removing land–sea thermal contrast and orography (LSCO), allowing us to robustly assess the full role of SST fronts in the Northern Hemisphere.

We acknowledge that, although this goal is clearly stated in the title and abstract, it may not have been articulated with sufficient clarity in the main text, particularly in the early framing of the Methods section. We further recognize that the absence of the Supplementary Information during the review process may have hindered a clear interpretation of our experimental design and the rationale behind our resolution choices.

To verify that the hemispheric asymmetry we describe is not an artifact of coarse resolution, we performed an additional sensitivity test using the coupled MPI-ESM at two CMIP-class resolutions (SFig. 5–7 or R1Fig. 1-3 below):

- a lower-resolution configuration with a T63 atmosphere (matching the resolution of our idealized experiments) and a ~100 km ocean,
- a higher-resolution configuration with a T127 atmosphere and a ~40 km ocean

Just like the NCEP date (Sfig. 2-3), Both configurations reproduce a clear (SFig. 5-7 or R1Fig. 1-3) NH > SH asymmetry in SSW frequency (Fig. 1.1) and a NH < SH asymmetry in PSC occurrence. The higher-resolution model exhibits slightly more frequent SSWs and slightly fewer PSCs compared to the standard resolution, which is consistent with the impact of SST fronts identified in our idealized experiments. This behavior confirmins that the hemispheric asymmetry between the NH and SH is robust and independent of model resolution. A closer comparison with reanalysis data (NCEP/ERA; Supplementary SFigs. 2–3) shows that the lower-resolution configuration, agrees more closely with the observed climatology than the higher-resolution configuration. A likely reason is that the gravity-wave parameterization in ECHAM was originally tuned at T63 resolution, whereas the higher-resolution version was not re-tuned. A detailed assessment of this issue is beyond the scope of the present study.

Overall, while increasing resolution slightly change the response, the hemispheric contrast remain unchanged, demonstrating that our conclusions regarding the role of SST fronts in shaping the zonal asymmetry of SSWs and PSCs depend primarily on LSCO and the large-scale structure of SST fronts, with higher SST resolution acting to slightly strengthen—rather than alter—the response in term of NH-SH asymmetry.

In our experiments, we use HadISST1, which provides 1° spatial-resolution SST climatology, interpolated to the model's T63 spectral resolution (~1.875°). As noted in the reviewer comments, this standard T63 resolution is sufficient for studying the NH–SH asymmetry in SSW and PPSC occurrence, even though it results in a smoothed representation of SST gradients. The use of monthly mean climatological SSTs may further contribute to temporal smoothing. However, results from the fully coupled MPI-ESM simulation at comparable atmospheric resolution (T63)—which includes hourly atmosphere–ocean coupling—exhibit nearly the same hemispheric asymmetry in SSW and PSC frequency as our prescribed monthly SST-front experiments. This close agreement indicates that the frequency of SST–atmosphere coupling has only a minor influence on the NH–SH asymmetry in SSWs and PPSC representation. When model resolution is increased (see R1Fig. 1–3 and the same figure in SFig. 5–7), the hemispheric asymmetry becomes slightly stronger, consistent with our idealized experiments showing that enhanced SST-front representation amplifies the response. Taken together, these results demonstrate that the hemispheric asymmetry primarily arises from the presence or absence of large-scale SST fronts and land–sea–orography contrasts, rather than from coupling frequency or finer spatial resolution alone.

Changes made in response to the comments:

We have clarified this distinction and modeling rationale in both the **Methods** and **Discussion** sections and ensured that the **Supplementary Information**, which includes the relevant SST maps, becomes apparent. In particulare:
We clarified in the Methods that SSTs are interpolated to the model resolution prior to integration (Lines. 375-376) and explicitly stated that the aim of the study is to identify *large-scale* lower-boundary controls (LSCO and SST fronts) on hemispheric SSW and PSC asymmetry in standard resolution model (Lines 83-89 and Lines 107-111 in the mean text and Lines 378-381 in the methods). We further justify the use of a semi-idealized T63 framework by showing that the NH–SH asymmetry is robust across both lower and higher standard resolutions, provided the stratosphere is well resolved and appropriate gravity-wave parameterizations are included (Lines 85-87 in the mean text and Lines 381–387 in methods; SFig. 5–7).

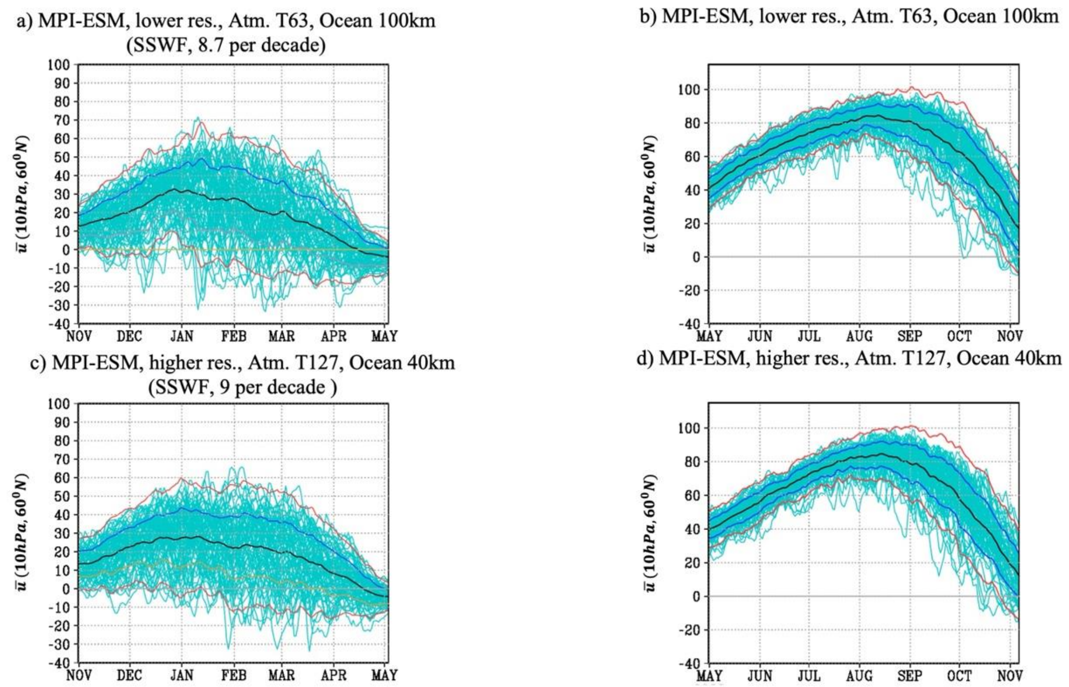

R1Fig. 1 Seasonal evolutions of the stratospheric zonally averaged zonal wind (MPI-ESM coupled simulations): Like Sfig. 2, these panels show the seasonal evolution of the zonally averaged westerly wind (u) at 10 hPa and 60° in the (a,c) Northern Hemisphere (NH) and (b,d) Southern Hemisphere (SH). Panels (a,b) correspond to the lower-resolution configuration (T63 atmosphere, ~1.9°, 100 km ocean), and (c,d) to the higher-resolution configuration (T127 atmosphere, ~1.0°, 40 km ocean). The black lines indicate the mean seasonal cycle, while the blue and red lines represent the ±1 and ±2 standard-deviation margins, respectively. The dark yellow horizontal line denotes the zero-wind threshold, marking the transition from westerly to easterly flow associated with Sudden Stratospheric Warmings (SSWs). The mean SSW frequency is **0.87 yr^{-1}** in the lower-resolution model and **0.90 yr^{-1}** in the higher-resolution model, demonstrating that the simulated hemispheric asymmetry and SSW variability are robust to changes in model resolution

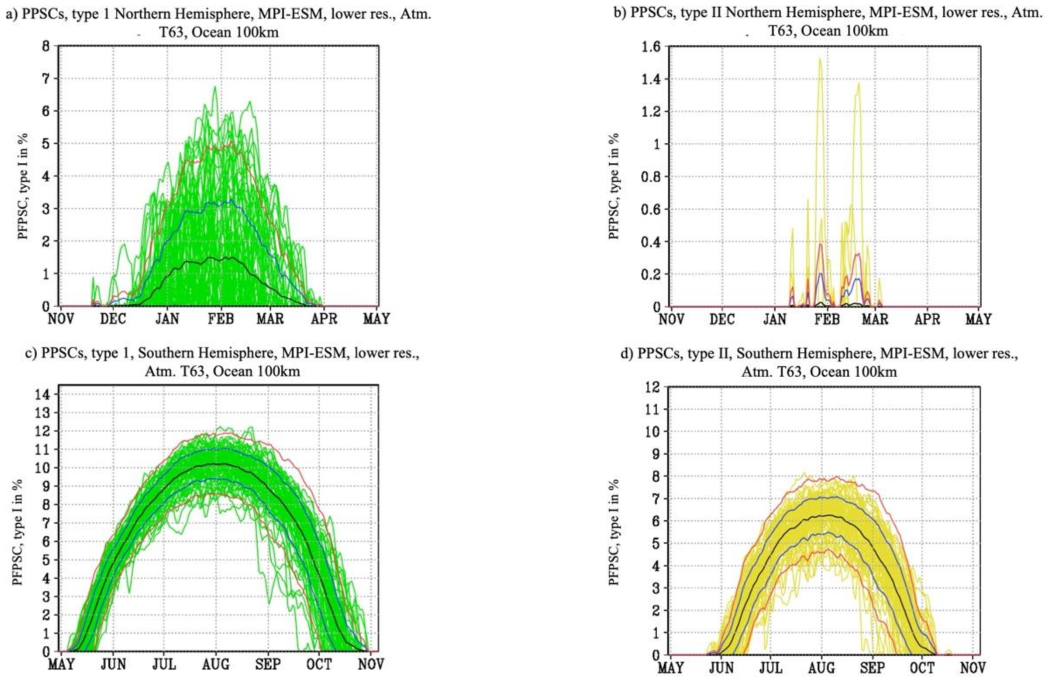

R1Fig. 2 Seasonal evolutions of the stratospheric zonally averaged zonal wind (MPI-ESM coupled simulations): Like Sfig. 2, these panels show the **Seasonal evolutions of PPSC type I and II (MPI-ESM, lower resolution):** Like Fig. 2, these panels illustrate the seasonal evolution of the potential formation of polar stratospheric clouds (PPSC; see Methods) for the Northern and Southern Hemispheres in the **MPI-ESM lower-resolution configuration** (T63 atmosphere, ~1.9°, 100 km ocean). Panels (a) and (b) show PPSC type I and type II for the **Northern Hemisphere**, while panels (c) and (d) depict PPSC type I and type II for the **Southern Hemisphere**. PPSC I and II correspond to the percentage of hemispheric area where temperatures fall below 195 K and 188 K, respectively. The black lines indicate the mean seasonal cycle, and the blue and red lines represent the ±1 σ and ±2 σ variability, respectively. The simulation reproduces the strong hemispheric asymmetry in PSC occurrence, with frequent PSC conditions in the SH and rare formation in the NH, consistent with the modeled SSW climatology.

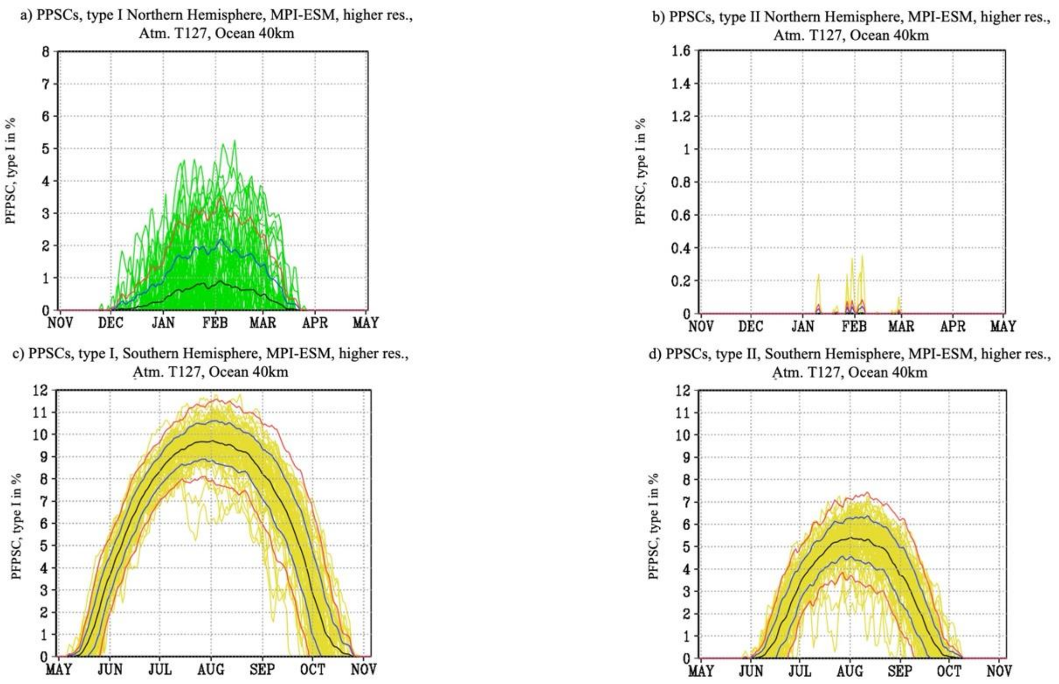

R1Fig. 3 Seasonal evolutions of PPSC type I and II (MPI-ESM, higher resolution): These panels illustrate the seasonal evolution of the potential formation of polar stratospheric clouds (PPSC; see Methods) for the Northern and Southern Hemispheres in the **MPI-ESM higher-resolution configuration** (T127 atmosphere, ~1.0°, 40 km ocean). Panels (a) and (b) show PPSC type I and type II for the **Northern Hemisphere**, and panels (c) and (d) show PPSC type I and type II for the **Southern Hemisphere**. PPSC I and II correspond to the percentage of hemispheric area where stratospheric temperatures fall below 195 K and 188 K, respectively. The black line indicates the mean seasonal cycle, while the blue and red lines represent the ±1 σ and ±2 σ variability, respectively. The higher-resolution configuration exhibits a slightly reduced overall PPSC occurrence—particularly in the Northern Hemisphere—consistent with enhanced resolved planetary-wave activity and the increased SSW frequency compared to the lower-resolution simulation.

2. Howe does the oceanic SST fronts affect the stratosphere? It must first drive tropo-spheric response, and then in one way or another pass the response over to the stratosphere. The most promising bridge-mechanism could be the up-ward-propagating planetary waves associated with changed tropospheric westerly jet and/or storm track in response to the SST fronts. Studies have shown that the storm track response to SST fronts can indeed trigger planetary waves. The effects of the planetary waves are already evaluated with the TEM analysis, but the origin of the changing wave activity should be further explored, probably by simply ex-tending the TEM analysis down to the surface. Other possible bridge-mechanisms include differential orographic and non-orographic gravity waves and tropopause folding events, both presumably associated with SST frontal-driven storm track re-sponse. These tropospheric signals are already there in the model output. They must be included and analyzed in the paper to depict a full picture of stratospheric re-sponse to the sea surface, otherwise the paper is just “floating in the air”.

We thank the reviewer for this insightful comment regarding the mechanism linking oceanic SST fronts to the stratospheric circulation. We didn’t include it to keep the paper as simple as possible.

However, we fully agree about clarifying the tropospheric origin of the planetary waves that ultimately drive the stratospheric response and we are happy to go deeper on that.

We have now extended the Eliassen–Palm (EP) flux analysis downward into the lower atmospheric levels (Supplementary Fig. 8; R1Fig. 4 below). As expected, the new figures confirm that stronger upward propagation of planetary waves from the lower into the upper atmospheric levels occurs in the presence of SST fronts, with the response being dominated by the Pacific sector.

The additional analysis (Supplementary SFig. 9; R1Fig. 5 below) further shows that the SST fronts—particularly in the Pacific—lead to a pronounced strengthening of the North Pacific low geopotential height anomalies. As demonstrated in previous studies (Garfinkel and Hartmann 2008, Ineson and Scaife 2009, Nishii, Nakamura et al. 2009, Garfinkel, Butler et al. 2012), the strengthening (weakening) of low geopotential height anomalies is a well-known precursor pattern for stratospheric vortex weakening (strengthening) enhancing (reduceing) upward planetary-wave propagation. Therefore, the increase of North Pacific low geopotential height anomalies in response to SST-front serves as intensifier of upward wave propagation and therefore precursor for vortex weakening and associated SSWs.

This strengthening of the north Pacific low geopotential height anomalies is dynamically consistent, with the intensification of the Pacific eddy-driven jet (as shown in Omrani et al. 2019), which arises from enhanced baroclinic activity and storm-track amplification maintained mainly by turbulent heat fluxes (both latent and sensible). In support of this mechanism, we also refer to our earlier study (Omrani et al., 2019), where we analyzed in detail the tropospheric storm-track, baroclinic eddy, and eddy-driven jet responses to midlatitude SST fronts using the same model configuration of SST-fronts. That work demonstrated that SST fronts strengthen baroclinicity and baroclinic wave activity, thereby intensifying the eddy-driven jet and the associated north Pacific low geopotential height anomalies (through geostrophic balance) —providing a coherent link between oceanic fronts, tropospheric circulation, and the enhanced upward planetary-wave flux that drives the stratospheric response.

In the North Atlantic sector, the precursory circulation pattern associated with weak vortex states and stratospheric warmings is typically characterized by positive (blocking like) geopotential height anomalies centered over the northern Euro-Atlantic region or Scandinavia (e.g. Nishii Nakamura et al. 2009). Such blocking-like high pressure events promote enhanced upward planetary-wave propagation into the stratosphere. In contrast to typical Atlantic precursors for weak stratospheric vortex, the response to the Atlantic SST-front in our experiments exhibits a positive NAO-like pattern (R1Fig. 5, the same figure presented as SFig. 9), with relatively lower pressure over the northern Euro-Atlantic and Scandinavian regions. This circulation configuration suppresses the development of high latitude blocking like high pressure and thereby inhibits upward wave propagation into the stratosphere as shown in R1Fig. 4 or SFig. 8.

Regarding the effect Gravity wave, it is included in the residuum. Which is dominated by the gravity waves in the middle atmosphere ((Lubis, Omrani et al. 2016). Unfortunately, we didn't output the different parametrized gravity wave drags from our model simulations. However, the residuum X in the TEM that we analyzed in detail is usely used to diagnose the GW in the

stratosphere and mesosphere (e.g. Lubis, Omrani et al. 2016). The orographic gravity waves dominate the residuum of TEM in the lower to middle stratosphere (below about 30hPa) and in the upper stratosphere and mesosphere (above 30 hPa) the rsiduum is dominated by the non-orographic gravity waves. We agree, all these waves are connected to the troposphere. The non-orographic gravity waves in the upper stratosphere and mesosphere are also originated from the troposphere, and their upward propagation is highly controlled by the response of the lower and middle stratosphere to planetary waves that are also provided by the troposphere. In general, if the background wind strengthens (weaken) by less (more) planetary wave propagation from the troposphere, more westerly (easterly) gravity wave drag provided from troposphere are filtered and therefore more easterly (westerly) gravity wave drag reach the upper troposphere and mesosphere leading to the reversing of the residual circulation and adiabatic temperature change and that is exactly what we get in our experiments.

Changes made in response to these comments:

We now clarify this link explicitly in the revised **Discussion** and **Methods** sections and include new figures in the Supplementary Material showing the full EP flux structure from surface to stratosphere as well as tropospheric change associated with the upwards wave propagation. In particular:
We added text and SFig. 8–9 explaining why the Pacific SST front dominates the stratospheric response. By extending the EP-flux diagnostics into the troposphere, we show that only the Pacific SST-front experiment produces enhanced upward planetary-wave propagation. This response is linked to strengthened tropospheric low-geopotential-height anomalies over the North Pacific, a well-established tropospheric precursor to weak vortex states and SSWs and is dynamically consistent with the SST-front-induced intensification of the Pacific storm track and eddy-driven jet (Lines 269–281; SFig. 8–9).

We also clarified that, unlike the Pacific, the Atlantic SST front generates a tropospheric response resembling a positive NAO-like pattern that lacks the high-latitude Euro-Atlantic blocking-like anomalies known to precede weak vortex and SSW events. Consequently, the Atlantic SST front does not favor strong upward planetary-wave propagation and produces only a weak stratospheric response, underscoring the dominant role of the Pacific sector in driving the hemispheric asymmetry (Lines 283–289; SFig. 8-9).

In addition, we clarified the physical interpretation of the residual momentum forcing term ($\bar{X}$) emphasizing that it represents unresolved momentum forcing primarily associated with gravity wave drag in atrtosphere and mesosphere. We explained how the vertical structure of $\bar{X}$ reflects known gravity-wave filtering behavior, with orographic gravity wave drag dominating the lower and middle stratosphere and non-orographic gravity waves becoming more important in the upper stratosphere and mesosphere. We also added that although individual gravity-wave components were not explicitly output, the diagnosed structure of $\bar{X}$ is dynamically consistent with established theory and previous studies (Lines 489–494).
We also added how the gravity waves and non-gravity waves were parametrized (in response to R2), which maks it clearer the tropospheric origine and cotrolling factors of these waves (Lines 495-503)

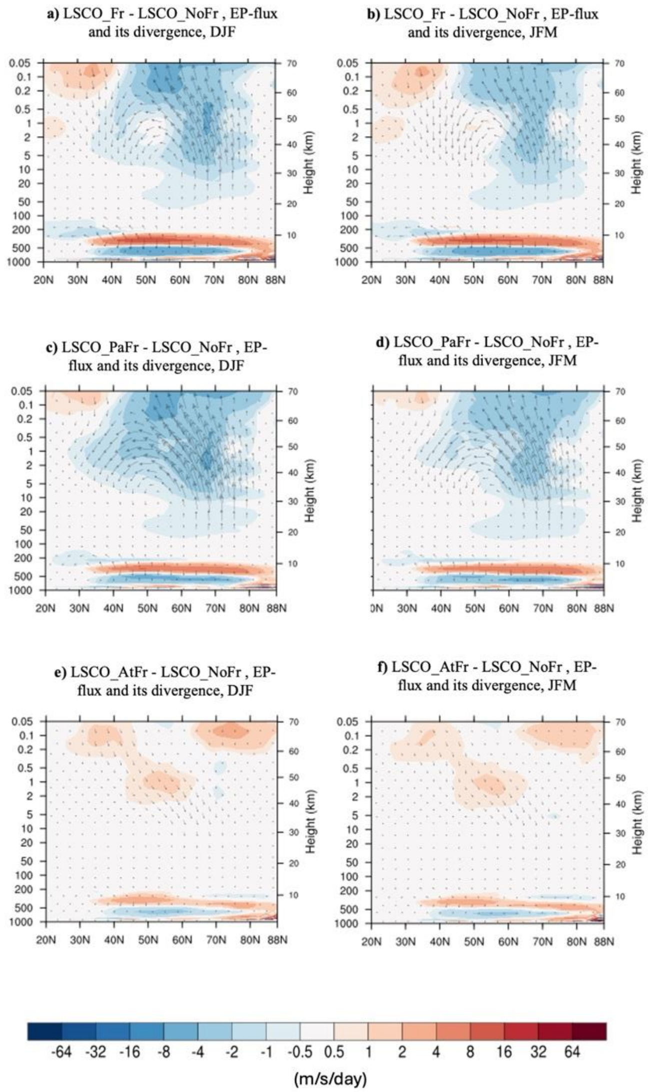

R1Fig. 4: EP-flux response to SST fronts and its regional components: Zonal-mean response of the Eliassen–Palm (EP) flux and its divergence to SST fronts. Panels (a–b) show the overall response to the inclusion of SST fronts (LSCO_Fr – LSCO_NoFr) for DJF and JFM, respectively. Panels (c–d) and (e–f) show the corresponding responses to the Pacific (PaFr – NoFr) and Atlantic (AfFr – NoFr) SST-fronts. Shading denotes the anomalous EP-flux divergence (m s^{-1} day^{-1}), and arrows indicate the scaled EP-flux vectors. Blue (red) shading indicates enhanced (reduced) upward and poleward wave propagation. The analysis extended into the troposphere reveals that the Pacific SST-front experiment produces the strongest upward propagation from the troposphere into the stratosphere, linking tropospheric storm-track intensification with enhanced stratospheric planetary-wave activity.

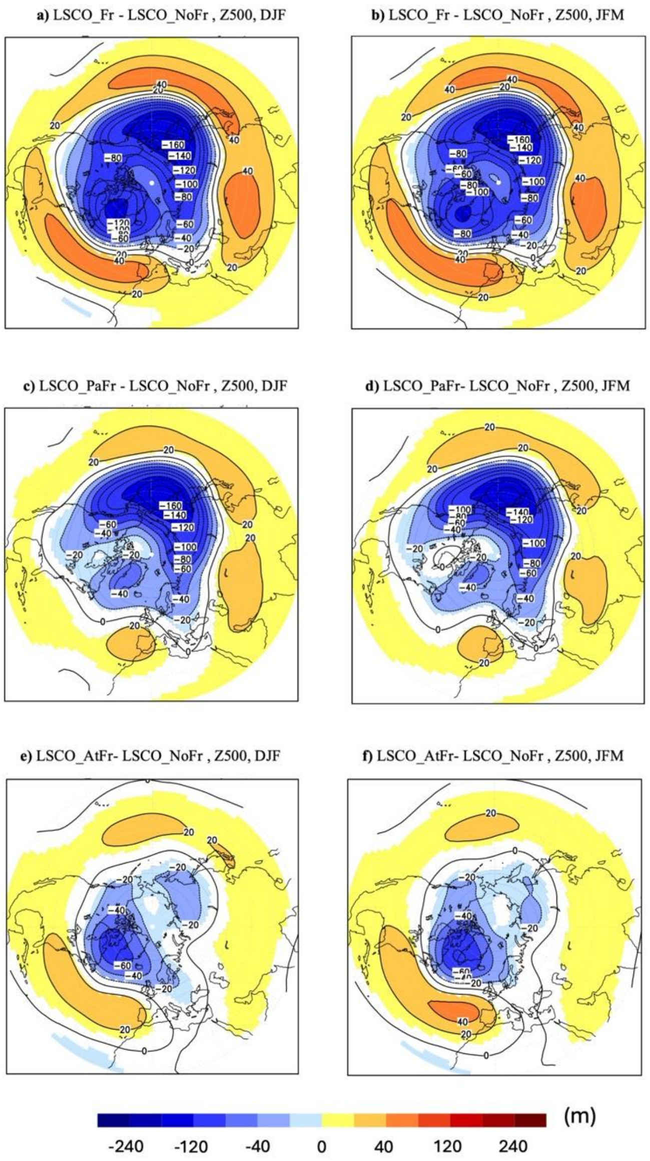

R1Fig. 5: Z500 response to SST fronts and their regional components: Tropospheric circulation response to SST fronts, shown as 500 hPa geopotential height anomalies (m). Panels (a–b) display the overall response to SST fronts (LSCO_Fr – LSCO_NoFr) for DJF and JFM, while panels (c–d) and (e–f) show the Pacific (PaFr – NoFr) and Atlantic (AfFr – NoFr) SST-front responses. Only statistically significant responses at the 95% confidence level (two-tailed Student's *t*-test) are shaded. Blue (yellow/red) shading indicates negative (positive) geopotential height anomalies. The Pacific SST-front response features a strengthened Aleutian Low, consistent with enhanced upward planetary-wave propagation and more frequent SSWs, while the Atlantic response exhibits an NAO-like pattern that does not favor strong upward wave activity.

3. The whole paper is based on an aqua-planet model setup without any observational evidence,

which is fine for a specialized paper in a specialized journal trying to un-derstand just a specific small piece of knowledge but is not enough for Nature Communications. More observational studies should be performed to justify the findings based on the model. For example, the authors could regress the BDC, PPSC, and SSW events in observational (e.g., SABER) or reanalysis dataset (e.g., ERA5 instead of NCEP for higher resolution) against SST fronts to show that SSW event frequency covaries with frontal strength and verify the mechanism in terms of the EP flux and others. The paper did include some basic results using the NCEP dataset in the supplementary (again, I cannot see the supplementary), but those are only for describing the phenomenon and no mechanism analysis is made thereby.

We thank the reviewer. First, we sincerely regret that the Supplementary information was accessible with the wrong name during the review process. It includes observational and reanalysis-based diagnostics from NCEP, which illustrate the strong hemispheric contrast in SSW frequency and PSC occurrence. These analyses are meant to highlight the observed asymmetry that motivates our modeling framework.

We would like to clarify that our study is not based solely on aquaplanet simulations. In addition to the aquaplanet experiments (AP_NoFr and AP_Fr), which are designed to represent Southern Hemisphere–like conditions, we conducted a complementary set of non-aquaplanet experiments that include realistic land–sea thermal contrasts and orography (LSCO_NoFr and LSCO_Fr) to emulate Northern Hemisphere–like configurations. These experiments are essential for testing how LSCO and SST fronts interact to shape stratospheric variability.

As discussed previously and in the manuscript, the primary objective of this study is not to analyze interannual variability or individual events, but to identify which large-scale boundary conditions — specifically, the presence or absence of SST fronts (associated with large-scale convergence zone of subpolar and subtropical gyre) and LSCO — explain the *systematic hemispheric asymmetry* in the frequency of SSWs and PSCs. This asymmetry is very strong and a robust feature of both observations and stratosphere-resolving models (SFig 2-3 and 4-7), and it arises from the permanent existence of the LSCO and SST fronts rather than year-to-year fluctuations of SST-fronts. For this reason, the mean-state and permanent existence of the SST fronts is far more relevant to our aim than their interannual variability. Regarding observational or ranalysis comparison, we have already analyzed the SH–NH asymmetry in SSW and PPSC frequency in the model and reanalysis data. These results were included in the Supplementary Material (submitted under a different filename by mistake) and confirm that the model reproduces the observed hemispheric contrast in both SSW and PPSC occurrence.

We acknowledge that observational regression analyses (e.g., using ERA5) could provide complementary support by illustrating how interannual variations in SST-front strength covary with stratospheric variability. Indeed, preliminary analyses that we performed using ERA5 show a Pacific response pattern consistent with our model experiments (see R1Fig. 6 compared to R1Fig. 7 below)— namely, low geopotential height anomalies over the North Pacific acting as a precursor for a weakened polar vortex during periods of enhanced Kuroshio SST-front intensity, which is consistent with our model response, where the Pacific SST-fronts dominate the response with

tropospheric low geopotential hight acting also as a precursor. However, we prefer to not include these results in the current paper for two reasons:

(1) they do not directly address the hemispheric asymmetry in the *mean-state* dynamics that this study focuses on, and

(2) A detailed dynamical assessment and multi-model evaluation, including high-resolution and eddy-resolving coupled models, are planned as part of an upcoming **NextGEMS-based study we are involved in**. We expect this analysis to further clarify how SST fronts enhance lower-tropospheric baroclinicity and storm-track activity, intensify the eddy-driven jet (as shown in Omrani et al. 2019), and—through geostrophic balance—amplify low geopotential height anomalies over the North Pacific, thereby increasing upward planetary-wave propagation and weakening the polar vortex, consistent with our model experiments. This work forms part of our **NextGEMS project deliverable** dedicated to assessing the added value of ultra–high-resolution coupled simulations in representing mesoscale air–sea interactions and their impact on large-scale atmospheric variability.

Change made in the paper

We clarified that our study targets the role of permanent, hemispheric-scale lower boundary conditions—land–sea contrast, orography, and midlatitude SST fronts—in setting the *mean* NH–SH asymmetry in SSW and PSC frequency. We emphasized that although interannual SST gradient variability may modulate stratospheric dynamics on a year-to-year basis, it is too weak to explain the robust hemispheric contrast, which persists despite interannual ocean variability. We also note that the role of interannual SST variability in stratosphere–troposphere coupling is an important topic for future work (Lines 330–338).

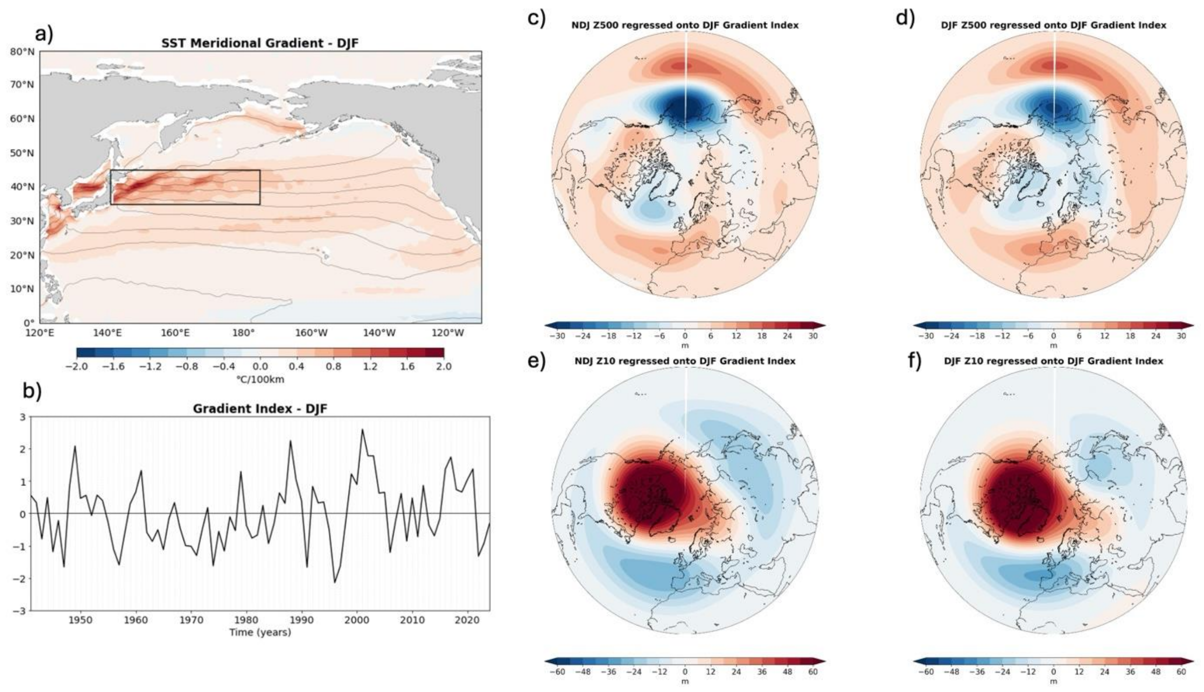

R1Fig. 6: Observed SST-gradient index in DJF and its tropospheric–stratospheric response in ERA5. (a) Climatology of the DJF meridional SST gradient (°C per 100 km), with the black box indicating the Kuroshio frontal region used to define the SST-gradient index. (b) Time series of the

standardized DJF Kuroshio SST-gradient index averaged over the marked region. (c–d) 500 hPa geopotential-height anomalies regressed onto the Kuroshio SST-gradient index for (c) NDJ and (d) DJF. (e–f) 10 hPa geopotential-height anomalies regressed onto the same index for (e) NDJ and (f) DJF. Blue (red) shading indicates negative (positive) height anomalies. Strengthened SST-gradients are associated with enhanced low geopotential-height anomalies over the North Pacific and a weakened polar vortex aloft—consistent with the Pacific SST-front mechanism identified in our model experiments (see next figure).

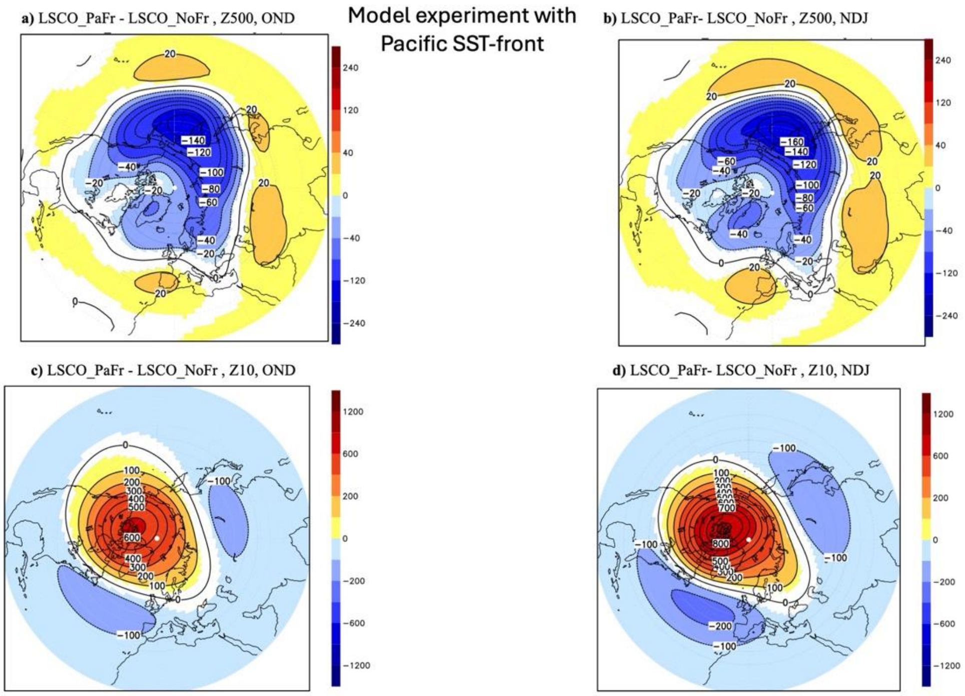

R1Fig. 7: **Model experiment with Pacific SST-front: tropospheric and stratospheric response:** (a–b) 500 hPa geopotential-height anomalies (Z500, m) and (c–d) 10 hPa geopotential-height anomalies (Z10, m) for OND (a, c) and NDJ (b, d), showing the difference between the experiments LSCO_PaFr and LSCO_NoFr. Blue (red/yellow) shading indicates negative (positive) height anomalies. The Pacific SST-front induces pronounced low-height anomalies over the North Pacific, consistent with enhanced Aleutian-Low-like circulation and increased upward planetary-wave propagation. This tropospheric response leads to weakened polar-vortex conditions aloft, in agreement with the mechanism linking SST-fronts to stratospheric variability. Only anomalies significant at the 95 % confidence level (two-sided *t*-test) are shaded.

4. Some details about the experimental design and the analysis framework are not clear. 1) The captions of Fig. 1 and 2 claim that they show the seasonal evolution of westerly wind and PPSC, but why is the x-axis from November to May, covering only 6 months? 2) I thought SSW events primarily occur in NH winter, but why in Fig. 1 the zonal wind sign reversal happens more in spring. How could this happen? Or is there a mistake in the labels of the x-axis? 3) I understand the logic of the pa-per is that the EP flux divergence associated with LSCO and fronts drives reduction to the background westerly jet around the year, instead of their effects on individual events. Then, what is shown in Fig. 3-5? 62-year annual mean? Or winter mean? It should be

made clear. 4) Is the model run continuously for 62 years forced by Had-ISST, or is it run for 62 separate periods spanning Nov-May? If the latter case (which I think is better expect for the seasons), how is the model initialized for each run? 5) How is the effects of land-sea thermal contrast and orography introduced into the SST? This is not described in the "Methods" section or anywhere in the paper. Is it shown in the supplementary? I have difficulties imaging adding the effect of orography just in SST. The land-sea thermal contrast could, I guess, probably be introduced by setting the SST to some typical land temperature over land. This must be clarified.

We thank the reviewer for pointing these points.

Response to Comment 4.1: Time Axis in Figs. 1 and 2

In this study, we focus primarily on the extended winter season, when SSWs and PSCs occur. We therefore limited the time axis in Figs. 1 and 2 to November through April, which captures the full evolution of the polar vortex and PSC activity from onset to breakdown. This 6-month window is centered on the climatological winter maximum and excludes months when polar stratospheric variability is minimal and stratospheric wind is easterly.

Response to the comment in the paper:

To clarify, we have updated the figure captions and text to refer to the **"The November to April"** rather than "seasonal evolution."

Response to Comment 4.2:

I thought SSW events primarily occur in NH winter, but why in Fig. 1 the zonal wind sign reversal happens more in spring. How could this happen?

Spring Wind Reversals and Final Warmings:

We thank the reviewer for this observation. The apparent increase in zonal wind reversals during spring in Fig. 1 reflects mostly the occurrence of final warmings, not dynamically driven Sudden Stratospheric Warmings (SSWs). This final warming and associated easterly wind persist all along the spring and summer and are not accounted as SSW. In middle atmosphere (stratosphere and mesosphere), the wind is westerly during the extended winter season (including large part of autumn and small part of spring) and easterly during the extended summer season (including large part of the spring and small part of autumn see below for details) (See Andrew and Holton book 1986, Fig. 4 in Ch 1).

In general, the polar stratospheric circulation is dominated by strong westerly winds during winter and early spring, which by thermal wind balance results from the polar middle atmospheric radiative cooling due to the absence of sunlight. During this time, any reversal of the westerlies—if followed by a return to westerly flow—is typically dynamically driven by upward-propagating planetary waves and is classified as a Majore Sudden Stratospheric Warming (SSW).

As spring progresses, solar radiation returns to the polar region, and UV absorption by ozone warms the upper stratosphere. This leads to a radiatively driven breakdown of the polar vortex, resulting in a persistent reversal to easterlies. The onset of this seasonal, radiatively induced warming is what is known as final warming.

Final warmings are characterized by a permanent transition of the stratospheric zonal winds from westerlies to easterlies, occurring in spring, and are not followed by a return to westerlies. These are distinct from dynamically forced SSWs and mark the seasonal end of the background winter

stratospheric westerlies and associated radiative cooling and return to easterly background wind and associated UV-absorption by ozone. The exact date of the occurrence of the finale warming varies from year to year.

In our analysis, we apply standard SSW detection criteria that exclude final warmings from the SSW count. This exclusion is implemented by identifying as SSWs only those wind reversals that occur during the radiatively cooled season and are followed by a return to westerlies and stratospheric cooling. As a result, much of the easterly flow that appears in spring is not classified as SSW but instead reflects final warmings—radiatively driven transitions that do not return to westerly flow or a cooled polar stratosphere.

Response to the comment in the paper:
We have now clarified this in the Methods section with the following sentence in Lines 450-452: "Only zonal wind reversals that return to westerlies are counted as SSWs. Persistent springtime reversals that do not return to westerlies are classified as final warmings and are excluded from the SSW count"

Response to Comment 4.3: Temporal Averaging in Figs. 3–5

Figures 3–5 show climatological winter (JFM) means, averaged over all simulation years. We chose this period because it coincides with the peak SSW activity and the strongest stratospheric circulation anomalies in the model. These are not annual means.

Response to the comment in the paper:
We have now clarified this in the figure captions.

Response to Comment 4.4: Simulation Length and Continuity
The model was run continuously for 62 years, using monthly climatological SSTs from HadISST1 repeated annually. Each integration includes a full seasonal cycle, allowing the model to simulate spontaneous stratospheric variability, including vortex breakdowns and SSWs.

Response in the paper:
We have added the following sentence to the Methods section in lines Lines 426-428:
"Each experiment is integrated continuously for 62 years using a repeating annual cycle of monthly climatological SSTs derived from HadISST1."

Response to Comment 4.5: How LSCO Is Introduced

We thank the reviewer for raising this clarification. The effect of land–sea thermal contrast and orography (LSCO) is not introduced via modifications to the SST field. Instead, it is implemented by restoring the model's land–sea mask and topography.

To isolate the effect of LSCO, we compared two experiments:

- AP_NoFr: aquaplanet configuration with zonally averaged SSTs without SST-fronts and no continents or orography (see Sfig. 1 and Omrani et al. 2019 for the way of removing the SST-front).
- LSCO_NoFr: identical SST field (no front), but with realistic continents and topography restored. We just put the realistic land–sea mask and topography in the aquaplanet configuration.

Comparing the aquaplaned experiment AP_NoFr withot LSCO and the experiment with realistic LSCO with the same non-front configuration isolate the effect of LSCO alone.

Response in the paper:

We clarified this in the Methods section in L415-417:

5. The model did not have sea ice, which could be a big problem. The authors argued that they removed sea ice on purpose to isolate the effects of SST fronts. But I think this is unreasonable because you just have to keep the sea ice boundary condition identical in the four experiments to achieve that. As long as the only thing different in the experiments is the SST front and/or the LSCO, you don’t have to worry about mixing different effects in the results. On the other hand, the most serious problem is regarding the model’s basic state and thermodynamic mechanisms responding to the midlatitude SST fronts. The absence of sea ice could force excessive upward heat flux at the surface, and cause erroneous low-level warming, weakened polar vortex, and increased SSW frequency. The propagation of planetary waves is affected too, which is essential to the BDC response. See Peings & Magnusdottir (2014, JC) and Kim et al. (2014, GRL) for the effect of sea ice. If you really want to keep the cur-rent simulation results, at least verify your model basic state.

Response to Comment 5: Sea Ice Exclusion

We thank the reviewer for this important point and fully agree that sea ice is a key component of the high-latitude climate system. In this work we wanted to focus only on the impact of SST-front excluding the effect of sea-ice. The reason we chose to remove sea ice in all experiments is not to ignore its importance, but to preserve the experimental control needed to isolate the effects of SST fronts. This can be seen clearer when looking on the SST-gradient in the experiment setup shown in supplement. Which was missing in the previous submission. Specifically, our no-front experiments use a zonally symmetric SST distribution with a linear poleward decreas with constant gradient as slop (Sfig. 1a). If sea ice were retained on top of this smoothed SST field, it would introduce an abrupt SST discontinuity at the ice edge, resulting in an artificial front that could spuriously generate atmospheric responses and compete with or obscure the intended frontal signal.

This issue does not arise from sea ice itself, but from the conflict between how sea ice is prescribed and how we remove the oceanic SST front. To maintain a clean comparison and fully isolate the influence of midlatitude SST fronts, we therefore removed sea ice uniformly across all experiments. This method has been adopted form different previous studies that aim to isolate frontal effects without interference from polar discontinuities (Ogawa eat al. 2012, Ogawa et.al. 2015, Omrani et al. 2019, Sampe et al. 2013 and Sampe et al. 2010)

To address this concern, we now have an additional experiment (LSCO_NHclim) that includes realistic Arctic sea ice climatology and realistic zonal SST, and the same midlatitude SST fronts applied in LSCO_Fr. This experiment enables the study of the effect of the missing Arctic sea ice component in our SST-fronts experiments. R1Fig.8-10 show a comparison of the response of this experiment with our SST-front only experiment for temperature, wind. EP-flux divergence and different forcing with associated residual circulation for DJF and JFM. Comparing LSCO_NHclim – LSCO_NoFr with LSCO_Fr – LSCO_NoFr shows that adding sea ice does not significantly change the sign, spatial structure, or dynamical mechanism of the response. The strengthened upward planetary-wave propagation, the weakened polar vortex, the warming (cooling) anomalies in the stratosphere (uppermost stratosphere and mesosphere), and the EP-flux structure remain very similar in both experiments. Thus, the new experiment demonstrates that the midlatitude SST fronts—not the absence of sea ice—constitute the primary driver of the stratospheric response in our study and therefore the mean oceanic driver of the stratospheric asymmetry in the stratospheric dynamic between NH and SH.

Changes made in the paper inresponse to these comments:

We clarified that sea ice was removed in all experiments to avoid artificial SST gradients at the ice edge in the no-front configurations. We explained that the zonally symmetric SST smoothing used to remove SST fronts would otherwise create artificial discontinuities when intersecting fixed sea-ice boundaries, interfering with the intended isolation of SST-front effects (Lines 396-401).

We added Supplementary Figure SFig. 10 to show that the stratospheric circulation response to combined SST-front and Arctic sea-ice forcing does not differ significantly from the response to SST fronts alone. This demonstrates that, in our experiments, SST fronts exert a dominant influence on the stratospheric circulation compared to Arctic sea-ice effects (Lines 354–357).

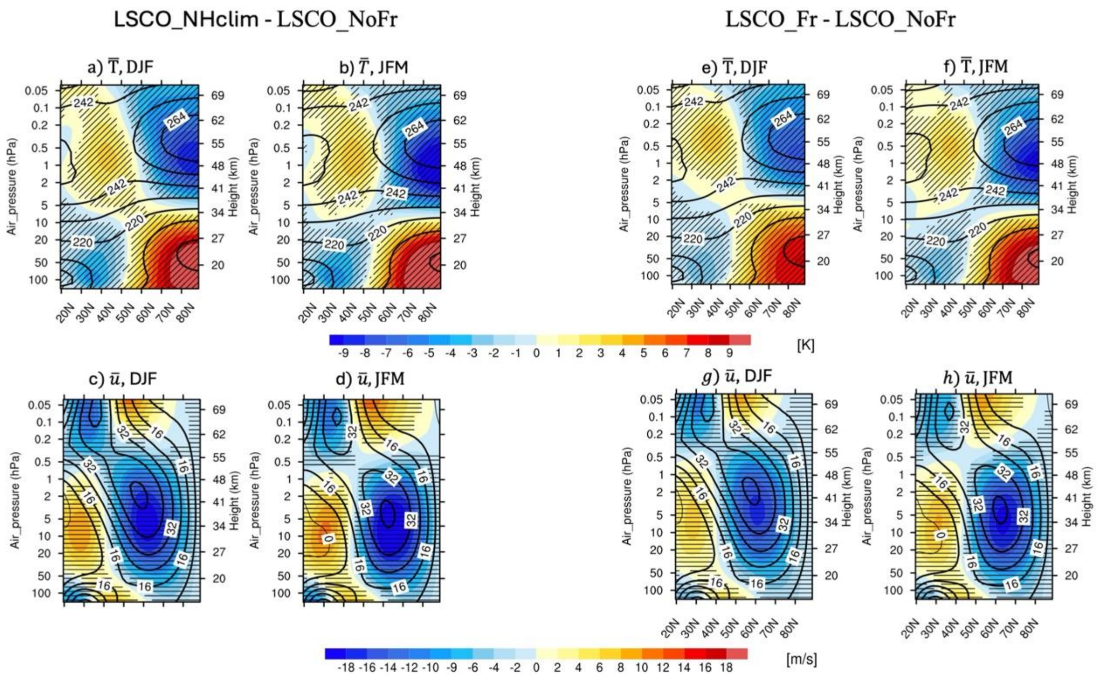

R1Fig. 8. Zonal-mean temperature and zonal-wind responses to midlatitude SST fronts and to the combined SST-front–Arctic-sea-ice forcing: Panels (a–d) show the response when realistic Arctic sea ice is added to the SST-fronts (LSCO_NHclim – LSCO_NoFr), while panels (e-h) show the response to midlatitude SST fronts alone (LSCO_Fr – LSCO_NoFr). The top row (a–b, e-f) displays zonal-mean temperature anomalies (K) for DJF and JFM, and the bottom row (c-d, g-h) shows zonal-mean zonal-wind anomalies (m s^{-1}) for the same seasons. Shading denotes anomaly amplitude; stippling marks regions significant at the 95% confidence level (Student's t-test). Both experiments exhibit a robust warming of the stratosphere up to ~5 hPa, associated with enhanced upward wave propagation (R1Fig. 9), and a cooling in the uppermost stratosphere and lower mesosphere, consistent with a weakened residual circulation.

The zonal-wind response shows a weakening of the polar-night jet accompanied by strengthened midlatitude westerlies. The close similarity between the response of LSCO_NHclim and LSCO_Fr indicates that midlatitude SST fronts constitute the dominant driver of the stratospheric dynamical response, while the additional effects of Arctic sea ice in LSCO_NHclim modify but do not fundamentally change the mechanism (see R1Fig. 9-10 below).

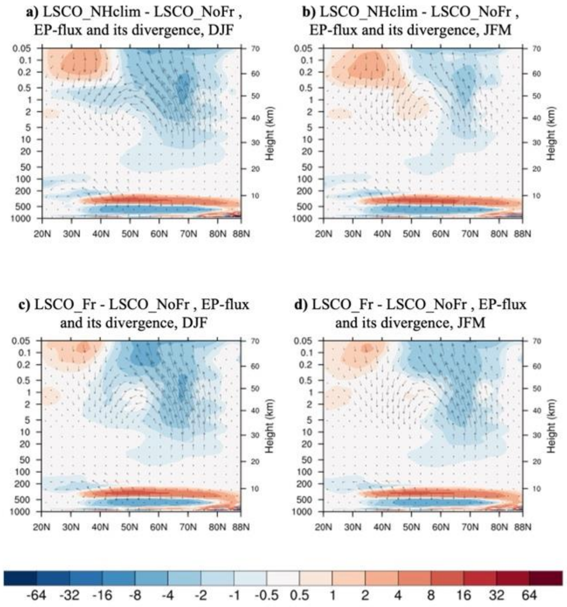

R1Fig. 9: Zonal-mean EP-flux response to (left) combined SST-front–Arcitic-sea-ice forcing and (right) SST-front forcing only: Panels (a–b) show the Eliassen–Palm (EP) flux anomalies and their divergence for LSCO_NHclim – LSCO_NoFr, which includes midlatitude SST fronts together with realistic Arctic sea ice. Panels (c–d) show the EP-flux response to midlatitude SST fronts alone (LSCO_Fr – LSCO_NoFr). Results are shown for DJF (left column) and JFM (right column). Shading denotes EP-flux divergence (m s^{-1} day^{-1}), with blue indicating EP-flux convergence and red indicating WP-flux divergence. Vectors show the EP-flux (horizontal and vertical components scaled for visual clarity). Both experiments exhibit a strong enhancement of upward planetary-wave propagation from the lower atmospheric levels into the stratosphere, centered around 50°–60° N, together with increased EP-flux divergence in the polar stratosphere, consistent with a weakened polar vortex. The close agreement between LSCO_NHclim and LSCO_Fr demonstrates that midlatitude SST fronts are the primary driver of the enhanced wave forcing, while sea ice modulate but do not fundamentally change the mechanism.

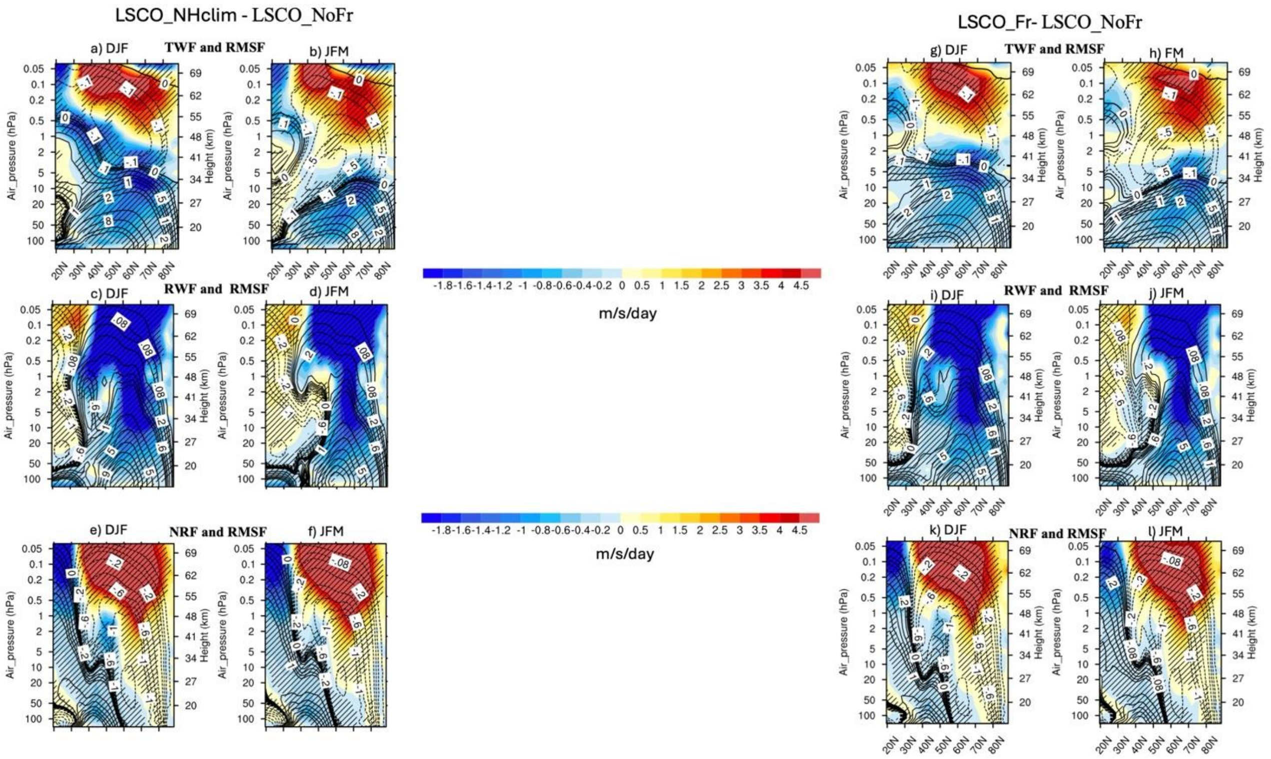

R1Fig. 10. Decomposition of TEM momentum forcing with comparison between combined SST-front and Arctic sea ice forcing (LSCO_NHclim) and SST-front forcing alone (LSCO_Fr): Panels (a–f) show the response to the combined SST-front + sea-ice forcing (LSCO_NHclim – LSCO_NoFr) for DJF and JFM. Panels (g–l) show the response to midlatitude SST fronts alone (LSCO_Fr – LSCO_NoFr). Each row isolates a different contribution to the Transformed Eulerian Mean (TEM) momentum equation: (a–b, g–h): Total Wave Forcing (TWF) and associated residual mean streamfunction (RMSF), (c–d, i–j): Resolved Wave Forcing (RWF) and associated RMSF, (e–f, k–l): Non resolved wave or "Residual" Forcing (NRWF) — dominated by parameterized gravity-wave drag — and associated RMSF. Shading indicates forcing anomalies (m s^{-1} day^{-1}). Black contours show the residual mean meridional streamfunction. Stippling marks regions significant at the 95% level (Student's t-test).
Across all diagnostics, the LSCO_NHclim response closely resembles the LSCO_Fr response, with only small differences due to the inclusion of sea ice and zonal tropical SST asymmetry. These results show that midlatitude SST fronts are the primary driver of the stratospheric dynamical response. Arctic Sea ice slightly modulate the amplitude but do not alter the mechanism.

6. The reason why the Pacific SST front is more effective should be discussed in more details. Is this front the Kuroshio-Oyashio Extension (KOE) front studied in many literature works? Is the Atlantic front the Gulf Stream (GS) front? Why are they dif-ferent? Is it because the KOE front is stronger, or is it because the Pacific is wider and its storm track is more powerful? As far as I know, this is somehow inconsistent with the findings of Kim et al. (2014) that the intensity of Atlantic storm tracks de-termines the frequency of SSW. Since the GS front modulates the Atlantic storm track, I would assume it has more impacts on the SSW too.

Response to Comment 6: Why the Pacific SST Front Is More Effective

We thank the reviewer for raising this question. Our experiments show that the Pacific SST front (representing the Kuroshio–Oyashio Extension, KOE) produces a substantially stronger stratospheric response than the Atlantic SST front (Gulf Stream Extension). According to previous studies on the tropoapheric precussore of stratospheric changes, we know that the stratosphere is dynamically sensitive to well defined specific pattern that acts to change the upward wave propagation of tropospheric wave into the stratosphere. Therefore, the differences in our experiments will depend on how each SST-front will simulate the well known tropospheric precursors. As described in our response to Comment 2 and shown in the revised Supplementary Figures (SFig. 8–9), extending the zonal-mean EP-flux diagnostics downward into the troposphere reveals that only the Pacific SST-front experiment exhibits a clear strengthening of upward planetary-wave activity entering the stratosphere. To understand the tropospheric origin of this enhanced wave forcing, we examined the 500 hPa geopotential height response.

The Pacific SST front generates **enhanced low geopotential height anomalies in the North Pacific**, which has been shown as efficient precursor for weak vortex events and therefore also for SSWs (e.g., Garfinkel and Hartmann 2008, Ineson and Scaife 2009, Nishii, Nakamura et al. 2009, Garfinkel, Butler et al. 2012). This tropospheric precursory circulation pattern is dynamically consistent (through geostrophic balance) with the strengthened storm-track and eddy-driven jet response to Pacific SST fronts found in Omrani et al. (2019).

In contrast, the Atlantic SST-front experiment produces a **positive NAO-like response** (see Sfig. 9) with **no high-latitude Euro-Atlantic or Scandinavian ridge**, known as Atlantic sector precursor of weak vortex and stratospheric warming (e.g. Nishii & Nakamura 2010 see response to comment 2). This explains why the Atlantic SST front does not produce enhanced upward EP-flux and thus has little effect on the stratospheric vortex.

Accordingly, the stronger stratospheric impact of the Pacific SST front arises because **only the Pacific front generates the correct tropospheric precursor pattern** known to increase upward planetary-wave propagation in the zonal mean. We now clarify this interpretation in the revised Discussion and explicitly refer to the supporting figures and right references.

Changes made in response to this comment:

We note that the key aspects of this comment are already addressed directly in the manuscript through the revisions made in response to Comment 2, where:

we added text and SFig. 8–9 explaining why the Pacific SST front dominates the stratospheric response. By extending the EP-flux diagnostics into the troposphere, we show that only the Pacific SST-front experiment produces enhanced upward planetary-wave propagation. This response is linked to strengthened low-geopotential-height anomalies over the North Pacific, a well-established tropospheric precursor to weak vortex states and SSWs and is dynamically consistent with the SST-front-induced intensification of the Pacific storm track and eddy-driven jet (Lines 269–281; SFig. 8–9).

we also clarified that, unlike the Pacific, the Atlantic SST front generates a tropospheric response resembling a positive NAO-like pattern that lacks the high-latitude Euro-Atlantic blocking-like anomalies known to precede weak vortex and SSW events. Consequently, the Atlantic SST front does not favor strong upward planetary-wave propagation and produces only a weak stratospheric response, underscoring the dominant role of the Pacific sector in driving the hemispheric asymmetry (Lines 283–289; SFig. 8-9).

Minor comments:

1. The paper defines SSW events purely with wind reversals without considering reversals of meridional temperature gradient. Strictly speaking, this is not SSW events defined by

WMO which requires both wind and temperature.

In this study, we detect SSW events using the method of Charlton and Polvani (2007), which is widely adopted in the stratospheric research community. This approach defines major SSWs based on the reversal of the zonal-mean zonal wind at 10 hPa and 60°N from westerly to easterly. Although the meridional temperature gradient criterion used in the full WMO definition is not explicitly included, it is implicitly accounted for: under thermal wind balance, a reversal of zonal-mean westerly winds is necessarily associated with a reversal of the meridional temperature gradient below the altitude of maximum wind reversal.

Final warmings are excluded by requiring a return to westerlies following the wind reversal, ensuring that the event is dynamically driven and not part of the seasonal transition to summer easterlies.

We added in the method L448-449:

Although the inversion of the meridional temperature gradient criterion is not explicitly included, it is implicitly accounted for under thermal wind balance.

2. The paper uses words like “pivotal” and “critical” to describe the role of SST fronts, but does not give quantitative evidence supporting such strong conclusions. As said in the paper, the effect of SST fronts is much weaker than those driven by LSCO.

When we used terms such as “pivotal” or “critical” in reference to the SST front, we were referring specifically to its role in increasing SSW frequency and reducing PSC occurrence—particularly the shift from 1 SSW with no front to around 9 events per decade with fronts, which we consider a threshold-like dynamical transition. We fully agree that the overall stratospheric response to SST fronts is quantitatively weaker than that driven by LSCO in terms of zonal wind deceleration. In fact, LSCO is responsible for most of the weakening of westerlies and the associated reduction in PSCs. However, the LSCO-only configuration is not sufficient to bring the SSW frequency close to observed NH values. We acknowledge that our earlier phrasing may have inadvertently

understated the importance of LSCO, and we have revised the wording throughout the manuscript to more accurately reflect the complementary roles of SST fronts and LSCO.

Changes in the papper:

In response to this comment, we revised key formulations throughout the manuscript to better reflect the relative roles of SST fronts and LSCO. Specifically, we replaced expressions like *"pivotal"* or *"critical"* with more precise terms such as *"critical complementary role"* or *"important supporting role"* when describing the influence of SST fronts. These revisions emphasize that SST fronts contribute an essential but secondary dynamical effect—particularly in enabling the transition to more NH-like SSW frequency and PSC conditions—while maintaining that LSCO remains the primary driver of the mean-state zonal wind weakening. These clarifications were made in the Abstract, Results, and Discussion, as well as in the introductory framing of the mechanism.

4. The Discussion section is just a lengthy repeat of the Results, with only a little bit discussion on implications and limitations in the last two paragraphs. Please shorten the review of results, extracting only the most saline findings, and add more discussions on important issues and comparison with previous studies.

Response to Minor Comment 4:

We agree with the reviewer that the original Discussion section was too long and included repetition of earlier results. Our intention was to provide the reader with a clear and self-contained summary of the mechanisms—particularly in relation to the final schematic diagram—which integrates and distills the core physical processes investigated in the paper. We wanted readers to come away with a coherent understanding of how LSCO and SST fronts interact to shape stratospheric dynamics.

That said, we acknowledge the reviewer's concern and have now shortened the summary of results, reducing overlap with the Results section and focusing more directly on the key findings, implications and additional aspects.

Changes made:

We have also added a new paragraph discussing aspects that were not covered in the main text, including broader implications and connections to prior studies. To reflect this refined structure, we now title the section **"Conclusion and Discussion."**

In addition, the revised discussion elaborates on the basin-specific effectiveness of SST fronts in triggering SSWs, the role of precursor circulation patterns (as response to major comments of R2), limitations of the experimental design (e.g., absence of sea ice), and future directions related to SST variability and stratospheric extremes.

5. The Method section is too verbose, including many sentences about background, purpose, or findings, that belong better to the introduction or results sections. Please in this section only describe the methods with concise language.

Thank you for this comment.

We have reduced the Methods section accordingly. However, because of the limited space in the main text, we retained some explanatory statements that justify our model setup, particularly in response to specific reviewer comments, as well as some details on the gravity-wave parameterization in the model and its interpretation. These additions are intended to clarify the experimental design and describe the model used, rather than to discuss results or interpretations.

6. Caption of Fig. 4e: due to

Done.

7. Line 144: details -> detailed
Done

8. Line 155: componeaffents? What's that?
It should be components RWF and NRF

Reference:

Andrews, D. G., J. R. Holton and C. B. Leovy (1987). Middle Atmosphere Dynamics, Academic Press.
Garfinkel, C. I., A. H. Butler, D. W. Waugh, M. M. Hurwitz and L. M. Polvani (2012). "Why might stratospheric sudden warmings occur with similar frequency in El Nino and La Nina winters?" Journal of Geophysical Research-Atmospheres 117: D19106.
Garfinkel, C. I. and D. L. Hartmann (2008). "Different ENSO teleconnections and their effects on the stratospheric polar vortex." Journal of Geophysical Research-Atmospheres 113(D18).
Ineson, S. and A. A. Scaife (2009). "The role of the stratosphere in the European climate response to El Nino." Nature Geoscience 2(1): 32-36.
Lubis, S. W., N. E. Omrani, K. Matthes and S. Wahl (2016). "Impact of the Antarctic Ozone Hole on the Vertical Coupling of the Stratosphere-Mesosphere-Lower Thermosphere System." Journal of the Atmospheric Sciences 73(6): 2509-2528.
McLandress, C. and T. G. Shepherd (2009). "Simulated Anthropogenic Changes in the Brewer-Dobson Circulation, Including Its Extension to High Latitudes." Journal of Climate 22(6): 1516-1540.
Nishii, K., H. Nakamura and T. Miyasaka (2009). "Modulations in the planetary wave field induced by upward-propagating Rossby wave packets prior to stratospheric sudden warming events: A case-study." Quarterly Journal of the Royal Meteorological Society 135(638): 39-52.
Ogawa, F., H. Nakamura, K. Nishii, T. Miyasaka and A. Kuwano-Yoshida (2012). "Dependence of the climatological axial latitudes of the tropospheric westerlies and storm tracks on the latitude of an extratropical oceanic front." Geophysical Research Letters 39.

Ogawa, F., N. E. Omrani, K. Nishii, H. Nakamura and N. Keenlyside (2015). "Ozone-induced climate change propped up by the Southern Hemisphere oceanic front." Geophysical Research Letters 42(22): 10056-10063.
Omrani, N.-E., F. Ogawa, H. Nakamura, N. Keenlyside, S. W. Lubis and K. Matthes (2019). "Key Role of the Ocean Western Boundary currents in shaping the Northern Hemisphere climate." Scientific Reports 9(1): 3014.

T. Sampe, H. Nakamura, A. Goto, Potential Influence of a Midlatitude Oceanic Frontal Zone on the Annular Variability in the Extratropical Atmosphere as Revealed by Aqua-Planet Experiments. *J Meteorol Soc Jpn* 91A, 243-267 (2013).
T. Sampe, H. Nakamura, A. Goto, W. Ohfuchi, Significance of a Midlatitude SST Frontal Zone in the Formation of a Storm Track and an Eddy-Driven Westerly Jet. *J*

Reviewer #2 (Remarks to the Author):

This is an interesting study showing the importance of oceanic fronts as drivers of stratospheric variability. The analysis is based on several idealized atmospheric-only experiments in which the effects of oceanic fronts is compared to the effect of land-sea contrast on stratospheric variability. The set of experiments include a pair of Aqua-Planet (AP) configurations and another pair with realistic orography (land-sea thermal contrast and orography, LSCO), both with and without oceanic fronts. Basin-specific contributions from the Pacific and the Atlantic are also assessed separately. Results highlight the overlook role of SST-fronts in driving stratospheric variability, and particularly in sudden stratospheric warming (SSW) occurrence and Polar Stratospheric Cloud formation, which appears to play a key role in the LSCO configuration. The observed impacts are explained via an acceleration of the residual mean circulation and therefore an adiabatic warming in the polar stratosphere.
The manuscript is overall well-written and the messages are quite clear. Particularly interesting is the focus on Polar Stratospheric Cloud formation in the Arctic stratosphere since this is a significant issue considering the uncertainties in the future trends of the Northern Stratospheric Polar vortex. However, there are several unclear points that need clarification before publication.

We thank Reviewer 2 for the careful reading of the manuscript and for the detailed and constructive comments. The reviewer's remarks helped improve the manuscript considerably. In particular, they motivated us to clarify the role of background wind conditions and wave–mean flow interaction in shaping the stratospheric response, to better explain the respective roles of resolved and parameterized wave forcing and their model dependence, and to strengthen the mechanistic interpretation linking SST fronts, tropospheric circulation changes, and SSW occurrence. The reviewer's comments also led to improvements in the presentation, terminology, and methodological clarity, thereby enhancing the overall rigor and readability of the manuscript.

I think too much attention is paid to separate the effects of resolved and non-resolved wave forcing. I understand this is a common procedure in this kind of analysis, but it should be acknowledged that this is quite model dependent, and very much depends on the particular

parametrization of gravity waves used. I specifically missed some comments on the parametrization used if the specific contribution of resolved vs. parametrized waves is considered to be so important.

Moreover, the conclusion was somehow expected, since by wave filtering, as you explain in the manuscript, non-resolved waves show a major role in the upper stratosphere and mesosphere, while resolved waves mainly force the stratospheric circulation. In fact, note that this result is not even mentioned in the abstract. I still think this analysis is correctly developed through the paper, and although it is valuable, it takes up too much space, and more priority should be given to explain the mechanisms leading to the results.

In particular, less attention is paid to the large changes in the background winds in the different configurations and how this may affect wave propagation. In principle, in the experiments, you are modifying wave generation: including or not those related to fronts in Fr/NoFr cases, and large-scale Rossby wave trains in the AP/LSCO configurations. In addition, upward wave propagation from the lower stratosphere is also modified via changes in the background flow: conditions may be very different in the Aqua Planet vs. the LSCO experiment; even we are not shown the different wind profiles, it can be inferred both Figure 1, or the already large differences in LSCO_NoFr and LSCO_Fr in Figures 3c and 3d. It would be helpful to quantify the impacts of LSCO and SST fronts on the background conditions to interpret the role that the increased front-related wave forcing has on SSWs. Are these waves just modifying the background conditions or are they directly responsible for triggering SSW? This is important because from Figure 1, one could interpret that they are responsible for SSW triggering – they have a major impact on the variability than on the mean wind value, but SSW occurrence is largely associated with resolved waves, how is this reconciled?

In this sense, line 12 reads "This warming significantly enhances Arctic SSW frequency and strongly reduces PSC formation."

From this statement, one could assume that SST fronts are responsible for background wind changes that favor conditions for SSWs, but, is this shown? As previously commented, Figure 1 shows that the largest effect on the mean flow is LSCO, and the effect of fronts mainly acts to increase the variability, which is more effective in producing SSWs because winds are already weaker: closer to zero (cf. Figure 1 left and right panels). Again, more details are needed in the specific role of fronts on SSW occurrence.

We added in the Method:

I think too much attention is paid to separate the effects of resolved and non-resolved wave forcing. I understand this is a common procedure in this kind of analysis, but it should be acknowledged that this is quite model dependent, and very much depends on the particular parametrization of gravity waves used. I specifically missed some comments on the parametrization used if the specific contribution of resolved vs. parametrized waves is considered to be so important:

We thank the reviewer for this insightful comment. We agree that the separation between resolved wave forcing (RWF) and non-resolved wave forcing (NRWF) is inherently model-dependent, particularly due to the parameterization schemes used for subgrid-scale gravity waves. Even with this critical model dependency, in general the inclusion of the stratosphere and associated

parameterization of non-orographic gravity wave improves the simulation of the stratospheric mean state and variability and its sensitivity to the SST (Cagnazzo and Manzini 2009, Karpechko and Manzini 2012, Omrani, Keenlyside et al. 2014). The stratosphereresolving MAECHAM5 used well-established parameterization schemes of **orographic gravity wave drag** (OGWD) and **Non-orographic gravity wave drag (NOGWD).** The OGWD is treated following the **McFarlane (1987)** scheme, which depends on near-surface winds and topography. NOGFD is parameterized using the **Hines (1997)** spectral formulation, which launches a broad spectrum of waves from the troposphere and simulates vertical momentum deposition based on critical level filtering and saturation processes. Whereas the OGWD-parametrization is required for all model even in low top models such as low top ECAHM5 (Omrani et. al. 2014), the NOGWD parametrization is found mainly in the stratosphere resolving high top models. The parametrizations of the NOGWD and the inclusion of the upper stratosphere are essential in simulating a realistic stratosphere. Low top model without NOGWD tends to lack the stratospheric variability and the right sensitivity of the stratosphere to forcing like SST (Omrani et al. 2014, Cagnazzo and Manzini 2009; Karpechko and Manzini 2012).

Changes in the paper:
We clarified the gravity wave drag parameterizations used in MAECHAM5 by explicitly describing the orographic (McFarlane 1987) and non-orographic (Hines 1997) schemes and their respective roles in shaping stratospheric circulation. We also added a statement emphasizing that the separation between resolved and non-resolved wave forcing is model dependent and may affect quantitative results, while noting that inclusion of non-orographic gravity wave drag is essential for a realistic simulation of stratospheric variability and its response to SST forcing (Lines 495–502).

Moreover, the conclusion was somehow expected, since by wave filtering, as you explain in the manuscript, non-resolved waves show a major role in the upper stratosphere and mesosphere, while resolved waves mainly force the stratospheric circulation. In fact, note that this result is not even mentioned in the abstract.

I still think this analysis is correctly developed through the paper, and although it is valuable, it takes up too much space, and more priority should be given to explain the mechanisms leading to the results.

We thank the reviewer for this helpful observation. We agree that the conclusion may appear expected, given the well-known vertical separation of wave forcing mechanisms: non-resolved wave forcing (NRWF) dominates in the upper stratosphere and mesosphere, while resolved wave forcing (RWF) primarily forces the lower and middle stratospheric circulation. This separation is consistent with established middle-atmosphere dynamics.

We did not explicitly mention this point in the Abstract due to the strict 150-word limit and because the Abstract focuses on stratospheric processes directly relevant to SSWs and PSCs. The distinction between RWF and NRWF is therefore developed in the Results and Discussion sections, where the vertical structure of the response can be shown explicitly. We now added the role of NRWF in the abstract.

While RWF plays the primary role in weakening the stratospheric westerlies and driving adiabatic warming in the polar vortex, NRWF also contributes significantly to the final warming response. In particular, the cooling induced by NRWF in the upper stratosphere and mesosphere propagates downward through the downward control mechanism (Haynes, McIntyre et al. 1991), influencing temperatures in the lower stratosphere and partially offsetting RWF-induced warming (see Figs. 4 and 5).

This modulating role of NRWF-induced cooling on RWF-induced warming is an important aspect of the overall stratospheric response that we think we should highlight also in the abstract. Without NRWF—especially gravity wave drag—the warming and wind weakening driven by RWF would be considerably larger throughout the stratosphere, and the net cooling in the upper levels would likely turn into warming.

Although this point was not sufficiently emphasized in the original manuscript, this comment helped us recognize the need to clarify the role of NRWF not only in the upper atmosphere but also in modulating the lower-stratospheric response. We have now revised the Discussion accordingly to better reflect this aspect too.

Changes made in response to the comment:
We now added the role of NRWF in the abstract and discusses the modulating effect in more details in the discussion to extend our mechanistic discussion (Line 309-315). Regarding the mechanisms, we have also extended the wave propagation from the troposphere to the stratosphere and discussed the possible impact of the changes in the troposphere (see response to backround wind bellow and response to comment 2 of reviewer 1). We also discussed the role of mean state as suggested in the rest of the comments (see next response)

In the abstract we have added:
"Subscale non-resolved forcing cools the stratosphere and mesosphere, modulating the stratospheric RW effect"

For additional changes related to the mechanisms please see response to next major comments.

In particular, less attention is paid to the large changes in the background winds in the different configurations and how this may affect wave propagation. In principle, in the experiments, you are modifying wave generation: including or not those related to fronts in Fr/NoFr cases, and large-scale Rossby wave trains in the AP/LSCO configurations. In addition, upward wave propagation from the lower stratosphere is also modified via changes in the background flow: conditions may be very different in the Aqua Planet vs. the LSCO experiment; even we are not shown the different wind profiles, it can be inferred both Figure 1, or the already large differences in LSCO_NoFr and LSCO_Fr in Figures 3c and 3d. It would be helpful to quantify the impacts of LSCO and SST fronts on the background conditions to interpret the role that the increased front-related wave forcing has on SSWs. Are these waves just modifying the background conditions or are they directly responsible for triggering SSW? This is important because from Figure 1, one could interpret that they are responsible for SSW triggering – they have a major impact on the variability than on the mean wind value, but SSW occurrence is

largely associated with resolved waves, how is this reconciled?
In this sense, line 12 reads "This warming significantly enhances Arctic SSW frequency and strongly reduces PSC formation."
From this statement, one could assume that SST fronts are responsible for background wind changes that favor conditions for SSWs, but, is this shown? As previously commented, Figure 1 shows that the largest effect on the mean flow is LSCO, and the effect of fronts mainly acts to increase the variability, which is more effective in producing SSWs because winds are already weaker: closer to zero (cf. Figure 1 left and right panels). Again, more details are needed in the specific role of fronts on SSW occurrence.

Response (on the role of the background wind):

We thank the reviewer for highlighting the importance of background wind conditions in shaping wave propagation and stratospheric variability. We fully agree that the background state plays a key role in modulating both the generation and the vertical propagation of planetary waves.

The importance of the background wind is well supported by the Charney–Drazin criterion (Charney and Drazin, 1961). According to this criterion, planetary waves can propagate into the stratosphere only when the zonal-mean stratospheric wind is westerly and sufficiently weak; easterlies and very strong westerlies inhibit upward wave propagation. This establishes positive feedback (e.g., Limpasuvan and Hartmann, 2000) between planetary-wave propagation and the background wind. In this feedback, when planetary waves propagate and converge in the stratosphere under weaker background winds, they decelerate the westerlies further (via the momentum budget), which in turn further enhances vertical wave propagation and convergence (consistent with the Charney–Drazin criterion), leading to an additional weakening of the stratospheric westerlies.

Since this mechanism is well established (Charney and Drazin, 1961; Limpasuvan and Hartmann, 2000), it provides a physical framework to interpret the impact of background wind conditions on wave propagation and associated SSWs, particularly in configurations where the background westerlies are already weakened, as is the case in the presence of land–sea contrast and orography (LSCO). In this preconditioned state, adding SST fronts enhances the efficiency of the Charney–Drazin mechanism and the associated positive wave–mean-flow feedback, facilitating a stronger stratospheric response. The generation of planetary waves and the weakening of the stratospheric westerlies induced by LSCO alone are consistent with the classical impact of LSCO on Northern Hemisphere stationary waves and the stratosphere (Held, 1983; Andrews et al., 1986).

Conversely, in the aquaplanet configurations designed to mimic Southern Hemisphere conditions, the classical boundary conditions for planetary-wave generation and stratospheric vortex weakening, such as LSCO, are absent, and the stratospheric westerlies remain very strong. Under these conditions, the Charney–Drazin criterion is not satisfied, and planetary waves are largely prevented from propagating vertically into the stratosphere, even in the presence of SST fronts. As a result, the feedback between planetary-wave convergence and wind deceleration is strongly reduced, maintaining a cold, undisturbed polar vortex, suppressing SSW occurrence, and favoring conditions conducive to PSC formation, even when SST fronts are present. These characteristics

highlight the critical role of background wind conditions in controlling the stratospheric response to lower boundary forcing.

In addition to the stratospheric background state, the tropospheric background circulation is also important. Features such as a deepening of the North Pacific low geopotential height or enhanced Euro-Atlantic and Scandinavian blocking have been identified as typical precursors for SSWs, as they enhance planetary-wave activity and vertical wave propagation from the troposphere into the stratosphere. In response to this comment and major comment 2 from Reviewer 1, we have expanded our analysis to track upward wave propagation from lower levels and show that SST fronts—particularly in the Pacific—strengthen the North Pacific low geopotential height (R2 Fig. 1 below and SFig. 8–9). This circulation anomaly is a well-known precursor of weak vortex states and SSWs and promotes enhanced upward planetary-wave propagation from the troposphere (Garfinkel and Hartmann, 2008; Ineson and Scaife, 2009; Nishii et al., 2009; Garfinkel et al., 2012).

In contrast, the tropospheric response to Atlantic SST fronts projects primarily onto a positive NAO-like pattern (SFig. 9), which lacks key precursory features for weak vortex events—such as high-latitude blocking over the North Euro-Atlantic and Scandinavia—and therefore produces no significant enhancement of upward planetary-wave propagation (R2Fig. 1 see also SFig. 8 in Supplements).

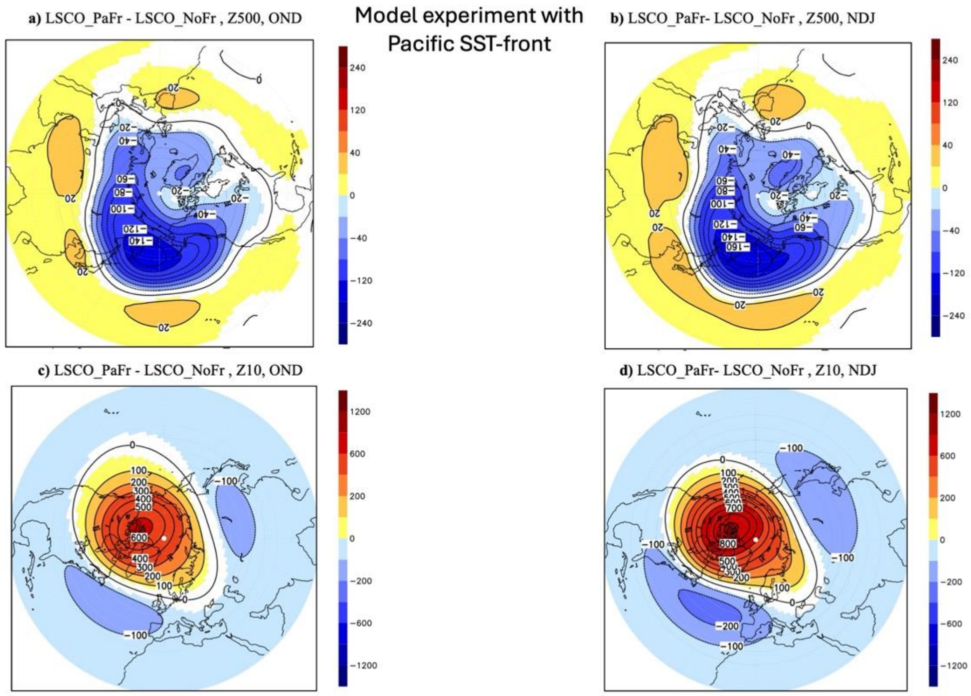

F2Fig. 1: **Model experiment with Pacific SST-front: tropospheric and stratospheric response:** (a–b) 500 hPa geopotential-height anomalies (Z500, m) and (c–d) 10 hPa geopotential-height anomalies (Z10, m) for OND (a, c) and NDJ (b, d), showing the difference between the experiments LSCO_PaFr and LSCO_NoFr. Blue (red/yellow) shading indicates negative (positive) height anomalies. The Pacific SST-front induces pronounced low geopotential height anomalies over the North Pacific, consistent with enhanced North Pacific low geopotential height anomaly and subsequence impact on the increased

upward planetary-wave propagation. This tropospheric response leads to weakened polar-vortex conditions aloft, in agreement with North pacific precursor for stratospheric change. Only anomalies significant at the 95 % confidence level (two-sided *t*-test) are shaded.

Changes made in response to the comments

We added a new paragraph clarifying the role of the stratospheric background wind in controlling planetary-wave propagation via the Charney–Drazin criterion. We explain that land–sea thermal contrast and orography (LSCO) precondition the stratosphere by weakening the mean westerlies, but that this alone is insufficient to reproduce the observed SSW frequency. We further show that the addition of SST fronts enhances stratospheric variability and tropospheric precursor conditions, allowing more efficient upward wave propagation in an already weakened background flow. This sequential weakening of the background wind provides a physical explanation for why SST fronts have a much stronger dynamical impact in the LSCO configuration than in isolation, reinforcing the wave–mean flow feedback leading to increased SSW frequency (Lines 316–329).

In response to Reviewer 1 and this comment, we expanded the analysis to explicitly address the role of tropospheric precursor circulation in mediating the stratospheric response to SST fronts. We extended the EP-flux diagnostics downward into the troposphere and added new figures (SFig. 8–9) showing that only Pacific SST fronts produce enhanced upward planetary-wave propagation from the troposphere into the stratosphere. We further demonstrate that this response is associated with strengthened low geopotential-height anomalies over the North Pacific, a well-established precursor to weak polar vortex states and SSWs. In contrast, Atlantic SST fronts project onto a positive NAO-like pattern that does not favor upward wave propagation, explaining their weaker stratospheric impact. These additions clarify the basin-specific tropospheric mechanisms linking SST fronts to SSW occurrence (Lines 269–289).

Minor comments

Another, although minor concern because should not change the conclusions of the analysis is that possible biases in the location of the stratospheric polar vortex are not taken into account in Figure 1. Note that Figure 3 shows an equatorward bias of the maximum strength of the winds, so it would be misleading to use this same unique latitude (60N) to evaluate the stratospheric polar vortex in all the experiments, and another approach as a latitudinal average would be more precise.

We repeated our SSWS detection using the averaged latitudes 55:65. This does not change the number of SSW events, but in few cases the central day of the SSW events shif by 1 day later. Meaning that the consideration of larger latitude band may need 1 day more to get latitudinaly averaged reversal of the westerlies.

It should be specified in the abstract that the experiments are semi-idealized because not including sea ice is an important fact considering the number of studies that relate stratospheric polar vortex interactions to Arctic sea-ice (e.g. recently, Liang et al 2024 or Xu et al. 2024).

We have added **“semi-idealized experiments”** explicitly to the Abstract. In addition, we have analyzed the effect of adding **Arctic sea ice**, in new experiment (SFig. 10; see also response to Comment 5 of Reviewer 1). We find that including the Arctic sea-ice does **not significantly change** the impact of the SST fronts on the stratospheric circulation. The stratospheric response to SST fronts remains **quasi-similar** in the presence and absence of Arctic sea ice.

Line 141: Have you considered using NRWF or non-RWF? Using RWF vs NRF is confusing to me.

We used NRF because the residual is not always sub-scale wave forcing; near the surface, friction dominates the residual. However, since our paper focuses on the stratosphere and mesosphere, where gravity waves dominate the residual, we believe it is appropriate to use NRWF. We now indicate explicitly that the residual in the stratosphere and mesosphere is dominated by gravity wave drag, and we therefore adopt NRWF instead of NRF.

Lines 163-165: Is it balanced by anomalous diabatic cooling? This is confusing, Are you just referring to a particular equation?

Yes Equation (2) the $\bar{Q}^{dia}$ -term. This term is just the residuum in the equation (2).
We have made that clearer by writing:
The response of the adiabatic warming $= -\bar{w}^{*}\bar{\theta}_{z}$ is balanced mainly by anomalous diabatic cooling $\bar{Q}^{dia}$ (not shown, Equ. 2 Methods).

Lines 200-201: “However, its influence is weaker compared to the LSCO effects.” Aren’t the LSCO effects translated into RWF? Or you mean the total, TWF?

Here we mean RWF. This is valid for both.
We changed it to:
However, the influence of RWF on BDC strengthening—and therefore also of TWF—in the SST-front experiment is weaker compared to their effect in the LSCO experiment

Lines 207-210: I very much agree with this statement but it’s quite repetitive as in lines 179-181
Line 645: “Negative dynamical forcing drives positive Coriolis forcing”. This does not seem accurate since that momentum does not drive Coriolis forcing, more accurate is that the Coriolis forcing term (the term) increases.

We have used “increases “ or “associated” instead of “lead” or “dirve” and have referred to the momentum budget, that explain how the balancing relation between RWF and Coriolis forcing will change the Coriolis forcing and the meridional residual circulation.

Line 191,192 and other parts of the text: LSCO-effects, SSW-frequency and PPSC-conditions, I don’t think you need the hyphen in these cases because they’re not a compound adjective followed by a name.

We also agree on that.

Discussion:
Interesting to see how oceanic fronts become so important when LSCO is present. In this sense, I am missing some discussion on the role of orography that has been explored before (e.g. White et al. 2018).

We thank the reviewer for highlighting the importance of orography and for referencing White et al. (2018). Such reference was missing in our paper.
We fully agree, the orography plays a central role in shaping the background conditions that govern upward planetary wave propagation into the stratosphere. As discussed in response to the comments on role of background wind, the weakening of the westerlies in the presence of LSCO plays crucial role in making the conditions favorable for Charney-Drazin chritereon, which strengthens the impact of SST-fronts on the stratosphere when the LSCO are present.

Compared to White et al. 2018, in our LSCO configuration, both land–sea thermal contrast and realistic orography are included and not only the Orgraphic impact, and together they enhance the generation of planetary wave activity. We now cite White et al. (2018) in the Introduction to support the well-established impact of LSCO on stratospheric dynamics and have added a more explicit discussion of LSCO effects in response to the major comments.

Our results show that orography, in combination with land–sea contrast, weakens the stratospheric polar vortex and creates conditions favorable for further disruption by SST fronts. Although we do not isolate the impact of orography alone, our findings remain consistent with those of White et al. (2018), who demonstrated that orographic wave forcing leads to EP flux convergence and stratospheric wind deceleration.

Changes made:
We have discussed white et al. 2018 in differen text passages. In the introduction as part of the theoretical motivation and before that (L74 and L120-122). We also have added a more explicit discussion of LSCO effects in response to the other major comments.

Detected typos:

Line 59: Do you mean wave-mean flow interactions? Please, revise
Yes. Changed

Line 144: details -> detailed
Changed

Line 151: please revise “componeaffents accelerate”
Changed to : components RWF and NRF

Line 259: Ozon -> Ozone

done

Line 216,308: “conductive” conducive, as you used in other parts of the text, is better.
Done

Line 285: SSW -> SSWs
We have rewritten this section in response to reviewer 1

Line 296: “which as we will show” change for “which as shown in Results” or similar since Methods appear at the end of the manuscript.

We have rewritten this section in response to reviewer 1

Line 316: AP_NoFr-configuration -> AP_NoFr configuration
done

Line 352: Eulerian Eulerean
Done

Line 368: temperature
Done

Line 380: “residuum”
Done
Line 397: “term..”
Done

Line 401: origin
Done

Figures 3-5: y-axis label reads “Air_pressur”, revise.
Done

Figure 5a: there’s a white arrow in the bottom right, does this mean something?
No thanks, removed

Figure 5d is missing the Height values.
Done

Figure 6: The vertical axis reads Height, but it should be Pressure level.
Done

Supplementary Material: Please, revise the formatting of figure captions.
Done

Reference:

- Andrews, D. G., J. R. Holton and C. B. Leovy (1987). Middle Atmosphere Dynamics, Academic Press.
- Cagnazzo, C. and E. Manzini (2009). "Impact of the Stratosphere on the Winter Tropospheric Teleconnections between ENSO and the North Atlantic and European Region." Journal of Climate 22(5): 1223-1238.
- Charney, J. G. and P. G. Drazin (1961). "Propagation of Planetary-Scale Disturbances from the Lower into the Upper Atmosphere." Journal of Geophysical Research **66**(1): 83-109.
- Haynes, P. H., M. E. McIntyre, Shepherd, T. G., C. J. Marks and S. K. P. (1991). "On the ‘‘downward’’ control of extratropical diabatic circulations by eddy induced mean zonal forces,." Journal of the Atmospheric Sciences 48(651– 680).
- Held, I. M. (1983). Stationary and quasi-stationary eddies in the extratropical troposphere: Theory. Large-Scale Dynamic Processes in the Atmosphere. B. J. Hoskins, Pearce, R. San Diego, CA, Academic Press: 127-168.
- Hines, C. O. (1997). "Doppler-spread parameterization of gravity-wave momentum deposition in the middle atmosphere .2. Broad and quasi monochromatic spectra, and implementation." Journal of Atmospheric and Solar-Terrestrial Physics **59**(4): 387-400.
- N. A. Mcfarlane, The Effect of Orographically Excited Gravity-Wave Drag on the
- Genera Circulation of the Lower Stratosphere and Troposphere. *J Atmos Sci* **44**, 1775-1800 (1987).
- C. O. Hines, Doppler-spread parameterization of gravity-wave momentum deposition in the
- middle atmosphere .2. Broad and quasi monochromatic spectra, and implementation. *J Atmos Sol-Terr Phy* **59**, 387-400 (1997).
- C. O. Hines, Doppler-spread parameterization of gravity-wave momentum deposition in the middle atmosphere .1. Basic formulation. *J Atmos Sol-Terr Phy* **59**, 371-386 (1997).
- Karpechko, A. Y. and E. Manzini (2012). "Stratospheric influence on tropospheric climate change in the Northern Hemisphere." Journal of Geophysical Research-Atmospheres 117.
- Omrani, N. E., N. S. Keenlyside, J. Bader and E. Manzini (2014). "Stratosphere key for wintertime atmospheric response to warm Atlantic decadal conditions." Climate Dynamics 42(3-4): 649-663.
- Limpasuvan, V. and D. Hartmann (2000). "Wave-Maintained Annulare Modes of climate Variability." Journal of Climate: 4414-4429.
- Mcfarlane, N. A. (1987). "The Effect of Orographically Excited Gravity-Wave Drag on the General-Circulation of the Lower Stratosphere and Troposphere." Journal of the Atmospheric Sciences **44**(14): 1775-1800.

Reviewer #3 (Remarks to the Author):

We thank Reviewer 3 for the thorough reading of the manuscript and for the detailed and critical comments. The reviewer's remarks prompted us to substantially improve the clarity, structure, and theoretical grounding of the paper. In particular, the comments motivated us to clarify the physical and mathematical rationale of the experimental design, explicitly address the role of linear additivity and background-state dependence in isolating SST-front effects, and strengthen the theoretical framework linking SST fronts, baroclinic wave generation, background wind conditions, and stratospheric wave propagation. The reviewer's feedback also helped improve the presentation of diagnostics, figure interpretation, and the broader relevance of the study, thereby significantly strengthening the manuscript.

This work aims to isolate and understand the impact of midlatitude SST-fronts on the frequency of SSWs and climatic conditions conducive to the PSCs. However, after reading the entire manuscript, I find that neither of the two stated goals is clearly addressed.

(A) The impact of SST-fronts cannot be considered in isolation, as it is inherently influenced by land-sea thermal contrast and orography.

In this work, the authors 'isolate' the impact of SST-fronts by calculating the differences between LSCO_Fr and LSCO_NoFr (Fig. 3 b&d and Fig. 5). This difference shows the impact of midlatitude SST-fronts with interference from land-sea thermal contrast and orography (LSCO). First of all, what the authors should do is to calculate the difference between AP_Fr and c, which represent the impacts of SST fronts in the absence of LSCO. Beyond that, I don't think the impact of SST-fronts can be isolated, given the non-linear interaction between SST-fronts and LSCO. The non-linear interaction between SST-fronts and LSCO can be inferred from Supplementary Table 1. In particular, AP_NoFr vs. AP_Fr suggests that adding SST-fronts alone does not substantially increase SSW-frequency; AP_NoFr vs. LSCO_NoFr suggests that adding LSCO alone also does not substantially increase SSW-frequency. It is only when both SST-front and LSCO (LSCO_F) are included that one can see an SSW frequency comparable to the observations.

We appreciate the reviewer's concern regarding the interaction between SST fronts and LSCO. This comment highlights the need to better explain the physical rationale and structure of our experimental design.

In the following, we will argue both physically and mathematically that, for understanding SST-front effects in the **Northern Hemisphere**, only the difference **LSCO_Fr – LSCO_NoFr** is appropriate. This is because SST fronts in the NH naturally coexist with land–sea thermal contrast and orography (LSCO), which fundamentally shape the NH atmospheric circulation. In

contrast, the difference **AP_Fr – AP_NoFr** is designed to mimic the **Southern Hemisphere** background — a more zonally symmetric SST-configuration without LSCO — and is therefore not suitable for assessing NH dynamics. We didn't considere this difference in details because the impact of the SST-front on the stratospheric SSWs and PPSC in the aquaplanet (mimicking the SH) is minimal, which we will see is consistent with theory from the perscpective of Charney-Drazin criterion.

On the Linearity and Physical Interpretability

We agree that nonlinear interactions may exist between SST fronts and LSCO. But this occurs only if one adds the effect of SST fronts in the aquaplanet with the LSCO effect together — which physically doesn't make sense, since SST fronts in the Northern Hemisphere act in the presence of LSCO. However, if we considere our LSCO_Fr as front experiment we can show both mathematically and physically that the linearity is natural consequence. To clarify this, we provide a mathematical demonstration of additivity that supports our experimental framework, demonstrate this linearity in our model experiment and shows that the linearity in the NH is accomplished only in the framwork of our experiment setup and not whene considering AP_NoFr as non-front experiment.

Let us define the configurations:

- **AP_NoFr**: Aqua-planet without SST fronts
- **AP_Fr**: Aqua-planet with SST fronts
- **LSCO_NoFr**: LSCO-comfiguration without SST fronts
- **LSCO_Fr**: LSCO-configuration with SST fronts

From these considerations, we should have:

a) **LSCO effect alone**: LSCO_NoFr − AP_NoFr (since LSCO_NoFr and AP_NoFr differ only thorugh **LSCO**)
b) **SST-front effect in SH-like background**: AP_Fr − AP_NoFr (since AP_Fr and AP_NoFr differ only through SST-fronts in SH-like conditions i.e Aqua-planet)
c) **SST-front effect in NH-like background**: LSCO_Fr − LSCO_NoFr (since differ only through SST-front in NH-like condition, i.e. in the presense of LSCO)
d) **Combined LSCO and SST-front effect for NH-like conditions**: LSCO_Fr and AP_NoFr (since LSCO_Fr and AP_NoFr differ through both LSCO and SST-fronts)

Now consider the implications of linear decomposition:

1. **Incorrect/physically invalid assumption:**

   If we add (b), the SST-front effect in aquaplanet condition (SH-like background) (i.e. AP_Fr − AP_NoFr) and (a), the LSCO effect alone (i.e. LSCO_NoFr − AP_NoFr) we don't

get (d), the combined LSCO and SST-front effect for NH-like conditions (LSCO_Fr − AP_NoFr):

(AP_Fr − AP_NoFr) + (LSCO_NoFr − AP_NoFr) = AP_Fr + LSCO_NoFr − 2 × AP_NoFr

→ This does not correspond to any realizable model experiment and is not physically meaningful. So, the proposed AP_Fr − AP_NoFr as effect of the SST-fronts in linearity doesn't work.

2. If we add (c), the SST-front effect in NH-like background (i.e. LSCO_Fr − LSCO_NoFr) and (a), the LSCO effect alone (i.e. LSCO_NoFr − AP_NoFr) we exactely get (d), the combined LSCO and SST-front effect for NH-like conditions (LSCO_Fr − AP_NoFr):

   (LSCO_NoFr − AP_NoFr) + (LSCO_Fr − LSCO_NoFr) = LSCO_Fr − AP_NoFr

   → Therfore sum of (a) and (c) exactly recovers the combined LSCO + SST-front effect and corresponds exactly to our actual model experiments. This can be shown not only theoretically but also from our experiment setup (see figure bellow R3Fid. 1 and R3Fig. 2). So, the effect of (a) and (c) add linearly and therefore our experiment setup is valid for our purpose.

Thus, our formulation using **LSCO_Fr – LSCO_NoFr** to isolate the SST-front effect under NH conditions is not only mathematically well-posed but it can also be proved from the model output.

Physical Justification

In real-world NH dynamics, SST fronts are embedded in a zonally asymmetric background shaped by continents and orography (LSCO). These features (LSCO) are essential sources of planetary wave activity and strongly modulate the background wind. Specifically:

- LSCO weakens the zonal-mean westerlies and accelerate upward wave propagation through the Charney–Drazin criterion [Charney & Drazin, 1961]. In this criterion the upward planetary wave propagation requires westerly but not too strong winds. Strong westerlies suppress vertical propagation; thus, LSCO preconditions the stratosphere for upward propagation by weakening the vortex, which can partially explain the enhanced impact of the SST-front on the stratosphere in the presense of LSCO.

- In addition, the SST fronts further enhance tropospheric baroclinicity and eddy driven jet (Omrani et al. 2019), particularly over the Pacific, which by geostrophic balance promots precursors such as a deepened high latitude geopotential height (Sfig. 9, see Garfinkel et al., 2010; Nishii & Nakamura, 2010), which are well known to trigger upward planetary wave propagation into the stratosphere.

- This two-step interaction — LSCO weakening background wind and SST fronts enhancing wave forcing — supports the feedback loop where planetary waves weaken the vortex, enabling further wave propagation and eventual SSWs.

By contrast, in Aqua-Planet (AP) configurations, the strong stratospheric westerlies and lack of planetary wave sources prevent upward wave propagation, even in the presence of SST fronts. This explains why AP_Fr – AP_NoFr produces weak stratospheric response in SSWs and strong PPSCs — consistent with Southern Hemisphere dynamics.

Conclusion

In summary, our choice to isolate the SST-front effect as **LSCO_Fr – LSCO_NoFr** reflects the only physically meaningful and experimentally realizable approach to study SST fronts under Northern Hemisphere–like conditions. It also allows for clean decomposition of effects that is consistent with both mathematical linearity and atmospheric theory. We have clarified this rationale in the revised manuscript and now include both the theoretical and mathematical justification in the Supplementary Information.

Changes in the paper:
We clarified the experimental design logic by explaining why SST-front effects in the Northern Hemisphere must be evaluated relative to a background that includes land–sea thermal contrast and orography (LSCO), which are inseparable components of the real NH system. We further explained that the aqua-planet configuration provides a Southern Hemisphere–like baseline, allowing SST-front effects to be isolated consistently in both hemispheric contexts. We also clarified that the combined LSCO and SST-front response satisfies mathematical additivity and can be decomposed into individual LSCO and SST-front contributions, a property confirmed by the model output (Lines 414–426).

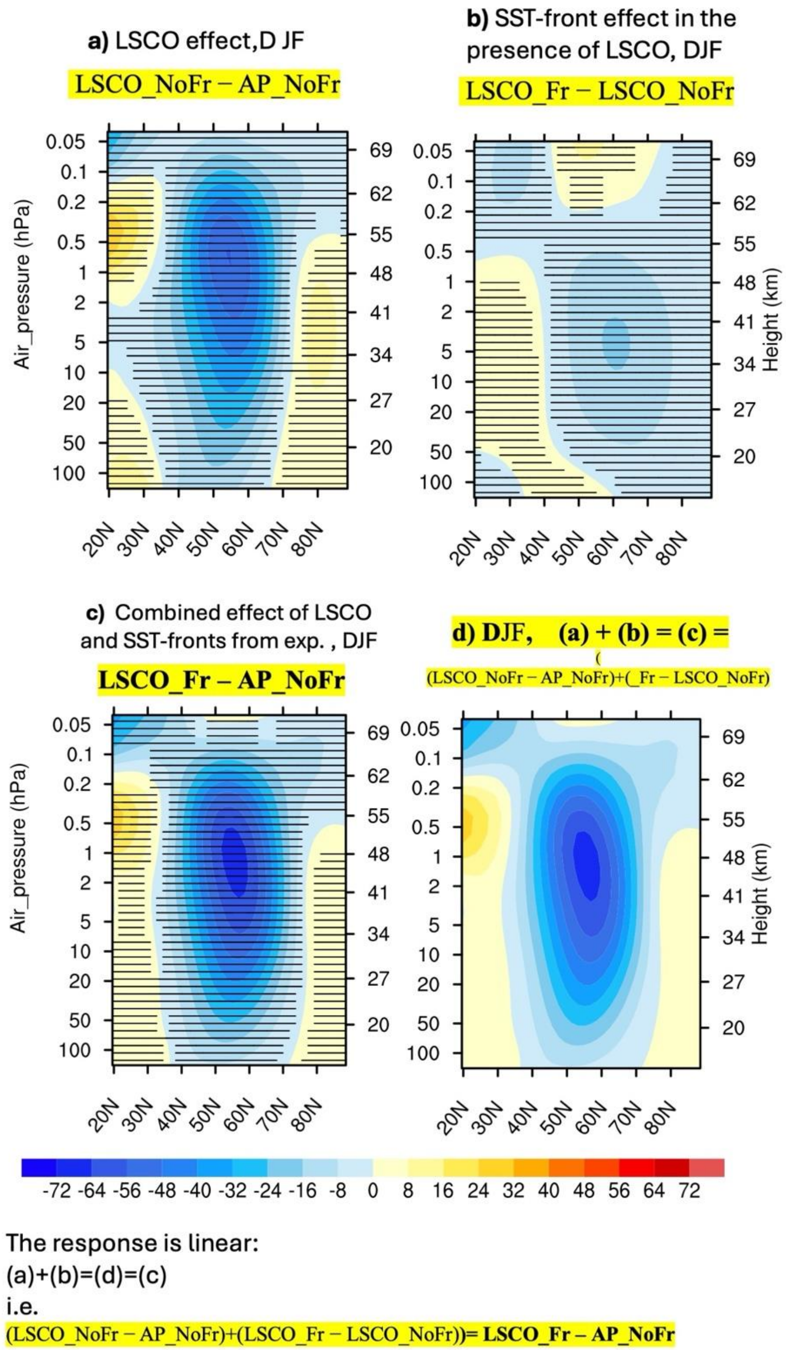

Figure R3.1: Additivity of LSCO and SST-front effects on [e.g., zonal wind anomaly for DJF]. Panels (a) and (b) show the individual LSCO and SST-front effects in our experiment; panel (c) shows the combined response from our experiment; panel (d) shows the linear sum of (a) and (b). The close agreement between (c) and (d) confirms the linearity of the system under this decomposition.

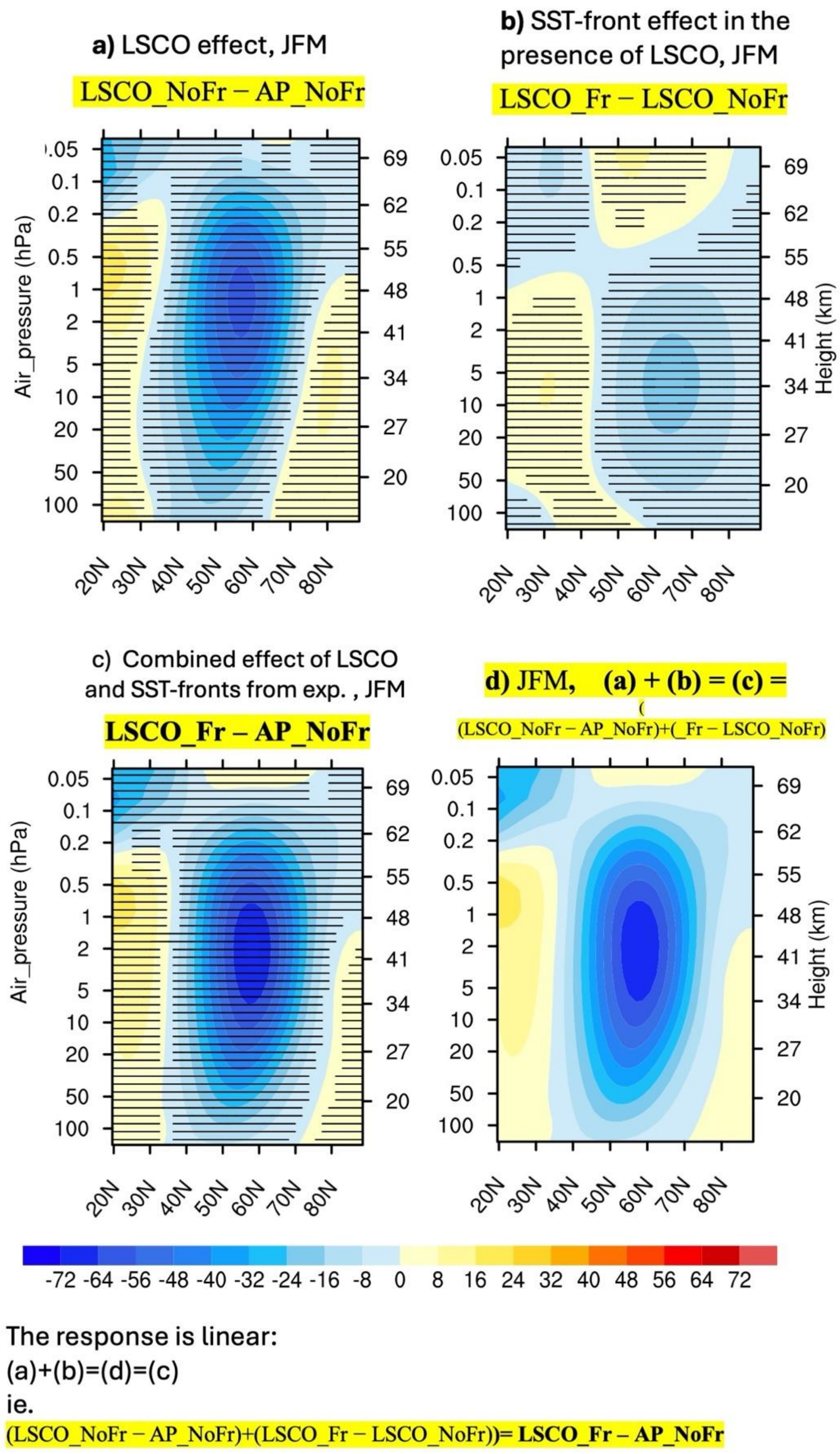

Figure R3.1: like **R2.1** but for JFM

(B) The study relies heavily on model simulations but lacks robust theoretical justification.

The findings of this study are largely derived from a single model's experiments. Although the model results show some agreement with reanalysis (Figs. 1-2), such agreement can arise by coincidence or may stem from compensating errors, rather than an accurate representation of the underlying physical processes. Therefore, a comprehensive theoretical framework is required to support the robustness of the results. However, this study lacks a thorough examination of the underlying mechanisms behind the results. In the absence of observational and theoretical support, modeling results alone cannot provide conclusive scientific insight. In fact, the manuscript lacks a clear logical structure, well-articulated scientific ideas, and a rationale analytical foundation. For example, regarding "What drives the North-South asymmetry of SSWs and PSCs?", after briefly introducing the role of orography and land-sea thermal contrast first, the authors jumped to say "Here, we propose a crucial missing link: Sea Surface-Temperature (SST) fronts" (see lines 61-67). What is your scientific thinking behind this proposal? How can you expect it to be 'crucial'? Without a clear explanation of the scientific rationale behind this proposal, it risks appearing as an arbitrary choice, lacking a meaningful physical connection to the core topic of the study.

While we did not explicitly include a full theoretical overview in the original manuscript—primarily to keep it as simple and as short as possible—we thank the reviewer for raising this issue. It has prompted us to clarify and expand the theoretical background underpinning our approach, and in doing so, we were able to articulate more clearly the physical rationale we were aiming to present, which we believe has increased the quality of the paper.

Our experimental design is guided by well-established dynamical theory. The key theoretical mechanisms underlying our study are grounded in linear baroclinic wave theory and the Charney–Drazin criterion (Ead, 1949, Charney, 1947, Charney and Drazin 1961). Baroclinic wave theory links SST fronts to enhanced low-level baroclinicity, which generates stronger baroclinic eddies, strengthening of the eddy-driven jet (Omrani et al. 2019, Sampe and Nakamura, 2010 and Sampe et al. 2022). In the Pacific this sets up tropospheric circulation conducive to upward wave propagation -such as strengthening of low geopotential hight in the North Pacific (Aleutian low)- (see details 1 below, Garfinkel and Hartman, 2008, Garfinkel et. al. 2012, Nishii et. al. 2009 and Inesoon ans Scaife 2009). The strengthening of the North Pacific low geopotential hight is well known as a precursor for stratospheric vortex weakening and Sudden Stratospheric Warmings (SSWs). The other motivating theoretical background is the Charney–Drazin criterion, which states that planetary waves can only propagate vertically into the stratosphere when the zonal-mean winds are westerly but not too strong. This is the basic for the well-known positive feedback between wave propagation and the mean flow (see below for details). This framework explains why introducing SST fronts under LSCO conditions—where the background stratospheric westerlies are already weakened—promotes stronger vertical wave propagation. In contrast, in the Aqua-Planet configuration, where the background westerlies are strong, the Chrney-Drazin criterion predict strong weakning of upward wave propagation in strong background westerly wind, and the stratospheric response is much weaker (see details 3 and 4 below). Our theoretical

background can also be used to predict the lack of the Atlantic SST-front in exiting the Polar vortex and SSWs (see details. 2)

Details

Details .1: Baroclinic Wave Generation from SST Fronts in the Pacific

Linear wave theory (Ead, 1949, Charney, 1947) suggests that strong meridional SST gradients, such as those associated with oceanic fronts in the Pacific and Atlantic, increase low-level baroclinicity (Omrani et al. 2019), which enhances the generation of baroclinic eddies.

- In the Pacific, this enhanced eddy activity strengthens the eddy-driven jet, and by geostrophic balance, deepens the low gropotential hight in the North Pacific (Aleutian Low) (Omrani et al. 2019, SFig. 9)
- The low geopotential hight in the North Pacific is a well-known precursor of SSWs, as it enhances upward planetary wave propagation into the stratosphere (e.g., Garfinkel et al. 2010; Nishii & Nakamura 2010, Inocent and Scaife 2009).

Details2. Why the Atlantic SST-front is less efficient in weakening the stratospheric vortex and generating more SSWs

The Strengthening of the Atlantic eddy-driven jet in response to Atlantic SST-front results by geostrophic balance in a positive NAO-like patter (SFIg. 9), which has not been shown to act as precursor for SSWs and weakening if the vortex. The well-known Atlantic percussor for SSW and weakening of the stratospheric vortex are Euro-Atlantic and Scandinavian Hight (Nishii & Nakamura 2010), which does not appear as response to SST-front. From the linear wave theory and our knowledge of the SSW-precursor, we can even predict the reduce capabilty of the Atlantic SST-front in generating SSWs.

Details 3. Stratospheric Background State and LSCO

Consistent wirth our results, Previous studies (e.g., White et al. 2018) have shown that orography significantly weaken the Northern Hemisphere stratospheric polar vortex. This sets up a more favorable background state for planetary wave propagation for SST-fronts, particularly under the Charney–Drazin criterion, which requires sufficiently weak stratospheric westerlies for vertical wave propagation to occur.

Details. 4 Positive Feedback under Charney–Drazin Conditions

When planetary waves propagate upward and converge in the stratosphere, they decelerate the westerlies. This deceleration further enhances the conditions for wave propagation, establishing a positive feedback loop that can culminate in an SSW — especially when the background westerlies are already weakened (as in our LSCO_Fr experiment).

Response to vthe comments:

We clarified that our experimental design and interpretation are grounded in established dynamical theory, specifically linear baroclinic wave theory and the Charney–Drazin criterion. We explained how SST fronts enhance low-level baroclinicity and the eddy-driven jet, particularly in the Pacific, thereby favoring tropospheric precursor patterns that promote upward planetary-wave propagation and SSWs. We further clarified that vertical wave propagation depends on a weakened background westerly flow, which is facilitated by land–sea thermal contrast and orography (LSCO), while aqua-planet configurations suppress this mechanism due to strong zonally symmetric winds. These

theoretical considerations provide the physical basis for the explaining stratospheric responses across configurations (Lines 112–127).

(C) The manuscript has a highly specialized focus and may not meet the general scope of the journal.

We thank the reviewer for raising this concern. While our study focuses on specific dynamical processes, we believe the topic is broadly relevant to the geoscience community. Sudden stratospheric warmings (SSWs) are well known to influence surface climate and extreme weather events, while polar stratospheric clouds (PSCs) are visually striking phenomena and play a central role in polar ozone depletion and radiative forcing.

Understanding the drivers of these processes is essential for advancing our knowledge of Earth system dynamics. The impact of oceanic SST fronts explored in this work is not only scientifically interesting and new but also important for improving model biases, seasonal-to-subseasonal predictability, and our understanding of climate sensitivity in both hemispheres.

We agree that the underlying mechanisms are complex; however, this complexity reflects the nature of the coupled atmosphere–ocean–stratosphere system. We have therefore tried to explain the key physical processes and dynamical links as clearly as possible while maintaining scientific rigor. We hope this balance supports the broader relevance of the work to the journal's readership.

Changes in the paper:
We clarified that understanding SSWs and PSCs requires a dynamical understanding of the Brewer–Dobson circulation and its control by planetary wave activity and wave–mean flow interactions, and we highlighted that these stratospheric processes have broader implications for surface climate, ozone chemistry, and climate-model fidelity (Lines 66–69).

The manuscript mentions PSCs and stratospheric ozone depletion but does not provide a direct or detailed analysis of these topics. The focus on temperature and wind anomalies may limit its relevance to the wider scope of the journal.

We thank the reviewer for this observation. In our study, we use a **widely accepted proxy for PSC formation**, namely the **temperature threshold of 195 K and 188K**, which corresponds to the conditions required for the formation of Type I PSCs (nitric acid trihydrate clouds) and type II (water). This approach is commonly used since decades in stratospheric studies to diagnose the potential for PSC formation for both observation and models and has strong support in the literature (Poole & McCormick (1988), Peter (1997), Solomon (1999), Drdla & Schoeberl (2003), Rex et al., (2004; 2006), Manney et al. (2011)).

Regarding stratospheric ozone depletion, we agree that our manuscript does not simulate chemical ozone loss directly. However, the formation of PSCs is a key precursor and therefore also predictor

of polar ozone depletion (Solomon et al. (1986), Hanson & Ravishankara (1994), Tabazadeh et al. (2000), Tabazadeh et al. (2000)), as PSCs provide the surfaces needed for heterogeneous chemical reactions that convert reservoir species (e.g., HCl and $ClONO_2$) into active chlorine (e.g., ClO), which then destroy ozone in the presence of sunlight. Thus, the dynamical modulation of PSC conditions—via temperature changes induced by SST fronts and LSCO—has direct implications for ozone loss, even if ozone chemistry is not explicitly modeled here.

Therefore, while our focus is on dynamical drivers, the temperature-based PSC proxy links directly to the physical preconditions for ozone depletion, underscoring the relevance of our results to stratospheric composition and broader Earth system processes.

Other comments:

Line 3: "such as Sudden Stratospheric Warmings (SSWs)" ->

such as major Sudden Stratospheric Warmings (SSWs)

done

Lines 3-4: "Extreme stratospheric events such as ... Polar Stratospheric Clouds (PSCs)"

The term "Polar Stratospheric Clouds (PSCs)" generally refers to a phenomenon rather than an event. This also applies to line 272.

Done, we have removed extrem events in the abstract (the abstract should be kept short) and written "extreme cooling events conductive to PSCs" instead of just PSCs in line 272

Lines 49-51: I'm not sure about the relevance of mentioning SSW in this sentence. Is the first part of this sentence necessary?

We have reformulated the sentence to put the focus on Antarctic-like ozone hole in the Arctic

Line 55: "The BDC is typically stronger in the NH than in the SH winter."

Please provide some references for this statement.
Done

Lines 55-56: "A strong BDC drives polar adiabatic warming and weaker polar vortex, ..."

maybe say "In the NH, a strong BDC drives polar adiabatic warming and weaker polar vortex, ...

"
Done

Lines 66-67: "These sharp oceanic temperature gradients, associated with western boundary currents, play pivotal roles in atmospheric dynamics (33-38)."

Please describe how the SST fronts impact atmospheric dynamics.

We have added a detailed section explaining how SST fronts influence atmospheric dynamics by Extending the EP-flux into troposphere, analyzing the tropospheric precursors and discussing the Charney-Drazin creterions (Lines 269-289 and Lines 316-329). We refered to our previous work (Omrani et al. 2019) and linear wave theory to show how the SST-fronts lead to precursory tropospheric response (required for stratospheric response) through their role in enhancing low-level baroclinicity and modulating planetary wave activity.

Lines 69-70: "While previous studies have explored their role in shaping the tropospheric eddy-driven jets, storm-tracks, blocking events and stratospheric polar vortex"

Please provide some references for these statements, as well as describe how the SST fronts shape these systems.
Done

Lines 129-130: "anomalous negative windshear (increasing easterly anomalies with height), whereas anomalous negative thermal gradient in the uppermost stratosphere and mesosphere corresponds to anomalous positive windshear (decreasing easterly anomalies with height)."

The correspondence is not that clear here. It is not easy to imagine the picture of a windshear based on the figure shown.

Above ~1 hPa, the absolute magnitude of the zonal-mean wind anomalies (which are easterly) **decreases** with height — that is, the easterlies become weaker with height. Since the zonal-mean wind anomalies are negative (i.e., easterly), this vertical structure implies a **positive** vertical gradient in the zonal wind ($\partial\bar{u}/\partial z > 0$), corresponding to a **westerly wind shear**.

At the same levels, the meridional temperature gradient becomes negative — that is, polar cooling and lower-latitude warming lead to $\partial\bar{T}/\partial y < 0$. According to thermal wind balance, $\partial\bar{u}/\partial z$ is proportional to $-\partial\bar{T}/\partial y$. Therefore, the positive vertical wind shear is consistent with the negative meridional temperature gradient observed.

The inverse relationship is seen below 1 hPa, where $\partial\bar{u}/\partial z$ becomes negative (easterly shear) and the meridional temperature gradient becomes positive (i.e., polar warming relative to midlatitudes), again consistent with thermal wind balance

Changes made:

We have added more details explaination of the thermal wind balance in lines L174-175 describing the mathematical relation between the vertical wind shear and meridional T-gradiet. This will make it easier to understand this relation.

Lines 131-132: "Adding SST-fronts overall amplifies these patterns,"

The impact of adding SST-fronts can also be shown by calculating the difference between AP_Fr and AP_NoFr. Do you get the same result?

We thank the reviewer for this observation. It should be **“Adding SST fronts to the LSCO_NoFr configuration amplifies.** Here, we refer to adding SST fronts to the **LSCO_NoFr** configuration, not to **AP_NoFr**. Only this makes sense for the nordern hemisphere to satisfy the lineatrity (see response to previous comments on linearity). This is represented by the difference **LSCO_Fr – LSCO_NoFr**, shown in **Fig. 3b & 3d**. The effect of LSCO alone, without SST fronts, is shown in **Fig. 3a & 3c** as **LSCO_NoFr – AP_NoFr**.

In contrast, **Fig. 3b & 3d** isolate the effect of adding SST fronts to the existing LSCO_NoFr background — that is, the SST-front effect on top of LSCO. These SST fronts are not zonally symmetric but specifically represent the **Atlantic and Pacific frontal zones**.

To clarify this in the text, we have revised the phrase “Adding SST fronts amplifies…” to: **“Adding SST fronts to the LSCO_NoFr configuration amplifies…”**

Lines 133-134: "While these responses are weaker compared to those to LSCO"

How do you reach this conclusion? Did you calculate the difference between a&b, as well as between c&d?

We thank the reviewer for this observation.

Our conclusion that the responses to SST fronts (Figs. 3b & 3d) are weaker than those to LSCO (Figs. 3a & 3c) is based on the visual comparison of the color bar values. The color bar in Fig. 3a & 3c (LSCO effect) has noticeably higher magnitude ranges than that in Fig. 3b & 3d (SST-front effect). So, we do not need the difference if we compare the scale of the color bars. The differences are very clear in this case. Thanks for making this point point clear. We must make it clearer for the reader in the figure captions.

To improve readability within each subplot, we chose slightly different color bar ranges — since using a single, uniform scale for all panels would make the relatively weaker signals in Fig. 3b & 3d visually indistinct, given the limited number of discrete colors available.

We now clarify the differences in scales of the color bars in the caption of Fig. 3 and other figures, where the values of the colorbar differes, to make this distinction transparent to the reader.

Lines 157-158: "across the extratropical stratosphere, resulting in notable weakening of the westerlies"

Do you show the changes of zonal winds here? If not, please plot a box in Fig. 4 to indicate the domain of change in dynamical drag, as well as a box in Fig. 3 to indicate the domain of change in zonal wind. This will allow the readers to see if there is a correspondence between the two.

The zonally averaged zonal wind is shown in Fig. 3c, where we observe a significant (hatched blue area) weakening of the westerlies throughout the extratropical stratosphere (approximately 100 hPa to 0.5 hPa) between 40°N and 70°N. The negative total wave forcing (TWF) in Fig. 4a corresponds spatially with this weakening, supporting a consistent dynamical interpretation based on the momentum budget in Equation 1.

This diagnostic approach, relying on the momentum budget, is well-established in atmospheric dynamics. Importantly, changes in zonal wind are not solely driven by local wave forcing; they also reflect **non-local effects** transmitted through the residual mean circulation—an effect widely recognized as **downward control** (Haynes et al. 1991).

We have attempted to clarify these mechanisms in detail, particularly in the schematic in Fig. 6. When downward control and thermal wind balance are considered, the spatial structure of wave forcing, temperature, and wind changes aligns consistently across our diagnostics.

Text clarification: We now specified the region of interest in the main text as region between 40°N and 70°N and under 0,5hPa (lines 206-207)

We chose not to highlight this region with a box in the figures, as such annotations are often interpreted as defining a region used for regional averaging or index calculation. To avoid potential confusion, we instead clearly define the relevant area directly in the text, where its physical significance is explained in context.

.
Line 159: "which corresponds to the strengthening of the BDC, particularly its downward branch"

You don't expect all readers to understand BDC and its downward branch. To make it easier for your readers to follow, please add a box to show where you refer to (e.g., where the downward branch is). This will allow the readers to know where they should focus on your figures, especially when most of the figures are busy and noisy.

The downward branch of the Brewer–Dobson circulation (BDC) in Fig. 4 can be read directly from the residual mean stream function, provided its definition is understood. This therefore requires explanation rather than additional marking.

A positive residual stream function corresponds to clockwise circulation, implying poleward motion in the lower to mid-latitudes followed by downward motion at high latitudes and over the pole, associated with adiabatic warming. Conversely, a negative residual stream function corresponds to anticlockwise circulation, implying equatorward motion in the mid-to-high latitudes and upward motion at high latitudes and over the pole, associated with adiabatic cooling.

Accordingly, the downward branch of the BDC corresponds exactly to the red hatched regions (adiabatic warming) in Fig. 4d. It coincides with the northern branch of the positive residual stream-function contours, which rotate clockwise—indicating poleward flow in mid-to-high latitudes and downward motion between approximately 50°N and 90°N. This region therefore corresponds precisely to where adiabatic warming occurs due to subsidence, since downward motion leads to compressional warming.

The downward branch does not occupy a simple rectangular area and is therefore difficult to delineate with boxes or straight lines. Instead, it is unambiguously identified through the combination of positive residual circulation contours and the associated red (warming) shading.

Changes made in the paper:

We explained the dynamics and structure of the BDC including its downward branch in L61-66. We also explicitly explained how the residual mean stream function and adiabatic temperature tendencies should be read from the plots. This clarification has been added in the figure captions, to guide readers unfamiliar with residual circulation diagnostics.

Line 160: "drives mid-to-high latitude adiabatic warming (Fig. 4d),"

Please indicate the altitude of this warming. Again, add a box to indicate where you refer to.

Please see previous response. The altitude of the significant warming are exactly the regions dashed with yellow to read colors in Fig, 4 (see color bar) regions between 50N and 90 bellow 10hPa. We have added how to localize the warming and cooling regions from the plots in the the figure-captions.

Lines 168-169: "This cooling explains the mid-to-high latitudes mesospheric temperature reduction."

I don't think there is a close correspondence between the changes in residual circulation and the changes in temperature. For example, the former is strongest at about 50-60N, whereas the latter is strongest at 90N.

We have explained how to read the residual circulation and adiabatic temperature changes in the figure captions. The cooling should not be collocated with the maximum of the residual circulation. Instead, cooling (warming) occurs in the upward (downward) branches of the residual circulation, which are located poleward of the maximum negative (positive) residual circulation.

Lines 169-170: "The overall vertical structure of the zonal wind response remains consistent with the meridional gradient of adiabatic temperature-changes,"

Again, this is not as obvious as the authors thought.

This consistency happens just because of the thermal wind balance; we now make it clearer by starting with "according to thermal wind balance ". The thermal wind balance is basic low of atmospheric and ocean dynamics that we tried to explain in the paper. We now explained it in more details by making clearer that the vertical shear of the zonal wind is proportional the negative meridional temperature gradient.

Lines 172-174: "RWF emerges as the primary driver of the stratospheric BDC-response in response to LSCO (Fig. 4b-f), intensifying its subsidence branch and inducing stronger adiabatic warming in mid-to-high latitudes."

"primary"? Panel b does not resemble panel a. Also, panel e does not resemble panel d.

The BDC is a stratospheric circulation. We wrote explicitly "stratospheric BDC response" to make this clear. In our case, it applies approximately below 1–2 hPa. We now state explicitly the vertical levels we refer to, namely between 150 hPa and about 2 hPa, to avoid ambiguity.

The uppermost stratospheric and mesospheric circulation is distinct from the BDC. It is commonly referred to as the Murgatroyd–Singleton circulation (MSC) and is interhemispheric, with an upward branch in the polar region of the summer hemisphere and a downward branch in the polar region of the winter hemisphere. In contrast, the BDC is hemispheric and does not reverse its direction between winter and summer. Unlike the MSC, which undergoes a complete seasonal reversal, the BDC primarily weakens from winter to summer.

Knowing that the BDC is confined to the stratosphere and that we therefore refer to the lower levels (bellow 2hPa), we can clearly identify the dominance of resolved wave forcing (RWF) in both the residual circulation and the associated adiabatic temperature changes.
The poleward residual circulation (bellow 2 hPa) seen in Fig. 4a and associated adiabatic warming seen in Fig. 4d are clearly reproduced in Fig. 4b and 4e, but not in Fig. 4c and 4f. This indicates that the changes in the BDC and associated adiabatic Temperature response seen in Fig. 4a and 4d are maintained to first order by RWF and not NRF.

Response in the paper:
We now add explicitely "between 150 and about 2 hP" to avoid confusion.

Lines 186-187, "The midlatitude SST-fronts exert significant influence on SSWs and PPSCs (Figs. 1&2), although their effects are weaker than those driven by LSCO (Fig. 4)."

This appears to be true, as suggested by the figures, but is not supported by the table. According to Supplementary Table 1, if one compares LSCO_NoFr and LSCO_Fr, one may conclude that the largest changes in SSW frequency are caused by adding the SST-fronts.

The way we wrote it is confusing. Thanks for pointing this out. What we intended to state is that although the effects of SST fronts on the mean stratospheric warming and vortex weakening are weaker than those driven by LSCO (Fig. 3), SST fronts still play a crucial role in increasing SSW and PPSC frequency.

When comparing Fig. 3a with Fig. 3b and Fig. 3c with Fig. 3d, we can clearly see that the LSCO effect on temperature and zonal wind is stronger (please note the different color-bar scales, which are now clarified in the caption). LSCO performs most of the work in warming the stratosphere and weakening the polar vortex. However, LSCO alone is not sufficient to reproduce the observed SSW and PPSC frequencies.

When SST fronts are added on top of LSCO, they act on an already weakened vortex and enhanced stratospheric warming, making wind reversals more likely and thereby increasing the frequency of SSWs. In contrast, as our experiments show, SST fronts alone (i.e., in the aqua-planet configuration without LSCO) are unable to sufficiently weaken the vortex, reverse the zonal winds, or generate SSWs.

Changes in the paper.

We have now changed the text to although their effects on the mean stratospheric warming, vortex weakening and therir dynamics are weaker than those driven by LSCO (Fig. 3 and Fig. 4).

We also made clearer the differences in the scales of the color bars of T, U and forcings in the captions between the response to SST-fronts and LSCO

Lines 233-234: "The Pacific SST-front emerges as the dominant driver of these changes, while the Atlantic SST-fronts exert minor impact. This underscores the importance of basin-specific SST-gradients."

Why? Any dynamic explanation?

Yes. The Pacific SST front induces a deepening of the North Pacific low geopotential height, which is a well-known tropospheric precursor for stratospheric vortex weakening and SSWs. This mechanism has been discussed in detail in several sections of the manuscript, supported by additional figures and references (see response to previous comments).

The deepening of the North Pacific low geopotential height acts to enhance upward planetary-wave propagation from the troposphere into the stratosphere. We have now explicitly quantified this upward wave propagation and diagnosed the associated tropospheric circulation response. These results are included in the revised manuscript and in our response to Reviewer 1, with supporting analyses shown in SFig. 8 and SFig. 9 (Lines 268–288 and 322–328).

In contrast, the Atlantic SST front does not produce a comparable tropospheric precursor pattern and therefore exerts a much weaker influence on upward wave propagation and stratospheric variability.

Lines 76-77 vs. Lines 314-316: In lines 76-77 "In AP_Fr a symmetric SST-front is introduced in AP_NoFr-configuration"; in lines 314-316: "The Aqua-Planet with SST-fronts (AP_Fr) experiment adds zonally averaged SST-fronts over the western Atlantic boundary current to the AP_NoFr-configuration,"

I don’t think these two statements are identical. What kind of SST-fronts are indeed used?

It should be “zonally symmetric SST-front” in both places.
In general, it is difficult to imposee observed zonally assymetric SST-front of the Atlantic and Pacific in aqua planet, as this will lead to unrealistic zonal SST-gradients. In the AP_NoFr-configuration, SST-fronts are removed by taking linear meridional decrease of the zonally averaged SST with constant gradient (ie. Constant SST-slope, see Sfig. 1). This Constant gradient is choosen within the range of the lowest SST-grdien North of 33.5N. This Methode has been widely used in previous papers (See references in the paper).

To keep the aquaplanet front experiment consistent with the non-front Aqua-planet configuration, we take the zonally averaged SST-front associated with western Atlantic boundary current and apply it uniformly in longitude. As a result, the SST field has **no zonal variation** in either AP_NoFr (SFig1a) or AP_Fr. This ensures that the difference AP_Fr – AP_NoFr isolates **only the effect of the meridional SST gradient associated with SST fronts**, without introducing zonal SST asymmetry or zonal SST gradients. This approach is standard in semi-idealized aqua-planet experiments designed to study SST-front effects and has been adopted in several previous studies (see references in the paper).

Changes in the paper:

We add zonally symmetric instead of just symmetric SS-fronts
We also have added some details in choice of SST-gradients in the method.

Fig. 3 b&d: the two panels represent the impacts of SST fronts in the presence of land-sea thermal contrast and orography. Can you also show the difference between AP_Fr and AP_NoFr, which represent the impacts of SST fronts in the absence of land-sea thermal contrast and orography? This also applies to the entire Fig. 5: you can also show the differences between

AP_Fr and AP_NoFr, which also represent the impacts of SST fronts but in the absence of land-sea thermal contrast and orography.

In our experiments, the aqua-planet (AP) configuration is designed to mimic Southern Hemisphere–like conditions. We did not show the difference **AP_Fr − AP_NoFr** because we know that in the Southern Hemisphere, even in the presence of SST fronts, there are almost no SSWs. This behavior is consistent with both observations and our aqua-planet results. When adding SST fronts in the aqua-planet configuration, there is no change in SSW frequency.

Therefore, we did not see the necessity to show the effect of SST fronts on temperature and zonal wind in the absence of LSCO, since our results demonstrate no significant impact of SST fronts in the AP configuration on SSWs and PPSC occurrence. Their impact is mainly seen in the variability of temperature and zonal wind rather than in the mean state or SSW statistics.

We have already added several important figures to the Supplementary Material in response to this and other reviewer comments. Adding further figures showing weak responses in the aqua-planet configuration would substantially increase the number of supplementary plots without providing additional insight. We therefore consider that the weak response of SSWs and PPSCs to SST fronts in the aqua-planet configuration makes a more detailed analysis of the stratospheric response in this configuration less relevant for the main conclusions of the paper.

References for R3

J. G. Charney, P. G. Drazin, Propagation of Planetary-Scale Disturbances from the Lower into the Upper Atmosphere. *Journal of Geophysical Research* 66, 83-109 (1961).
J. G. Charney, The Dynamics of Long Waves in a Baroclinic Westerly Current. *J Meteorol* 4, 135-162 (1947).
E. T. Eady, Long Waves and Cyclone Waves. *Tellus* 1, 33-52 (1949).
C. I. Garfinkel, D. L. Hartmann, Different ENSO teleconnections and their effects on the stratospheric polar vortex. *J Geophys Res-Atmos* 113, (2008).
C. I. Garfinkel, A. H. Butler, D. W. Waugh, M. M. Hurwitz, L. M. Polvani, Why might stratospheric sudden warmings occur with similar frequency in El Nino and La Nina winters? *J Geophys Res-Atmos* 117, D19106 (2012).
K. Nishii, H. Nakamura, T. Miyasaka, Modulations in the planetary wave field induced by upward-propagating Rossby wave packets prior to stratospheric sudden warming events: A case-study. *Q J Roy Meteor Soc* 135, 39-52 (2009).
S. Ineson, A. A. Scaife, The role of the stratosphere in the European climate response to El Nino. *Nat Geosci* 2, 32-36 (2009).
N.-E. Omrani *et al.*, Key Role of the Ocean Western Boundary currents in shaping the Northern Hemisphere climate. *Sci Rep-Uk* 9, 3014 (2019).
T. Sampe, H. Nakamura, A. Goto, Potential Influence of a Midlatitude Oceanic Frontal Zone on the Annular Variability in the Extratropical Atmosphere as Revealed by Aqua-Planet Experiments. *J Meteorol Soc Jpn* 91A, 243-267 (2013).
T. Sampe, H. Nakamura, A. Goto, W. Ohfuchi, Significance of a Midlatitude SST Frontal Zone in the Formation of a Storm Track and an Eddy-Driven Westerly Jet. *J Climate* 23, 1793-1814 (2010).

Drdla, K. (2010). The role of polar stratospheric clouds in Arctic ozone depletion. *Atmospheric Chemistry and Physics*, **10**(16), 7515–7531.
Poole, L. R., & McCormick, M. P. (1988). Airborne lidar observations of Arctic polar stratospheric clouds: Signature of small, solid particles above the frost point. *Geophysical Research Letters*, 15(8), 867–870.
Peter, T. (1997). Microphysics and heterogeneous chemistry of polar stratospheric clouds. *Annual Review of Physical Chemistry*, 48(1), 785–822.Rex, M., Salawitch, R. J., von der Gathen, P., Harris, N. R. P., Chipperfield, M. P., & Naujokat, B. (2004). Arctic ozone loss and climate change. *Geophysical Research Letters*, 31(4).Rex, M., Salawitch, R. J., von der Gathen, P., Harris, N. R. P., Chipperfield, M. P., & Naujokat, B. (2006). A long-term limiting value for Arctic ozone loss. *Nature Geoscience*, 1, 812–815.
Manney, G. L., Daffer, W. H., Pawson, S., O'Neill, A., & Naujokat, B. (2011). Unprecedented Arctic ozone loss in 2011. *Nature*, 478(7370), 469–475.
Solomon, S., Garcia, R. R., Rowland, F. S., & Wuebbles, D. J. (1986). On the depletion of Antarctic ozone. *Nature*, 321(6072), 755–758.
Hanson, D. R., & Ravishankara, A. R. (1994). Heterogeneous chemistry of HCl and $ClONO_2$ on sulfuric acid tetrahydrate and the polar stratospheric clouds. *Journal of Geophysical Research*, 99(D4), 8119–8127.
Tabazadeh, A., Toon, O. B., Schoeberl, M. R., Rocca, R., & Lopez, J. P. (2000). New insights into the mechanisms of PSC formation, processing, and denitrification. *Science*, 291(5512), 2591–2596.

Response to Reviewer #2

We thank the reviewer for the careful reading of the manuscript and for the positive evaluation. All remaining minor comments have been addressed as indicated below.

L3: MSSW; You should choose MSSW/SSW and use it thorough the paper, SSW is a better option since it's the standard.

Done.

L30: Majore -> Major

Done.

L31: 50C is quite too specific and it's not part of the definition; rapid warming could be enough here.

Done.

L57–58: This sentence leads to the understanding the BDC is responsible for the different polar vortex strengths between the Northern and the Southern Hemispheres. Please clarify.

Done.

L55–57: These three lines need some revision, it feels choppy, you could try to connect them.

Done.

L60: wavea -> wave

Done.

L64: Please, correct: "tropical (mid-to high) latitudes"

Done.

L100: In AP_Fr -> For AP_Fr

Done.

L101: in (the) AP_NoFr

Done.

L120: srong -> strong

Done.

L141 and 142: PPSC is already defined in L131.

Done.

L159: Figure R1Fig6 seems to be in agreement with Palmeiro et al. (2025).

Done.

L174: proportional “to”

Done.

L200: accelerate of -> accelerate

Done.

L200: reduces -> reduce

Done.

L244: “shifting stratospheric dynamics”; Shifting where?

Done.

L253: high-latitudes -> high-latitude

Done.

L320: use of “too strong”; this sentence could be improved

Done.

L321: “but not enough…” -> but insufficient to account for the observed…

Done.

L323: upward propagation -> upward wave propagation

Done.

L364: theire -> their

Done.
